# Mathematical model predicts anti-adhesion–antibiotic–debridement combination therapies can clear an antibiotic resistant infection

**DOI:** 10.1371/journal.pcbi.1007211

**Published:** 2019-07-23

**Authors:** Paul A. Roberts, Ryan M. Huebinger, Emma Keen, Anne-Marie Krachler, Sara Jabbari

**Affiliations:** 1 School of Mathematics, University of Birmingham, Edgbaston, Birmingham, United Kingdom; 2 Institute of Microbiology and Infection, School of Biosciences, University of Birmingham, Edgbaston, Birmingham, United Kingdom; 3 Department of Surgery, University of Texas Southwestern Medical Center, Dallas, Texas, United States of America; 4 Department of Microbiology and Molecular Genetics, University of Texas McGovern Medical School at Houston, Houston, Texas, United States of America; Oxford, UNITED KINGDOM

## Abstract

As antimicrobial resistance increases, it is crucial to develop new treatment strategies to counter the emerging threat. In this paper, we consider combination therapies involving conventional antibiotics and debridement, coupled with a novel anti-adhesion therapy, and their use in the treatment of antimicrobial resistant burn wound infections. Our models predict that anti-adhesion–antibiotic–debridement combination therapies can eliminate a bacterial infection in cases where each treatment in isolation would fail. Antibiotics are assumed to have a bactericidal mode of action, killing bacteria, while debridement involves physically cleaning a wound (e.g. with a cloth); removing free bacteria. Anti-adhesion therapy can take a number of forms. Here we consider adhesion inhibitors consisting of polystyrene microbeads chemically coupled to a protein known as multivalent adhesion molecule 7, an adhesin which mediates the initial stages of attachment of many bacterial species to host cells. Adhesion inhibitors competitively inhibit bacteria from binding to host cells, thus rendering them susceptible to removal through debridement. An ordinary differential equation model is developed and the antibiotic-related parameters are fitted against new *in vitro* data gathered for the present study. The model is used to predict treatment outcomes and to suggest optimal treatment strategies. Our model predicts that anti-adhesion and antibiotic therapies will combine synergistically, producing a combined effect which is often greater than the sum of their individual effects, and that anti-adhesion–antibiotic–debridement combination therapy will be more effective than any of the treatment strategies used in isolation. Further, the use of inhibitors significantly reduces the minimum dose of antibiotics required to eliminate an infection, reducing the chances that bacteria will develop increased resistance. Lastly, we use our model to suggest treatment regimens capable of eliminating bacterial infections within clinically relevant timescales.

## Author summary

Since the development of the first antibiotics, bacteria have utilised and developed resistance mechanisms, helping them to avoid being eliminated and to survive within a host. Traditionally, the solution to this problem has been to treat with multiple antibiotics, switching to a new type when the one currently in use proves ineffective. However, the development of antibiotics has slowed significantly in the past two decades, while multi-drug resistant strains, otherwise known as ‘super bugs’, are on the rise. In answer to this challenge, alternative approaches, such as anti-adhesion therapy, are being developed as a complement or alternative to traditional antimicrobials. In this paper we formulate and analyse a mathematical model of a combination therapy, applied in the context of an infected burn wound, bringing together antibiotics, anti-adhesion therapy and debridement (the physical cleaning of a wound). We use our models to make sense of how these treatments interact to combat a bacterial infection, to predict treatment outcomes for a range of strategies and to suggest optimal treatment regimens. It is hoped that this study will guide future experimental and clinical research, helping biomedical researchers to identify the most promising approaches to treatment.

## Introduction

Antimicrobial resistance (AMR) is on the rise [1–3] and with it the need to develop and apply novel treatment strategies [4, 5]. In this paper, we formulate and analyse mathematical models of combination therapies, bringing together traditional antibiotics and debridement with a new anti-adhesion treatment, seeking to determine if a combination therapy could succeed in eliminating an AMR infection in cases where antibiotics alone would fail.

It has been predicted that unless steps are taken to combat its rise, AMR could lead to as many as 10 million deaths per annum by the year 2050 [1]. Antibiotics are the standard treatment option for microbial infections. They may be classified into two broad categories: bactericidal and bacteriostatic [6]. Bactericidal antibiotics act by killing bacteria, while bacteriostatic antibiotics inhibit their growth (we note that some antibiotics may exhibit both modes of action). While effective in general, antibiotic use has the unfortunate consequence of selecting for those members of a bacterial population which are resistant to the antibiotic being applied. Resistance then spreads through the bacterial population via vertical (parent to daughter) and/or horizontal (cell to cell) gene transfer, until the resistant phenotype comes to dominate [7–9]. One solution to this problem is to use multiple antibiotics; however, this runs the risk of selecting for multi-drug resistant bacteria, or ‘super bugs’ [10]. An alternative approach is to use a class of treatments known as anti-virulence therapies, either in place of, or in addition to, antibiotics. Anti-virulence therapies are diverse [11–13]; however, they have the common aim of preventing or limiting disease in the host [6]. By using these therapies in combination with more traditional treatments, such as antibiotics and debridement (physical clearance of a wound e.g. with a cloth), it is hoped that bacteria can be cleared from a host more rapidly, while reducing the risk of resistant phenotypes emerging [14, 15].

In this paper we shall consider a particular form of anti-virulence therapy, known as anti-adhesion therapy, which operates by preventing bacteria from binding to the cells of an infected host, thus rendering them more susceptible to physical clearance [13]. Krachler *et al*. [12] have developed an anti-adhesion treatment based upon a protein, discovered earlier by the same group, which they named multivalent adhesion molecule (MAM) 7 [16]. MAM7 is anchored in the extracellular side of the outer membrane of many Gram-negative bacteria, where it is responsible for mediating the initial stages of attachment to host cells [16, 17]. By chemically coupling polystyrene microbeads to MAM7, adhesion inhibitors (henceforth inhibitors) can be constructed which, when applied to an infection site, competitively inhibit the binding of bacteria to host cells [18]. Burn wound infections provide a promising application of this treatment [18–20]. Nosocomial (hospital-acquired) infections pose a major challenge in the treatment of burn wound patients, as these wounds create a significant opportunity for bacteria to penetrate host defences [21–25]. Here we consider the potential of an anti-adhesion–antibiotic–debridement combination therapy to clear an infection, preventing further tissue damage and sepsis.

The mathematical model developed in the present study extends our earlier model in Roberts *et al*. [26], which considered the response of a purely susceptible bacterial infection to treatment with inhibitors and debridement. Our models predicted that, when combined with debridement, the bacterial burden could be significantly reduced and, in some cases, eliminated. The present study extends this model by considering mixed susceptible and resistant infections and an augmented treatment strategy, combining inhibitors and debridement with antibiotics. This is the first mathematical modelling study: (i) to consider the effects of antibiotic in a situation where bacteria can exist in either bound or unbound states in the absence of a biofilm ([27] and [28], noted below, do not include antibiotic treatment); (ii) to consider a treatment combining antibiotics with anti-adhesion therapy, or (iii) to predict optimal antibiotic-inhibitor-debridement treatment regimens.

As in [26], the present mathematical model is based upon the *in vivo* rat burn wound model described in Huebinger *et al*. [18]. In each experiment, a burn wound was administered to the back of a rat and a portion of the resulting necrotic tissue later excised. An inoculum of the Gram-negative *Pseudomonas aeruginosa* (*P. aeruginosa*) bacteria was then applied to the wound, together with an active or inactive form of the inhibitor. The bacterial burden was monitored for six days, after which each rat was euthanised (see [18] for further details). The treatment was found to effect a marked reduction in the total bacterial burden compared to controls.

A mathematical model of a generalised anti-virulence treatment combined with antibiotics was proposed by Ternant *et al*. [29]. This ODE model conceived of anti-virulence treatment as providing a boost to the immune system, though it did not consider an anti-adhesion therapy specifically. The model predicted that antibiotics and anti-virulence treatments could be effective when used in combination, in cases where neither is effective in isolation, provided the therapies are administered in staggered doses. A number of modelling studies have considered bacterial infections of burn wounds [30–35], the binding of bacteria to surfaces [27, 28] and anti-virulence treatments which interfere with quorum sensing [33, 36–42]. There is also a large literature on the mathematical modelling of antibiotic therapy (see [43–48] for reviews).

In this paper we develop an ODE model to describe and predict the bacterial population dynamics in an infected burn wound, under treatment regimens combining antibiotic, inhibitor and debridement therapies. We fit our antibiotic-associated parameters to new *in vitro* data collected for this study. We use our models to gain insight into how these combination therapies operate, to predict treatment outcomes and to suggest ways in which therapy could be optimised in a clinical setting. Crucially, it is found that anti-adhesion–antibiotic-debridement combination therapies can eliminate bacterial infections in situations where each treatment would fail when used in isolation.

## Materials and methods

### Model development

**Fig 1:**
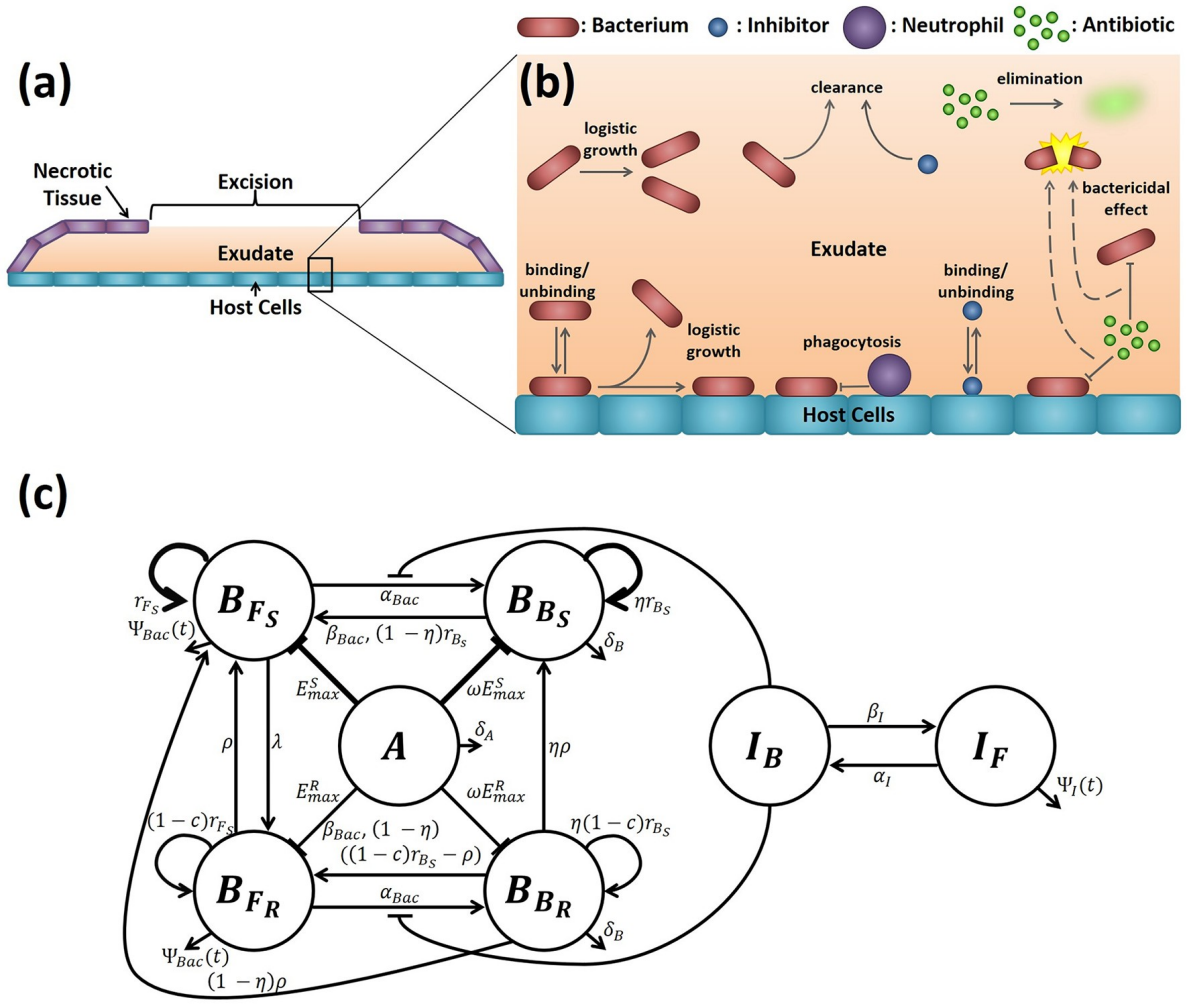
Wound geometry and model structure. (a) The wound is pictured in the transverse plane. The (liquid) exudate overlies the host cells, while a layer of necrotic tissue covers the wound, except at the excision where the exudate is exposed to the air. (b) The processes described in the mathematical model as they relate to the wound geometry (the diagram does not distinguish between susceptible and resistant bacteria, nor does it depict conjugation or segregation). (c) A representation of the model topology (Eqs 1–11). $B_{F_{S}}$: free susceptible bacteria, $B_{F_{R}}$: free resistant bacteria, $B_{B_{S}}$: bound susceptible bacteria, $B_{B_{R}}$: bound resistant bacteria, *I*_*F*_: free inhibitors, *I*_*B*_: bound inhibitors, and *A*: antibiotic. Antibiotic directly inhibits bacteria by killing them, while inhibitors indirectly inhibit bacteria by limiting their ability to bind to host cells. The intrinsic growth rate of susceptible bacteria is greater than that of resistant bacteria, while the maximum killing rate of susceptible bacteria by antibiotic is greater than that for resistant bacteria. This is represented by thicker arrows/inhibition symbols in each case. See Table 1 for variable descriptions and units, and Tables 2–4 for parameter descriptions and units.

We construct a mathematical model of an infected burn wound, focussing upon the bacterial population and the treatment strategies employed to clear an infection. For our purposes a burn wound consists of a layer of host cells, over which lies a fluid layer, exuded by the host cells, called the exudate. The exudate is partially covered by a layer of necrotic tissue, except in the region of a surgical excision, where it is exposed to the air and from which fluid may leak. If left undisturbed, a scab forms across the excision after 24 hr, preventing further fluid loss (see Fig 1(a)). The environment-dependent parameters used in this paper were fitted to an *in vivo* rat model with the bacterial species *P. aeruginosa* in [26]; however, this model is also of relevance to burn wounds in humans and for any bacterial species for which host cell attachment is partly mediated by MAM7. See the ‘Experimental set-up’ and ‘Model formulation’ sections of [26] for more details.

Our model considers three types of species: bacteria, inhibitors and antibiotics. Both bacteria and inhibitors may exist in one of two physical states, either swimming/floating freely in the exudate or bound to the host cells, while antibiotics remain in solution in the exudate at all times. Further, bacteria come in two varieties: those which are more vulnerable to antibiotic (susceptible bacteria) and those which have developed resistance to the antibiotic (resistant bacteria). In this study we model a bactericidal antibiotic, employing parameter values fitted to newly measured *in vitro* kill curves for *P. aeruginosa* using the antibiotic meropenem (see Parameter fitting and justification and S1 Text for more details). Meropenem is commonly used to treat *P. aeruginosa* burn wound infections [49] and can be administered intravenously; thus, it is a natural choice for this study. Inhibitors are applied directly to the exudate, whereas antibiotics are applied systemically, entering the wound through the host cell layer, having reached the wound via the bloodstream.

Assuming, as in [26], that the system is well-mixed, we define an ordinary differential equation (ODE) model for free susceptible bacteria density, $B_{F_{S}}\left(t\right)$ (cells cm^−3^), free resistant bacteria density, $B_{F_{R}}\left(t\right)$ (cells cm^−3^), bound susceptible bacteria density, $B_{B_{S}}\left(t\right)$ (cells cm^−2^), bound resistant bacteria density, $B_{B_{R}}\left(t\right)$ (cells cm^−2^), free inhibitor concentration, *I*_*F*_(*t*) (inhib. cm^−3^), bound inhibitor concentration, *I*_*B*_(*t*) (inhib. cm^−2^), and antibiotic concentration *A*(*t*) (*μ*g cm^−3^), over time, *t* (hr) (the dependent and independent variables are summarised in Table 1). It is assumed that the total binding site density on the host cells, consisting of both free and occupied sites, is conserved, such that the free binding site density $E\left(t\right)=E_{total}-\phi_{Bac}\left(B_{B_{S}}\left(t\right)+B_{B_{R}}\left(t\right)\right)-\phi_{I}I_{B}\left(t\right)$ (sites cm^−2^), where *E*_*total*_ (sites cm^−2^) is the total density of binding sites (both free and bound), and *ϕ*_*Bac*_ (sites cell^−1^) and *ϕ*_*I*_ (sites inhib.^−1^) are the number of binding sites occupied by a bacterium or an inhibitor respectively.

**Table 1:** Dependent and independent variables for Eqs 1–11.

Variable	Description (Units)
$B_{F_{S}}$	Free susceptible bacteria density (cells cm^−3^)
$B_{F_{R}}$	Free resistant bacteria density (cells cm^−3^)
$B_{B_{S}}$	Bound susceptible bacteria density (cells cm^−2^)
$B_{B_{R}}$	Bound resistant bacteria density (cells cm^−2^)
*I*_*F*_	Free inhibitor concentration (inhib. cm^−3^)
*I*_*B*_	Bound inhibitor concentration (inhib. cm^−2^)
*A*	Antibiotic concentration (*μ*g cm^−3^)
*t*	Time (hr)

The model, summarised in Fig 1(b) and 1(c), is described by the following governing equations

$$\begin{array}{cc} \frac{dB_{F_{S}}}{dt} & =[\underset{\text{logistic}\;\text{growth}}{\underset{︸}{r_{F_{S}}B_{F_{S}}H\left(VB_{F_{S}}-1\right)}}+\underset{\text{segregation}}{\underset{︸}{\rho B_{F_{R}}H\left(VB_{F_{R}}-1\right)H\left(K_{F}-B_{F_{S}}-B_{F_{R}}\right)}}](1-\frac{B_{F_{S}}+B_{F_{R}}}{K_{F}})\\ & \;+\underset{\text{daughter}\;\text{cells}\;\text{freed}\;\text{from}\;\text{host}\;\text{cells}\;\text{upon}\;\text{division}}{\underset{︸}{\left(1-\eta \left(E\right)\right)H\left(K_{B}-B_{B_{S}}-B_{B_{R}}\right)}}\\ & \;\times \frac{1}{h}[\underset{\text{logistic}\;\text{growth}}{\underset{︸}{r_{B_{S}}B_{B_{S}}H\left(A_{r}B_{B_{S}}-1\right)}}+\underset{\text{segregation}}{\underset{︸}{\rho B_{B_{R}}H\left(A_{r}B_{B_{R}}-1\right)}}](1-\frac{B_{B_{S}}+B_{B_{R}}}{K_{B}})\\ & \;-\underset{\text{binding}\;\text{to}\;\text{host}\;\text{cells}}{\underset{︸}{\alpha_{Bac}A_{r}B_{F_{S}}E}}+\underset{\text{unbinding}\;\text{from}\;\text{host}\;\text{cells}}{\underset{︸}{\frac{\beta_{Bac}}{h}B_{B_{S}}}}-\underset{\text{killing}\;\text{by}\;\text{antibiotic}}{\underset{︸}{\frac{E_{max}^{S}A}{A_{50}^{S}+A}B_{F_{S}}}}-\underset{\text{conjugation}}{\underset{︸}{\lambda B_{F_{S}}B_{F_{R}}}}-\underset{\text{natural}\;\text{clearance}}{\underset{︸}{\psi_{Bac}\left(t\right)B_{F_{S}}}}\text{,} \end{array}$$ (1)

$$\begin{array}{cc} \frac{dB_{F_{R}}}{dt} & =[\underset{\text{logistic}\;\text{growth}}{\underset{︸}{\left(1-cH\left(K_{F}-B_{F_{S}}-B_{F_{R}}\right)\right)r_{F_{S}}B_{F_{R}}}}-\underset{\text{segregation}}{\underset{︸}{\rho B_{F_{R}}H\left(K_{F}-B_{F_{S}}-B_{F_{R}}\right)}}]\\ & \;\times (1-\frac{B_{F_{S}}+B_{F_{R}}}{K_{F}})H\left(VB_{F_{R}}-1\right)\\ & \;+\underset{\text{daughter}\;\text{cells}\;\text{freed}\;\text{from}\;\text{host}\;\text{cells}\;\text{upon}\;\text{division}}{\underset{︸}{\left(1-\eta \left(E\right)\right)H\left(K_{B}-B_{B_{S}}-B_{B_{R}}\right)}}\\ & \;\times \frac{1}{h}[\underset{\text{logistic}\;\text{growth}}{\underset{︸}{\left(1-c\right)r_{B_{S}}B_{B_{R}}}}-\underset{\text{segregation}}{\underset{︸}{\rho B_{B_{R}}}}](1-\frac{B_{B_{S}}+B_{B_{R}}}{K_{B}})H\left(A_{r}B_{B_{R}}-1\right)\\ & \;-\underset{\text{binding}\;\text{to}\;\text{host}\;\text{cells}}{\underset{︸}{\alpha_{Bac}A_{r}B_{F_{R}}E}}+\underset{\text{unbinding}\;\text{from}\;\text{host}\;\text{cells}}{\underset{︸}{\frac{\beta_{Bac}}{h}B_{B_{R}}}}-\underset{\text{killing}\;\text{by}\;\text{antibiotic}}{\underset{︸}{\frac{E_{max}^{R}A}{A_{50}^{R}+A}B_{F_{R}}}}+\underset{\text{conjugation}}{\underset{︸}{\lambda B_{F_{S}}B_{F_{R}}}}-\underset{\text{natural}\;\text{clearance}}{\underset{︸}{\psi_{Bac}\left(t\right)B_{F_{R}}}}\text{,} \end{array}$$ (2)

$$\begin{array}{cc} \frac{dB_{B_{S}}}{dt} & =\underset{\text{a}\;\text{proportion,}\;\eta \text{,}\;\text{remain}\;\text{attached}}{\underset{︸}{\left[1+\left(\eta \left(E\right)-1\right)H\left(K_{B}-B_{B_{S}}-B_{B_{R}}\right)\right]}}\\ & \;\times [\underset{\text{logistic}\;\text{growth}}{\underset{︸}{r_{B_{S}}B_{B_{S}}H\left(A_{r}B_{B_{S}}-1\right)}}+\underset{\text{segregation}}{\underset{︸}{\rho B_{B_{R}}H\left(A_{r}B_{B_{R}}-1\right)H\left(K_{B}-B_{B_{S}}-B_{B_{R}}\right)}}]\\ & \;\times (1-\frac{B_{B_{S}}+B_{B_{R}}}{K_{B}})+\underset{\text{binding}\;\text{to}\;\text{host}\;\text{cells}}{\underset{︸}{\alpha_{Bac}VB_{F_{S}}E}}-\underset{\text{unbinding}\;\text{from}\;\text{host}\;\text{cells}}{\underset{︸}{\beta_{Bac}B_{B_{S}}}}-\underset{\text{phagocytosis}}{\underset{︸}{\delta_{B}B_{B_{S}}}}-\underset{\text{killing}\;\text{by}\;\text{antibiotic}}{\underset{︸}{\omega \frac{E_{max}^{S}A}{A_{50}^{S}+A}B_{B_{S}}}}\text{,} \end{array}$$ (3)

$$\begin{array}{cc} \frac{dB_{B_{R}}}{dt} & =\underset{\text{a}\;\text{proportion,}\;\eta \text{,}\;\text{remain}\;\text{attached}}{\underset{︸}{\left[1+\left(\eta \left(E\right)-1\right)H\left(K_{B}-B_{B_{S}}-B_{B_{R}}\right)\right]}}\\ & \;\times [\underset{\text{logistic}\;\text{growth}}{\underset{︸}{\left(1-cH\left(K_{B}-B_{B_{S}}-B_{B_{R}}\right)\right)r_{B_{S}}B_{B_{R}}}}-\underset{\text{segregation}}{\underset{︸}{\rho B_{B_{R}}H\left(K_{B}-B_{B_{S}}-B_{B_{R}}\right)}}]\\ & \;\times (1-\frac{B_{B_{S}}+B_{B_{R}}}{K_{B}})H\left(A_{r}B_{B_{R}}-1\right)\\ & \;+\underset{\text{binding}\;\text{to}\;\text{host}\;\text{cells}}{\underset{︸}{\alpha_{Bac}VB_{F_{R}}E}}-\underset{\text{unbinding}\;\text{from}\;\text{host}\;\text{cells}}{\underset{︸}{\beta_{Bac}B_{B_{R}}}}-\underset{\text{phagocytosis}}{\underset{︸}{\delta_{B}B_{B_{R}}}}-\underset{\text{killing}\;\text{by}\;\text{antibiotic}}{\underset{︸}{\omega \frac{E_{max}^{R}A}{A_{50}^{R}+A}B_{B_{R}}}}\text{,} \end{array}$$ (4)

$$\begin{array}{cc} \frac{dI_{F}}{dt} & =-\underset{\text{binding}\;\text{to}\;\text{host}\;\text{cells}}{\underset{︸}{\alpha_{I}A_{r}I_{F}E}}+\underset{\text{unbinding}\;\text{from}\;\text{host}\;\text{cells}}{\underset{︸}{\frac{\beta_{I}}{h}I_{B}}}-\underset{\text{natural}\;\text{clearance}}{\underset{︸}{\psi_{I}\left(t\right)I_{F}}}\text{,} \end{array}$$ (5)

$$\begin{array}{cc} \frac{dI_{B}}{dt} & =\underset{\text{binding}\;\text{to}\;\text{host}\;\text{cells}}{\underset{︸}{\alpha_{I}VI_{F}E}}-\underset{\text{unbinding}\;\text{from}\;\text{host}\;\text{cells}}{\underset{︸}{\beta_{I}I_{B}}}\text{,} \end{array}$$ (6)

$$\begin{array}{cc} \frac{dA}{dt} & =\{\begin{array}{cc} -\underset{\text{elimination}}{\underset{︸}{\delta_{A}A}} & \text{discrete}\;\text{dosing,}\\ 0 & \text{constant}\;\text{concentration,} \end{array} \end{array}$$ (7)

where parameter definitions and values are given in Tables 2–4. See Parameter fitting and justification for details on how the parameter values were obtained.

**Table 2:** Parameter values fitted to *in vivo* data for Eqs 1–11.

Parameter	Description (Units)	Value			
		Case A	Case B	Case C	Case D
$r_{F_{S}}$	Intrinsic growth rate of free susceptible bacteria (hr^−1^)	8.37×10^−2^	3.97×10^−2^	5.57×10^−3^	2.57×10^−1^
$r_{B_{S}}$	Intrinsic growth rate of bound susceptible bacteria (hr^−1^)	1.10×10^−1^	1.60	8.81×10^−2^	5.55
*K*_*F*_	Carrying capacity of free bacteria (cells cm^−3^)	1.17×10^7^	8.23×10^6^	1.95×10^7^	1.85×10^6^
*K*_*B*_	Carrying capacity of bound bacteria (cells cm^−2^)	9.96×10^5^	4.15×10^5^	1.79×10^6^	1.43×10^6^
*α*_*Bac*_	Binding rate of bacteria to host cells (hr^−1^ sites^−1^)	1.34×10^−9^	1.88×10^−11^	6.47×10^−10^	3.34×10^−11^
*β*_*Bac*_	Unbinding rate of bacteria from host cells (hr^−1^)	1.97×10^−1^	2.02×10^−3^	2.48×10^−10^	5.79×10^−6^
*δ*_*B*_	Rate of phagocytosis of bacteria by neutrophils (hr^−1^)	1.06×10^−3^	1.90×10^−6^	2.95×10^−5^	3.02×10^−5^
*η*_*max*_	Maximum proportion of daughters of bound cells that can enter the bound compartment (dimensionless)	2.95×10^−2^	1.23×10^−8^	3.37×10^−2^	1.52×10^−2^
*γ*	Concentration of binding sites at which *η* = *η*_*max*_/2 (sites cm^−2^)	3.12×10^4^	1.89×10^5^	1.05×10^4^	1.65×10^6^
${\tilde{\psi }}_{Bac}$	Natural clearance rate of bacteria (hr^−1^)	1.42×10^−1^	7.28×10^−2^	1.39×10^−3^	5.01×10^−1^
*α*_*I*_	Binding rate of inhibitors to host cells (hr^−1^ sites^−1^)	1.46×10^−6^	1.77×10^−10^	6.47×10^−10^	5.51×10^−9^
*β*_*I*_	Unbinding rate of inhibitors from host cells (hr^−1^)	6.35×10^−8^	4.48×10^−6^	3.92×10^−3^	4.43×10^−1^
${\tilde{\psi }}_{I}$	Natural clearance rate of inhibitors (hr^−1^)	4.39×10^−8^	5.17×10^−4^	5.29×10^−3^	1.75×10^−5^

Values are given to an accuracy of 3 significant figures. All parameter values in this table are taken from [26].

**Table 3:** Parameter values fitted to *in vitro* data for Eqs 1–11.

Parameter	Description (Units)	Value
*c*	Fitness cost (dimensionless)	0.328
$E_{max}^{S}$	Maximum killing rate of susceptible bacteria by antibiotics (hr^−1^)	0.133
$E_{max}^{R}$	Maximum killing rate of resistant bacteria by antibiotics (hr^−1^)	8.62×10^−2^
$A_{50}^{S}$	Antibiotic concentration at which killing rate of susceptible bacteria is half maximal (*μ*g cm^−3^)	7.30×10^−2^
$A_{50}^{R}$	Antibiotic concentration at which killing rate of resistant bacteria is half maximal (*μ*g cm^−3^)	14.2

Values are given to an accuracy of 3 significant figures. All parameter values in this table were fitted as part of the present study as described in Parameter fitting and justification and S1 Text.

**Table 4:** Measured, calculated, literature-derived and estimated parameter values for Eqs 1–11.

Parameter	Description (Units)	Value	Source
*ϕ*_*Bac*_	Number of binding sites occupied by a bacterium (sites cell^−1^)	1	Estimated
*ϕ*_*I*_	Number of binding sites occupied by an inhibitor (sites inhib.^−1^)	1	Calculated
*V*	Volume of the exudate (cm^3^)	4.9	Calculated
*A*_*r*_	Area of the burn wound (cm^2^)	49	Measured
*h*	Height of the exudate (cm)	0.1	Measured
*n*	Hill coefficient (dimensionless)	1	Estimated
λ	Conjugation rate (cm^3^cell^−1^hr^−1^)	0 (10^−14^–10^−9^)	[50–53]
*ρ*	Segregation rate (hr^−1^)	0 (10^−4^)	[52, 54]
*δ*_*A*_	Elimination rate of antibiotic (hr^−1^)	0 or 1	[29, 55–58]
*ω*	Factor difference in antibiotic potency against bound bacteria compared with free bacteria (dimensionless)	1 (0.5–2)	Estimate
$B_{F_{S_{init}}}$	Initial density of free susceptible bacteria (cells cm^−3^)	1.00×10^6^	Estimated
$B_{F_{R_{init}}}$	Initial density of free resistant bacteria (cells cm^−3^)	2.04×10^4^	Estimated
$I_{F_{init}}$	Initial concentration of free inhibitors (inhib. cm^−3^)	0 or 6.12×10^7^	Measured
*A*_*init*_	Initial concentration of antibiotic (*μ*g cm^−3^)	0 or 8	[59–61]
*E*_*total*_	Total density of binding sites (sites cm^−2^)	2.57×10^6^	Calculated

Measured values are those which have been measured directly, calculated values are those which have been calculated using values which were measured directly, literature-derived values are taken directly from the literature and estimated values are those which could not be measured, calculated or obtained from the literature. Where multiple values are given, those without brackets are typical values, while those within brackets denote biologically realistic ranges (*λ* and *ρ*) or ranges used in sensitivity analyses (*ω*). Values are given to an accuracy of 3 significant figures.

We note that this model differs from that presented in [26] in the following respects:

- it includes antibiotic concentration, resulting in an additional ODE (Eq 7);
- both the free and bound bacterial compartments are split into susceptible and resistant sub-compartments (having been tacitly susceptible in [26]), such that the two ODEs describing free and bound bacterial dynamics are replaced with four ODEs for free susceptible, free resistant, bound susceptible and bound resistant bacteria (Eqs 1–4);
- it includes parameters and terms for fitness cost to resistant bacteria, conjugation, segregation, killing of bacteria by antibiotic and the factor difference in antibiotic potency against bound bacteria compared with free bacteria (see below for more details);
- it includes terms to prevent the regrowth of bacteria once their population size goes beneath one (see below).

These key extensions to our model in [26] facilitate investigation into how the combination therapies presented here can best be employed to tackle an otherwise untreatable antibiotic resistant infection.

Both free and bound bacteria are assumed to grow logistically with carrying capacities *K*_*F*_ (cells cm^−3^) and *K*_*B*_ (cells cm^−2^) respectively. In our model, the carrying capacities represent the maximum number of bacteria that can be sustained by available nutrients and are such that bacterial division is negligible when $B_{F_{S}}\left(t\right)+B_{F_{R}}\left(t\right)=K_{F}$ or $B_{B_{S}}\left(t\right)+B_{B_{R}}\left(t\right)=K_{B}$ (see [62, 63]). It is important to note that the number of bacteria that can be supported by nutrients near the host cells is not in general equal to the number of available binding sites on the host cells (*K*_*B*_ ≠ *E*_*total*_/*ϕ*_*Bac*_), indeed *K*_*B*_ < *E*_*total*_/*ϕ*_*Bac*_ for all parameter sets considered here (see Tables 2 and 4).

Susceptible bacteria have intrinsic growth rates $r_{F_{S}}$ (hr^−1^) (free) and $r_{B_{S}}$ (hr^−1^) (bound), while resistant bacteria incur a fitness cost, 0 < *c* < 1 (dimensionless), such that their intrinsic growth rates are $\left(1-c\right)r_{F_{S}}$ (hr^−1^) (free) and $\left(1-c\right)r_{B_{S}}$ (hr^−1^) (bound). This fitness cost only operates when the logistic terms represent bacterial growth. If the density of free cells, $B_{F_{S}}\left(t\right)+B_{F_{R}}\left(t\right)$, exceeds the free carrying capacity, *K*_*F*_, then the free logistic growth term becomes a death term, and likewise for bound bacteria. In this case the intrinsic growth rates of resistant bacteria revert to those of susceptible bacteria, since resistant bacteria are assumed to die at the same rate as susceptible bacteria. This is achieved through the use of Heaviside step functions, $H(K_{F}-B_{F_{S}}\left(t\right)-B_{F_{R}}\left(t\right))$ and $H(K_{B}-B_{B_{S}}\left(t\right)-B_{B_{R}}\left(t\right))$, in Eqs 2 and 4, where

$$\begin{array}{cc} H(x) & ≔\{\begin{array}{cc} 0 & \text{if}\;x<0\text{,}\\ 1 & \text{if}\;x\geq 0\text{.} \end{array} \end{array}$$ (8)

Further, the growth of any bacterial subtype (free-susceptible/free-resistant/bound-susceptible/bound-resistant) ceases once the number of bacteria in that subtype falls beneath one, since at least one cell is required in order for division to be possible. This is achieved using the Heaviside step functions $H(VB_{F_{S}}\left(t\right)-1)$, $H(VB_{F_{R}}\left(t\right)-1)$, $H(A_{r}B_{B_{S}}\left(t\right)-1)$ and $H(A_{r}B_{B_{R}}\left(t\right)-1)$, in Eqs 1–4, where *H* is defined in Eq 8.

Daughter cells derived from bound bacteria may enter either the bound compartment (in the proportion 0 ≤ *η*(*E*(*t*)) ≤ 1 (dimensionless)) or the free compartment (1 − *η*(*E*(*t*))), the proportion entering the bound compartment increasing as the density of free binding sites, *E*(*t*), increases. We model this dependence using a Hill function as follows

$$\begin{array}{c} \eta \left(E\right)=\frac{\eta_{max}E^{n}}{\gamma^{n}+E^{n}}\text{,} \end{array}$$ (9)

where *η*_*max*_ (dimensionless) is the maximum proportion of daughter cells which may remain bound to the surface, *γ* (sites cm^−2^) is the binding site density at which *η*(*E*) = *η*_*max*_/2 and *n* (dimensionless) is the Hill coefficient. We use a Heaviside step function, $H(K_{B}-B_{B_{S}}\left(t\right)-B_{B_{R}}\left(t\right))$, in Eqs 1–4 to restrict cell death due to the bound logistic growth term to the bound compartment when $B_{B_{S}}\left(t\right)+B_{B_{R}}\left(t\right)>K_{B}$, where *H* is defined in Eq 8.

The resistant strain of *P. aeruginosa* used in our *in vitro* experiments, PA1004 Evo10, transfers resistance genes vertically, but not horizontally. Therefore, throughout most of this study we neglect horizontal gene transfer and segregation. We include conjugation and segregation terms in Eqs 1–11 so as to make our model relevant to a wider class of infections, performing a sensitivity analysis on these parameters in Sensitivity analysis. In those cases where horizontal gene transfer does occur, resistant bacteria transfer plasmids conferring resistance to susceptible bacteria via conjugation at a rate *λ* (cm^3^cell^−1^hr^−1^). It is assumed that this process occurs within the free compartment, but not within the bound compartment or between the two compartments, since bound bacteria are typically physically separated from each other and free bacteria are unlikely to interact with bound bacteria. Horizontal gene transfer can also occur via transformation and transduction; however, we consider only conjugation here since it is the most common of the three mechanisms [10]. When a bacterium divides, its plasmids are segregated (divided) between the resulting daughter cells. A portion of the daughter cells of resistant bacteria produced upon division fail to inherit the resistance plasmid, leading to the production of susceptible offspring (by resistant bacteria) at a rate *ρ* (hr^−1^) (see [53] for an example). Similarly to the processes described above, segregation only occurs where the number of free or bound bacteria are below carrying capacity and where the number of free or bound resistant bacteria is greater than one. This is achieved through the use of Heaviside step functions, $H(K_{F}-B_{F_{S}}\left(t\right)-B_{F_{R}}\left(t\right))$, $H(K_{B}-B_{B_{S}}\left(t\right)-B_{B_{R}}\left(t\right))$, $H(VB_{F_{R}}\left(t\right)-1)$ and $H(A_{r}B_{B_{R}}\left(t\right)-1)$, in Eqs 1–4, where *H* is defined in Eq 8.

Bacteria and inhibitors bind to and unbind from the host cells with respective binding rates *α*_*Bac*_ (hr^−1^ sites^−1^) and *α*_*I*_ (hr^−1^ sites^−1^), and unbinding rates *β*_*Bac*_ (hr^−1^) and *β*_*I*_ (hr^−1^), in accordance with the law of mass action.

Neutrophils are present only on the surface of the host cells and are fully upregulated throughout an infection, such that bound bacteria can be assumed to decay exponentially at rate *δ*_*B*_ (hr^−1^), where *δ*_*B*_ accounts for neutrophil density.

We use Michaelis-Menten terms for the killing rates of susceptible and resistant bacteria by antibiotics to capture the saturating effects of increased antibiotic concentration. The maximum killing rates are given by $E_{max}^{S}$ (hr^−1^) (susceptible) and $E_{max}^{R}$ (hr^−1^) (resistant), where $E_{max}^{S}>E_{max}^{R}$, while the Michaelis constants $A_{50}^{S}$ (*μ*g cm^−3^) (susceptible) and $A_{50}^{R}$ (*μ*g cm^−3^) (resistant) give the antibiotic concentrations at which the killing rate is half-maximal, where $A_{50}^{R}>A_{50}^{S}$ (see Table 3). We multiply the bound bacteria antibiotic killing terms by a factor *ω* (dimensionless), to account for the potential difference in the antibiotic potency against bound bacteria as compared with free bacteria. Bound bacteria may be less vulnerable to antibiotic than free bacteria, in which case *ω* < 1; however, they may also be exposed to higher concentrations of antibiotic, which enters the wound through the host cell layer, in which case they may be more vulnerable, such that *ω* > 1. If bound bacteria are equally as vulnerable to antibiotic as free bacteria then *ω* = 1.

The clearance of bacteria and inhibitors (*ψ*_*Bac*_(*t*) (hr^−1^) and *ψ*_*I*_(*t*) (hr^−1^)) is assumed to occur at a constant rate for the first 24 hours, after which it ceases when a scab forms over the excision. Therefore, clearance occurs at rates

$$\begin{array}{c} \psi_{Bac}\left(t\right)={\tilde{\psi }}_{Bac}H\left(24-t\right)\;\text{and}\;\;\psi_{I}\left(t\right)={\tilde{\psi }}_{I}H\left(24-t\right)\text{,} \end{array}$$ (10)

where ${\tilde{\psi }}_{Bac}$ (hr^−1^) and ${\tilde{\psi }}_{I}$ (hr^−1^) are the constant clearance rates which apply in the first 24 hours, and *H* is a Heaviside step function (see Eq 8).

Antibiotics may either be administered in discrete doses or applied continuously, such that the antibiotic concentration remains fixed. In the former case, antibiotic is assumed to be eliminated from the system (e.g. through degradation and clearance into the bloodstream and surrounding tissues) at a rate *δ*_*A*_ (hr^−1^) following a dosing event. It is assumed that the loss of antibiotic through its interaction with bacteria is negligible in comparison to its elimination rate, and hence we do not include it in the model. Further, we do not include an antibiotic clearance term, similar to those given in Eq 10 for bacteria and inhibitors, since, while some antibiotic will leave the wound within the leaking exudate, this will not affect the antibiotic concentration in the remaining exudate (which is replenished via passage cross the host cell layer). It is assumed that inhibitor degradation, if it occurs, is sufficiently gradual that it can be neglected.

Several of the terms in Eqs 1–7 contain the exudate height, *h*, or volume, *V*, or the wound area, *A*_*r*_, as a factor in order to ensure dimensional consistency. We retain them in explicit form in the interests of clarity, though we note they could have been combined with their multipliers to create new parameters.

Bacteria are applied to the burn wound, following the excision, at time *t* = 0 (hr). This is also the first occasion upon which inhibitor or antibiotic treatment may be applied. Therefore, initially

$$\begin{array}{l} B_{F_{S}}\left(0\right)=B_{F_{S_{init}}},B_{F_{R}}\left(0\right)=B_{F_{R_{init}}},B_{B_{S}}\left(0\right)=0,B_{B_{R}}\left(0\right)=0,\\ I_{F}\left(0\right)=I_{F_{init}},I_{B}\left(0\right)=0,A\left(0\right)=A_{init}\text{,} \end{array}$$ (11)

where $B_{F_{S_{init}}}$, $B_{F_{R_{init}}}$, $I_{F_{init}}$ and *A*_*init*_ are constants. The bound compartments are empty initially, since bacteria and inhibitors have not had an opportunity to bind to the host cells. See Tables 2–4 for parameter values. We note that we retain equations in dimensional form to ease biological interpretation.

### Treatment types

Previously we considered a susceptible only bacterial population, treated using inhibitors and debridement [26]. There the focus was upon optimising inhibitor properties to improve treatment. Here we consider how to optimally combine antibiotic, inhibitor and debridement therapies so as to eliminate a mixed susceptible-resistant population of bacteria.

Antibiotics are applied systemically and may be administered either in discrete doses (e.g. administered orally as tablets) or continuously (e.g. administered intravenously via a drip). In the continuous case the antibiotic concentration is held at a constant value such that *A* ≡ *A*_*init*_. Hellinger *et al*. [59] have shown that meropenem dosages as high as 6 g day^−1^ can be used in humans without increasing the frequency of adverse effects, a result which has been confirmed by other groups [60, 61]. Furthermore, Roberts *et al*. [60] found that continuous dosing of meropenem at 3 g day^−1^ in humans resulted in subcutaneous tissue concentrations of 4 *μ*g cm^−3^. Therefore, since the daily dosage could be up to twice this value, we can infer (assuming a linear scaling) that subcutaneous tissue (and hence burn wound) concentrations up to 8 *μ*g cm^−3^ are achievable. In the discrete dosing case, antibiotic degrades and is cleared from the body following each dosing event. We assume that discrete doses may not exceed tissue concentrations of 8 *μ*g cm^−3^, consistent with the continuous case. We take the dosing frequency to be once a day, at the same times at which inhibitors are applied (see below), thus ensuring that our treatment regimens are feasible to implement clinically.

Inhibitors are applied topically to the wound. There is no hard limit on how frequently inhibitors may be applied; however, twice daily is a reasonable upper limit (that is, at 0, 12, 24, 36, … hr), fixing the frequency at daily dosing in the present study for simplicity. We take the dose used by Huebinger *et al*. [18] in their experiments, that is 3×10^8^ inhibitors (which, when added to the exudate, corresponds to a concentration of 6.12×10^7^ inhib. cm^−3^), as standard. The total number of inhibitors in the system (free and bound) is conserved in the absence of debridement, except during the first 24 hr (after the necrotic tissue is first excised), when free inhibitors are lost through leakage of the exudate.

Debridement involves the mechanical cleansing of a wound, for example with a cloth. In our model this corresponds to the instantaneous removal of the exudate and with it all of the free bacteria and inhibitors. The exudate is quickly replenished (on the timescale of a few minutes) such that its volume fluctuation can be neglected. Debridement can be administered at most once daily, starting from the first day after the excision is made (that is at 24, 48, 72, … hr). In those cases where debridement and dosing with inhibitors coincides, debridement is performed first, to avoid immediately removing the newly administered inhibitors. Since debridement involves the removal of the scab that forms over the wound, clearance of bacteria and inhibitors is re-established in the first 24 hr after each debridement event.

### Parameter fitting and justification

The parameters in Table 3 were fitted to newly gathered *in vitro* data. Susceptible, PA1004 WT, and resistant, PA1004 Evo10, strains of *P. aeruginosa* were grown both in the absence of antibiotic and in the presence of a range of concentrations of meropenem. Simplified equations, containing only logistic growth and antibiotic killing terms were then fitted to the data using the Matlab routine fminsearch, providing fits for *c*, $E_{max}^{S}$, $E_{max}^{R}$, $A_{50}^{S}$ and $A_{50}^{R}$. See S1 Text for further details.

The parameters in Table 2 come from [26] where they were fitted to *in vivo* data from the rat burn wound model described in [18]. Twelve valid parameter sets were identified, which were grouped into four qualitatively distinct cases (Case A–Case D). Treatment with inhibitors is effective in Cases A and B, worsens an infection in Case C and has little effect in Case D. Each parameter set gave an equally good fit to the data, while insufficient experimental data is currently available to distinguish between them. In the present work we use a single parameter set from each case, Set 2 from Case A, Set 6 from Case B, Set 10 from Case C and Set 12 from Case D. Set 2 was chosen as it is the most biologically realistic parameter set in Case A, Sets 6 and 10 were chosen since they are the most resistant to treatment, allowing us to consider the worst-case-scenario, and Set 12 was chosen since it is the only parameter set in Case D. We note that we used Sets 3 and 8, rather than Sets 6 and 10, in the main text of [26] in Cases B and C respectively.

The combination of the parameters fitted to *in vitro* data in Table 3 with the parameters fitted to *in vivo* data in Table 2 is valid, both since the effects and processes with which each set of parameters are associated are independent from each other, and because the *in vitro* data were gathered using the same bacterial species (*P. aeruginosa*) as the *in vivo* data and using a growth medium which replicates the nutrient levels in a burn wound exudate (see S1 Text).

Each of the parameters in Table 4 were either measured, calculated, derived from the literature or estimated, as indicated in the fourth column. The parameters *ϕ*_*Bac*_, *ϕ*_*I*_, *V*, *A*_*r*_, *h*, *n* and *E*_*total*_ are justified in [26] (where *E*_*total*_ is written as *E*_*init*_), while $I_{F_{init}}$ and *A*_*init*_ are justified above in Treatment types.

We set the conjugation and segregation rates, *λ* and *ρ*, to zero unless otherwise stated. This is because the resistance genes to meropenem in the PA1004 Evo10 strain of *P. aeruginosa* under consideration are chromosomal and hence cannot be transferred by conjugation or lost through segregation, which requires the resistance gene to be carried on a plasmid. While we have the PA1004 Evo10 strain in mind throughout this study, we have included terms for conjugation and segregation in order to make our model sufficiently general to account for other bacterial strains. In S3 Text we perform a sensitivity analysis to investigate the effect of these parameters on the bacterial dynamics, using values informed by the literature as described below.

Hall *et al*. [50] measured the intraspecific conjugation rates of *P. fluorescens* and *P. putida* to be 10^−11±0.2^ cell^−1^hr^−1^ and 10^−14±0.4^ cell^−1^hr^−1^ respectively (these values must be multiplied by *V* = 4.9 cm^3^ to make them dimensionally consistent with our model), which fall within the range of values measured by [51–53], while Simonsen *et al*. [51] have measured conjugation rates as high as 10^−9^ cm^3^ cell^−1^hr^−1^ in *E. coli*.

Smets *et al*. [52] measured a plasmid loss rate of 2.52×10^−4^hr^−1^ which informed the value of 1×10^−4^hr^−1^ used in [50, 53] and falls within the range of values measured by [54].

The antibiotic elimination rate, *δ*_*A*_, has been measured to lie in the range 0.62–1.72 hr^−1^ for meropenem in both humans and pigs [55–57], thus we choose *δ*_*A*_ = 1 hr^−1^ as a typical value.

The factor difference in antibiotic potency against bound bacteria *ω* is assumed to be one (i.e. no difference) by default and in the absence of further information. We perform a sensitivity analysis on *ω* in Sensitivity analysis, varying it within the range *ω* ∈ [0.5, 2].

The initial bacterial burden is taken to be 5×10^6^ CFU (colony-forming units), corresponding to an initial free density (all bacteria are free initially) of 1.02×10^6^ cells cm^−3^, in accordance with the *in vivo* model in [18]. The initial ratio of susceptible to resistant bacteria may vary; however, susceptible bacteria will be in the majority prior to treatment with antibiotic due to the resistance-associated fitness cost. Therefore, we assume that only 2% (2.04×10^4^ cells cm^−3^) of the initial bacterial population exhibits the resistant phenotype, the remaining 98% (1.00×10^6^ cells cm^−3^) being susceptible.

## Results

In each of the results presented below, we consider the behaviour of the model, given by Eqs 1–11, for each of the parameter sets denoted as Cases A–D (see Parameter fitting and justification). Rather than provide plots for each of the dependent variables, we typically plot some combination of the total number of free bacteria, $B_{F}=V\left(B_{F_{S}}+B_{F_{R}}\right)$, the total number of bound bacteria, $B_{B}=A_{r}\left(B_{B_{S}}+B_{B_{R}}\right)$, or the total number of bacteria, *B*_*T*_ = *B*_*F*_ + *B*_*B*_, since these are the quantities of greatest interest. We begin with a steady-state analysis of the system to determine the number of steady-states and their stability properties. Next, simulations of the full time-dependent problem under a range of treatment regimens are discussed. We then consider a series of sensitivity analyses to determine the effect of the size of the inhibitor and antibiotic doses, together with other key parameters, upon the bacterial population dynamics. Lastly, we use our model to predict optimal treatment regimens. We note that a treatment is considered to have eliminated the bacterial burden if the total number of bacteria, *B*_*T*_, is reduced beneath one.

### Steady-state analysis

We begin by considering a steady-state analysis of Eqs 1–10, performed using Maple, to determine the number of steady-states exhibited by the system under various conditions, together with their stability properties. While the system may take a long while to approach steady-state in practice, depending upon the choice of parameters and initial conditions, this analysis is instructive for at least two reasons. Firstly, it allows us to ensure that we are not overlooking any potential stable steady-state solutions in the time-dependent simulations presented below. Secondly, it allows us to make more clear-cut comparisons between different scenarios, looking beyond the transient dynamics resulting from the choice of initial conditions.

We consider four scenarios: untreated, antibiotic treatment only, inhibitor treatment only, and treatment with both antibiotics and inhibitors, comparing Cases A–D in each scenario. We use the maximum continuous concentration (8 *μ*g cm^−3^) for antibiotic treatment and a single standard dose (6.12×10^7^ inhib. cm^−3^) for inhibitor treatment (see Treatment types). We set the clearance terms to zero (${\tilde{\psi }}_{Bac}=0$ hr^−1^ and ${\tilde{\psi }}_{I}=0$ hr^−1^) since fluid only leaks from the wound in the first 24 hr. Further, we neglect conjugation and segregation (*λ* = 0 cm^3^cell^−1^hr^−1^ and *ρ* = 0 hr^−1^), and assume that there is no difference in the potency of antibiotics against bound bacteria as compared with free bacteria (*ω* = 1) (see Parameter fitting and justification). We also remove the Heaviside step functions preventing the logistic growth of bacteria when their population size goes beneath one ($H(VB_{F_{S}}-1)$, $H(VB_{F_{R}}-1)$, $H(A_{r}B_{B_{S}}-1)$ and $H(A_{r}B_{B_{R}}-1)$) since these are only required in dynamic simulations to prevent biologically unrealistic regrowth when bacteria have been eliminated. Lastly, where antibiotics are applied, we assume a constant dose, since with a discrete dose the antibiotic concentration is zero at steady-state, being identical to the equivalent scenario without antibiotic treatment. Following these simplifications, the governing equations reduce to Eqs A–E in S2 Text. All remaining parameter values are as given in Tables 2–4.

**Fig 2:**
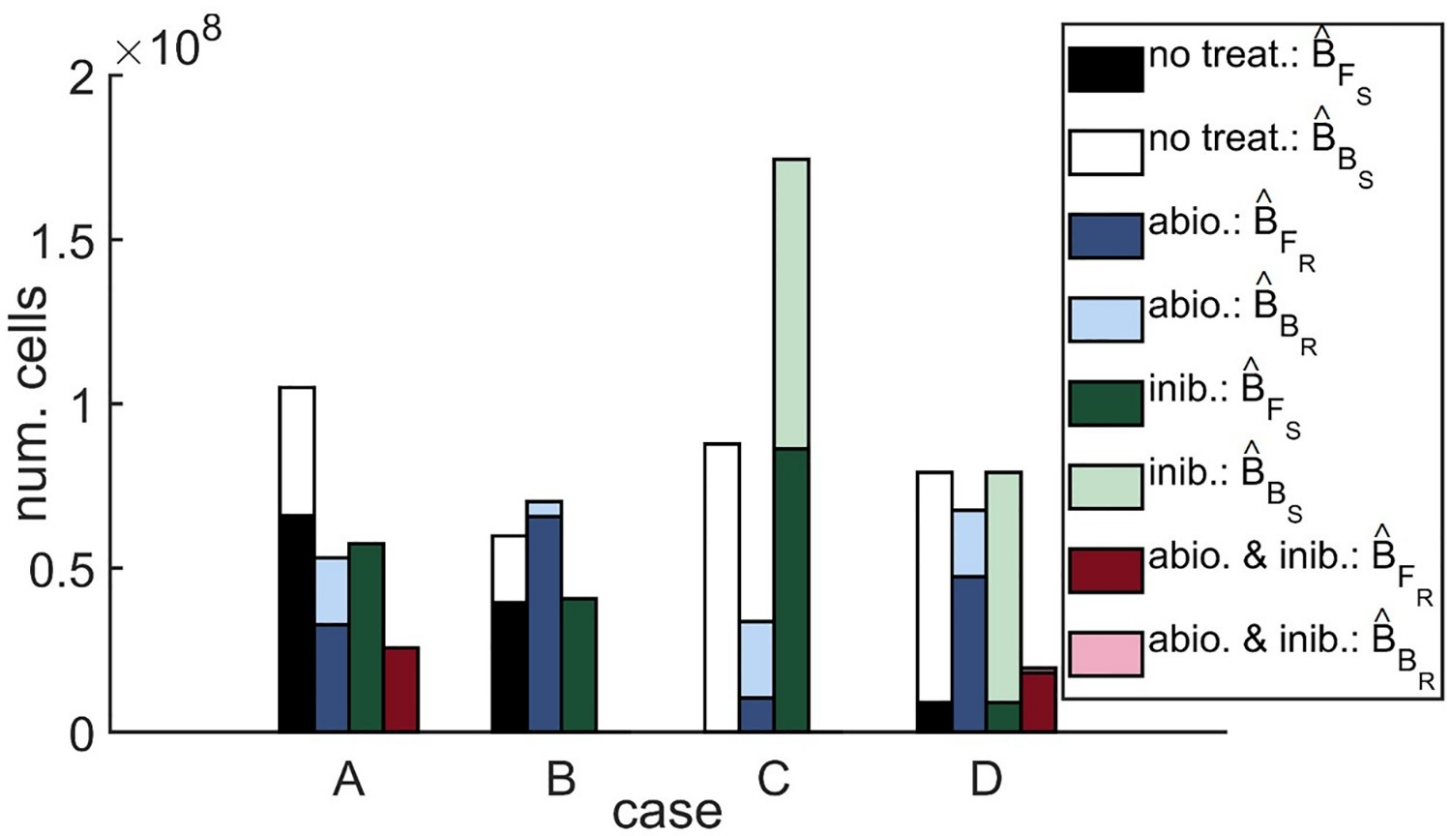
Steady-state solutions. Four stacked bars are plotted for each case: the first bar shows the number of free and bound susceptible bacteria, ${\hat{B}}_{F_{S}}=VB_{F_{S}}$ and ${\hat{B}}_{B_{S}}=A_{r}B_{B_{S}}$, at steady-state in the untreated scenario; the second bar shows the number of free and bound resistant bacteria, ${\hat{B}}_{F_{R}}=VB_{F_{R}}$ and ${\hat{B}}_{B_{R}}=A_{r}B_{B_{R}}$, at steady-state in the antibiotic only scenario; the third bar shows the number of free and bound susceptible bacteria at steady-state in the inhibitor only scenario, and the fourth bar shows the number of free and bound resistant bacteria at steady-state in the antibiotic and inhibitor scenario. There are no resistant bacteria at steady-state in the absence of antibiotic, nor are there any susceptible bacteria at steady-state in the presence of antibiotic. Thus, the combined height of the bars in each stack gives the total number of bacteria, *B*_*T*_. Treatment with antibiotics alone reduces the total bacterial burden in Cases A, C and D, and increases it in Case B. Treatment with inhibitors alone reduces the total bacterial burden in Cases A and B, increases it in Case C and has a negligible effect in Case D. Treatment with both antibiotics and inhibitors eliminates the bacterial burden in Cases B and C, and reduces it more than either treatment in isolation in Cases A and D. Steady-state solutions to Eqs 1–10 were calculated using Maple, neglecting the Heaviside step functions which prevent the logistic growth of bacteria when their population size goes beneath one. Parameter values: ${\tilde{\psi }}_{Bac}=0$ hr^−1^, ${\tilde{\psi }}_{I}=0$ hr^−1^, *λ* = 0 cm^3^cell^−1^hr^−1^, *ρ* = 0 hr^−1^, *ω* = 1, *A* = 0 or 8 *μ*g cm^−3^, and $I_{F_{init}}=0$ or 6.12×10^7^ inhib. cm^−3^. See Tables 2–4 for the remaining parameter values.

The results of the steady-state analysis are described in detail in S2 Text and summarised here, in Table 5 and in Fig 2. In all cases except Case A under the inhibitor only treatment the system is monostable, the number of steady-states (stable plus unstable) varying between one and three depending upon the treatment scenario and the parameter set. In the absence of antibiotics, resistant bacteria go extinct at the stable steady-state, while free and bound susceptible bacteria survive. The situation is reversed in the presence of antibiotics, with susceptible bacteria going extinct at the stable steady-state, while free and bound resistant bacteria survive. There are three exceptions to this rule. The first two are for the scenario in which treatment with both antibiotics and inhibitors is applied, in Cases B and C, for which all bacteria go extinct at the stable steady-state. The third is Case A under the inhibitor only treatment (noted above), for which there exist no isolated stable steady-states. Instead, there exists a region of non-isolated steady-states [64], in which susceptible and resistant bacteria may coexist, including the extremes (unstable steady-states) at which only one of these subtypes survives. As such, the state to which the system settles depends upon the initial conditions. For simplicity of exposition, we plot the unstable steady-state solution in which only susceptible bacteria survive in Fig 2, this being the typical state in the absence of antibiotics under most parameter sets.

**Table 5:** Steady-states and their stability properties.

Steady-state	No Treatment				Abio. Only				Inhib. Only				Abio. and Inhib.			
	A	B	C	D	A	B	C	D	A	B	C	D	A	B	C	D
No Bacteria	U	U	U	U	U	U	U	U	U	U	U	U	U	S	S	U
Susceptible Only	S	S	S*	S	—	—	—	U	U	S	S	S	—	—	—	U
Resistant Only	U	U	U*	U	S	S	S	S	U	U	U	U	S	—	—	S

The steady-states and their stability properties are summarised for four treatment scenarios (no treatment, antibiotic only, inhibitors only, and antibiotics and inhibitors), for Cases A–D. There are three types of steady-state: those in which bacteria are absent, those in which only susceptible bacteria survive and those in which only resistant bacteria survive. S: stable steady-state, U: unstable steady-state and ‘—’: no steady-state. All steady-states are pure nodes except for those marked with a star which are mixed nodes/spirals, where the eigenvalues include a single pair of complex conjugates.

Treatment with antibiotics alone reduces the total number of bacteria, *B*_*T*_, in Cases A, C and D; however, it slightly increases the bacterial burden in Case B (this counter-intuitive result is discussed below in Sensitivity analysis). As was the case in [26], treatment with inhibitors reduces the total number of bacteria in Cases A and B, increases the bacterial burden in Case C and has little effect in Case D (see Sensitivity analysis and [26] for discussion). Treatment with antibiotics and inhibitors in combination is more effective than treatment with either therapy in isolation, eliminating the bacterial burden in Cases B and C and greatly reducing it in Cases A and D. It is evident from these results that antibiotics and inhibitors are predicted to work together in a synergistic manner, as opposed to an additive one, their combined effect reducing the total bacterial burden by a greater quantity in Cases B–D than the sum of the reductions when applied in isolation, and by a smaller quantity in Case A.

### Dynamic simulations

**Fig 3:**
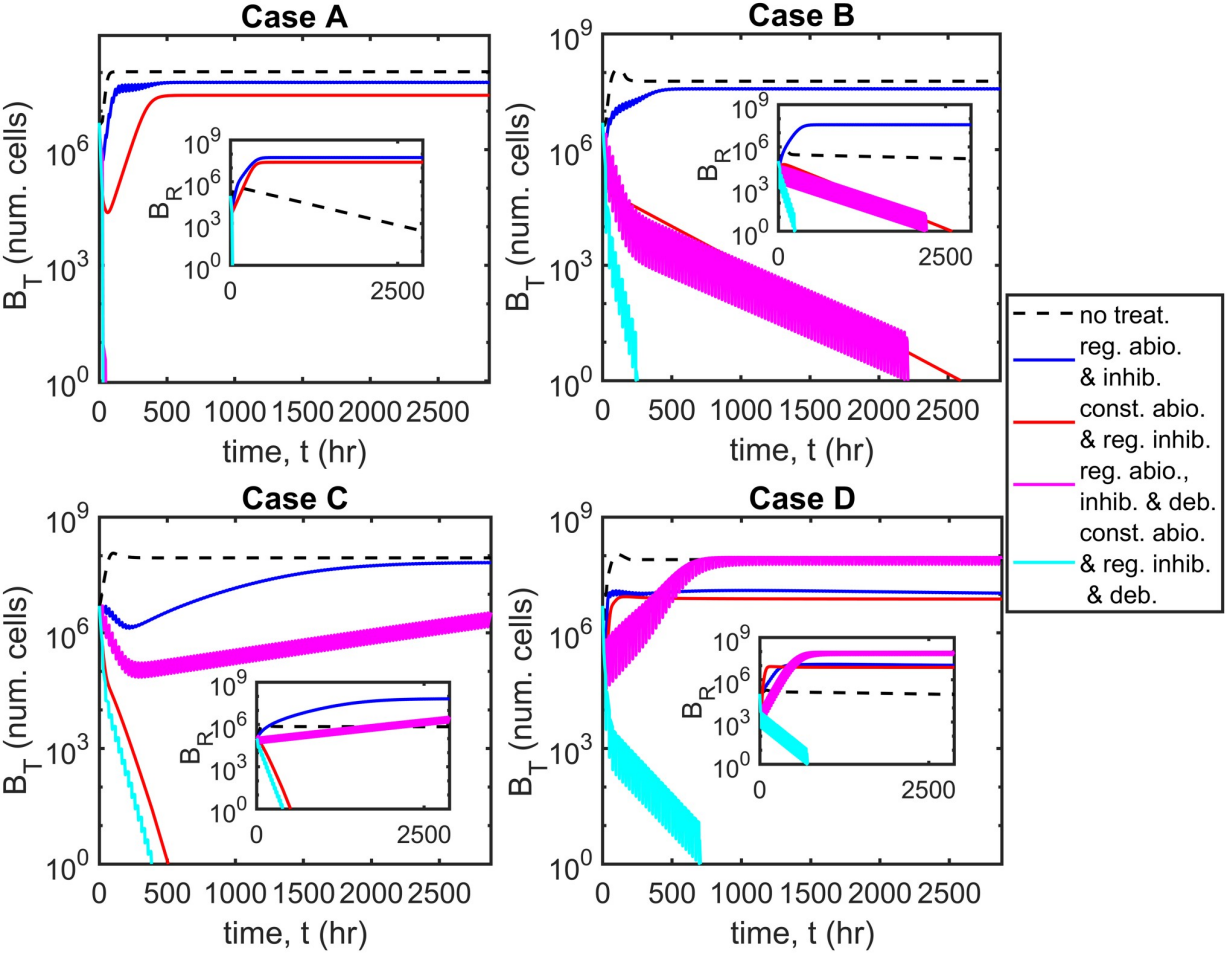
Dynamic simulations. The total number of bacteria, *B*_*T*_, is shown over time for the untreated scenario and for four treatment strategies: regular antibiotic and inhibitor dosing with and without regular debridement, and constant antibiotic concentration with regular inhibitor dosing, with and without regular debridement. Note the log_10_ scale on the *y*-axis. Inset graphs show the number of resistant bacteria $B_{R}=VB_{F_{R}}+A_{r}B_{B_{R}}$. Treatment with constant antibiotics together with regular inhibitor dosing and debridement is most effective, eliminating the bacterial population in all cases (A–D), while regular antibiotic and inhibitor dosing without debridement is least effective, failing to eliminate the bacterial burden in all cases. The remaining two treatment strategies are intermediate in their efficacy, eliminating the bacterial population in some, but not all cases. Eqs 1–11 were solved using ode15s. Parameter values: *λ* = 0 cm^3^cell^−1^hr^−1^, *ρ* = 0 hr^−1^ and *ω* = 1. Antibiotics doses: 8 *μ*g cm^−3^, inhibitor doses: 6.12×10^7^ inhib. cm^−3^, constant antibiotic scenarios: *A* = 8 *μ*g cm^−3^. See Tables 2–4 for the remaining parameter values.

Having examined the behaviour of the system at steady-state, we consider the bacterial population dynamics over time in response to treatment. We use the Matlab routine ode15s, a variable-step, variable-order solver based upon numerical differentiation formulas, to solve the time-dependent problem (Eqs 1–11) both here and throughout the paper. The untreated scenario is compared with four treatment scenarios: regular antibiotic and inhibitor dosing with and without regular debridement, and constant antibiotic concentration with regular inhibitor dosing, with and without regular debridement (see Fig 3), for Cases A–D. We note that while only the total number of bacteria, *B*_*T*_, is plotted for clarity, the simulations include susceptible/resistant and free/bound bacteria. Regular antibiotic/inhibitor/debridement treatments are performed every 24 hr, antibiotic/inhibitor dosing occurring for the first time at *t* = 0 hr and debridement being performed for the first time at *t* = 24 hr. Antibiotic doses of 8 *μ*g cm^−3^ and standard inhibitor doses of 6.12×10^7^ inhib. cm^−3^ are used in all cases, while each debridement event results in the removal of all free bacteria and inhibitors. The antibiotic concentration is held fixed at *A* = 8 *μ*g cm^−3^ in the constant antibiotic scenarios (see Treatment types for more details).

Constant antibiotic concentration with regular inhibitor dosing and debridement is the most effective treatment, eliminating the bacterial burden in all cases and doing so more rapidly than the other strategies. Constant antibiotic concentration with regular inhibitor dosing and no debridement eliminates the bacterial population in Cases B and C, but has a more modest effect in Cases A and D. Regular antibiotic and inhibitor dosing with debridement eliminates all bacteria in Cases A and B, but is ineffective in Cases C and D. Lastly, regular antibiotic and inhibitor dosing without debridement is the least effective strategy, having little effect in Cases A–D. We note that while constant antibiotic concentration with regular inhibitor dosing and debridement reduces the number of antibiotic resistant bacteria, $B_{R}=VB_{F_{R}}+A_{r}B_{B_{R}}$, in all cases (see inset graphs, Fig 3), the remaining strategies increase *B*_*R*_ above untreated levels in some cases; indeed, regular antibiotic and inhibitor dosing without debridement does so in all cases.

A comparison between regular inhibitor and debridement treatment, which is the most effective therapy in the absence of antibiotics (see [26]), and treatment which combines regular inhibitor and debridement therapy with a constant antibiotic dose, shows that the combined therapy is significantly more effective (see Fig. A in S3 Text). Inhibitors and debridement alone eliminate the bacterial burden in Case A only, whereas, when combined with antibiotics, the bacterial burden is eliminated in all four cases (A–D).

### Sensitivity analysis

In the results that follow we consider the effect of varying the antibiotic and inhibitor doses, and other key parameters, upon the bacterial population dynamics and their steady-state values. In the cases where time-dependent simulations are employed, the solutions are shown at 4 weeks (672 hr), with solutions at 1 week (168 hr) and 1 year (365 days = 8760 hr) provided in S3 Text. Results are given at 1 week since ideally we would like to clear an infection within this time, while results are shown at 4 weeks and 1 year to demonstrate the dynamics of more persistent infections and since the sensitivity of the system to changes in parameter values varies over time.

#### Antibiotic and inhibitor doses

**Fig 4:**
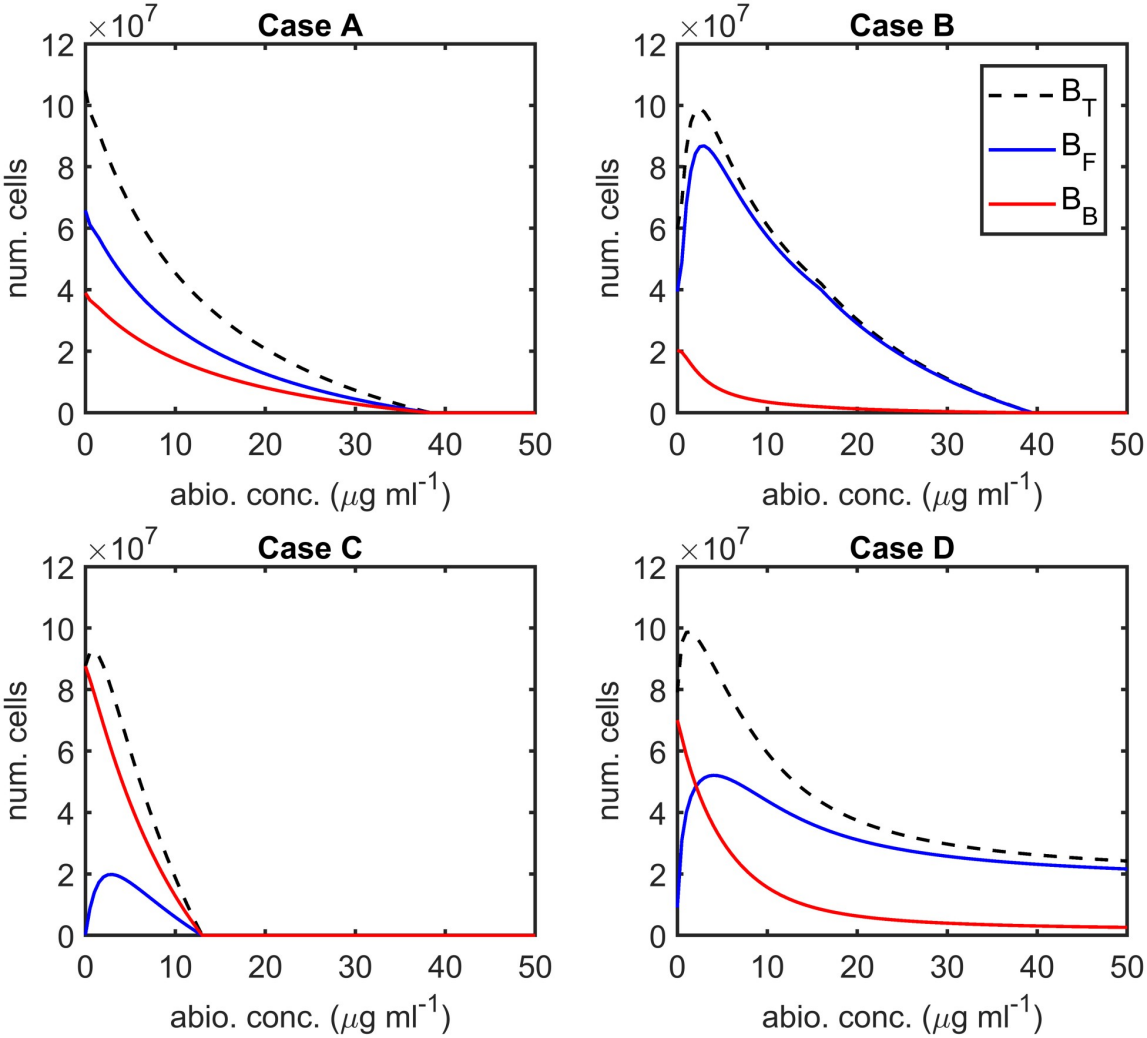
Steady-state sensitivity analysis for antibiotic concentration. The number of bound bacteria, *B*_*B*_, is a monotone decreasing function of the antibiotic concentration, *A*, in all cases, as are the number of free bacteria, *B*_*F*_, and the total number of bacteria, *B*_*T*_, in Case A. In Cases B–D the free and total bacterial burdens increase initially, before decreasing with increasing antibiotic concentration. (Note that, except near *A* = 0 *μ*g ml^−1^, all bacteria are resistant at steady-state in the presence of antibiotic, see Steady-state analysis). Steady-state solutions to Eqs 1–10 were calculated using fsolve, neglecting the Heaviside step functions which prevent the logistic growth of bacteria when their population size goes beneath one. Parameter values: ${\tilde{\psi }}_{Bac}=0$ hr^−1^, ${\tilde{\psi }}_{I}=0$ hr^−1^, *λ* = 0 cm^3^cell^−1^hr^−1^, *ρ* = 0 hr^−1^, *ω* = 1 and $I_{F_{init}}=0$ inhib. cm^−3^. See Tables 2–4 for the remaining parameter values.

Fig 4 shows the effect of varying the antibiotic concentration, *A*, upon the total number of bacteria, *B*_*T*_, the number of free bacteria, *B*_*F*_, and the number of bound bacteria, *B*_*B*_, at steady-state, in the absence of inhibitors (we note that, except in a very narrow region around *A* = 0 *μ*g ml^−1^, all bacteria are resistant at steady-state in the presence of antibiotic, see Steady-state analysis). Eqs 1–10 were solved using the Matlab routine fsolve, employing the Trust-Region-Dogleg algorithm, in the same form and with the same parameter values as in Steady-state analysis, but with *A* ∈ [0, 50] *μ*g ml^−1^. This range of antibiotic values goes well above the maximum concentration discussed in Treatment types. We use a wider range here in order to elucidate the theoretical effect of increased antibiotic concentrations in isolation.

It can be seen that *B*_*B*_ is a monotone decreasing function of *A* in all cases (A–D), as are *B*_*F*_ and *B*_*T*_ in Case A. However, in Cases B–D, we have the counter-intuitive result that *B*_*F*_ increases initially as *A* is increased above zero, before reaching a maximum and decreasing, causing a similar behaviour in *B*_*T*_. The increase in *B*_*F*_, and hence in *B*_*T*_, is caused by an increase in the logistic growth rate of bound bacteria, occurring as a result of a decrease in the number of bound bacteria, the majority of the daughter cells from bound bacteria entering the exudate since *η*_*max*_ ≪ 1 in all cases. Given the quadratic dependence of the bound logistic growth rate upon the number of bound bacteria, a change to the system resulting in a reduction in the number of bound bacteria has the potential to increase the bound growth rate provided the original bound bacterial population is greater than half the bound carrying capacity, *K*_*B*_/2 (see [26] for more details). Since the increase in the number of free bacteria outweighs the decrease in bound bacteria, this also increases the total bacterial burden. All bacteria are eliminated for sufficiently high antibiotic concentrations in Case A–C, while the antibiotic killing effect saturates in Case D such that *B*_*T*_ does not drop far below 2 × 10^7^ as the antibiotic concentration is increased beyond 50 *μ*g ml^−1^.

**Fig 5:**
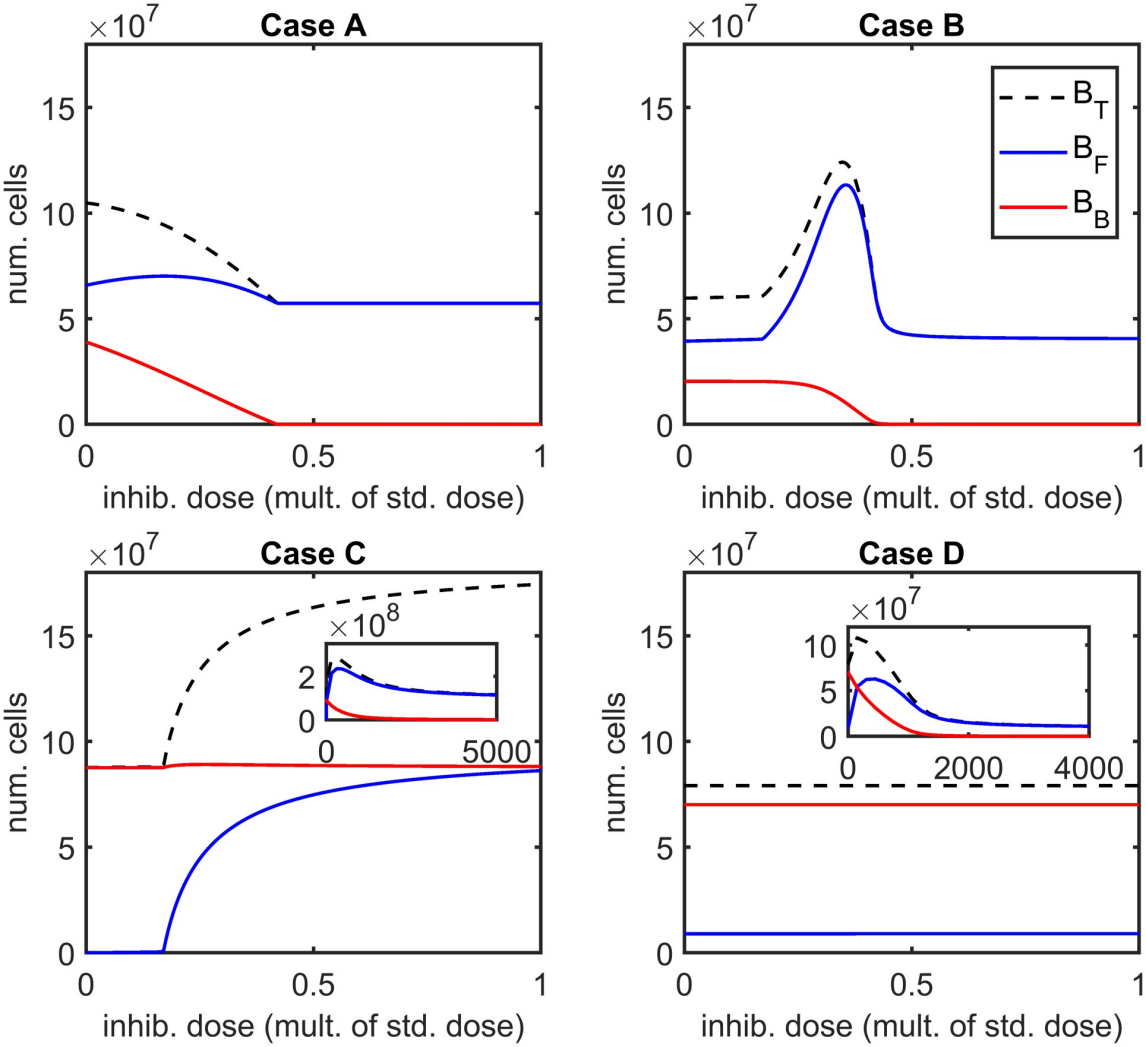
Steady-state sensitivity analysis for inhibitor doses. Note that inhibitor treatments are plotted as multiples of the standard dose (6.12×10^7^ inhib. cm^−3^). In Cases A and B the number of bound bacteria, *B*_*B*_, is a monotone decreasing function of the inhibitor dose, $I_{F_{init}}$, while the number of free bacteria, *B*_*F*_, increases initially, before decreasing with increasing inhibitor dose. The total number of bacteria, *B*_*T*_, is a monotone decreasing function of the inhibitor dose in Case A, while it increases initially, before decreasing with increasing inhibitor dose, in Case B. In Case C both free and bound bacterial numbers, and hence the total number of bacteria, are increasing functions of the inhibitor dose within the range $I_{F_{init}}\in \left[0,6.12\times 10^{7}\right]$ inhib. cm^−3^, while in Case D inhibitors have little effect on the steady-state bacterial numbers within this range. The insets in the lower panels show the steady-state behaviour for higher inhibitor doses. Eqs 1–11 were solved using ode15s, allowing the system to evolve until it reached steady-state and neglecting the Heaviside step functions which prevent the logistic growth of bacteria when their population size goes beneath one. Parameter values: ${\tilde{\psi }}_{Bac}=0$ hr^−1^, ${\tilde{\psi }}_{I}=0$ hr^−1^, *λ* = 0 cm^3^cell^−1^hr^−1^, *ρ* = 0 hr^−1^, *ω* = 1 and *A* = 0 *μ*g cm^−3^. See Tables 2–4 for the remaining parameter values.

Fig 5 shows the effect of varying the (single) inhibitor dose, $I_{F_{init}}$, upon the total number of bacteria, the number of free bacteria and the number of bound bacteria at steady-state, in the absence of antibiotic (we note that all bacteria are susceptible at steady-state in the absence of antibiotic, see Steady-state analysis). As discussed in Steady-state analysis there are inhibitor concentrations at which the system does not possess an isolated steady-state in Case A; therefore, in this case, we plot the steady-state for which only susceptible bacteria survive across all values of $I_{F_{init}}$ for consistency. The Matlab routine ode15s was used to solve the time-dependent problem (Eqs 1–11), allowing the system to evolve until it reached steady-state (fsolve struggles to find the steady-state solution when inhibitors are included due to the difficulty in choosing an initial guess that will converge to the desired steady-state). Aside from using the time-dependent form of the equations, the same assumptions were made as in Steady-state analysis, but with $I_{F_{init}}\in \left[0,6.12\times 10^{7}\right]$ inhib. cm^−3^, the maximum dose being the standard dose as discussed in Treatment types.

It can be seen that *B*_*B*_ is a monotone decreasing function of $I_{F_{init}}$ in Cases A and B, while the number of free bacteria increases initially as $I_{F_{init}}$ is increased above zero, before reaching a maximum and decreasing. This rather surprising increase in the number of free bacteria results from the same phenomenon as that described above for antibiotic treatment, whereby a decrease in the number of bound bacteria results in an increase in their growth rate and hence in the rate of their contribution of daughter cells to the exudate. In Case B this causes the total number of bacteria to increase for intermediate vales of $I_{F_{init}}$, while in Case A the reduction in bound bacteria is greater than the increase in free bacteria such that the total number of bacteria is a monotone decreasing function of $I_{F_{init}}$. In both Cases A and B, further increases in $I_{F_{init}}$ above about half the standard dose have relatively little effect on the steady-state bacterial population.

Case C gives the highly counter-intuitive prediction that the addition of inhibitors will increase both the number of free and bound bacteria, and, therefore, the total bacterial burden, within the range $I_{F_{init}}\in \left[0,6.12\times 10^{7}\right]$ inhib. cm^−3^. This cannot be due to the phenomenon described above since *B*_*B*_ is close to its carrying capacity, *K*_*B*_, in the absence of inhibitors and comes to slightly exceed it as $I_{F_{init}}$ is increased. Rather, the addition of inhibitors greatly decreases the free binding site density, *E*, such that the number of free bacteria can greatly increase before the bacterial binding terms in Eqs 1–2, which act as a sink on free bacteria, achieve a similar magnitude to that in the inhibitor free case. Indeed, since the increase in free bacterial numbers results in an increase in the growth rate of free bacteria, the magnitude of the bacterial binding terms can exceed that in the untreated case before the growth and binding terms balance in Eqs 1–2. Consequently, the bacterial binding terms, which act as a source in Eqs 3–4, make a greater contribution to the number of bound bacteria, increasing the bound bacterial burden beyond that in the untreated case. Lastly, in Case D, the addition of inhibitors has a negligible effect within the range $I_{F_{init}}\in \left[0,6.12\times 10^{7}\right]$ inhib. cm^−3^. The inset graphs for Cases C and D show that at higher inhibitor doses *B*_*B*_ falls, while *B*_*F*_ and *B*_*T*_ rise and fall. We note that this behaviour only occurs for unrealistically high doses, thousands of times higher than the standard dose.

**Fig 6:**
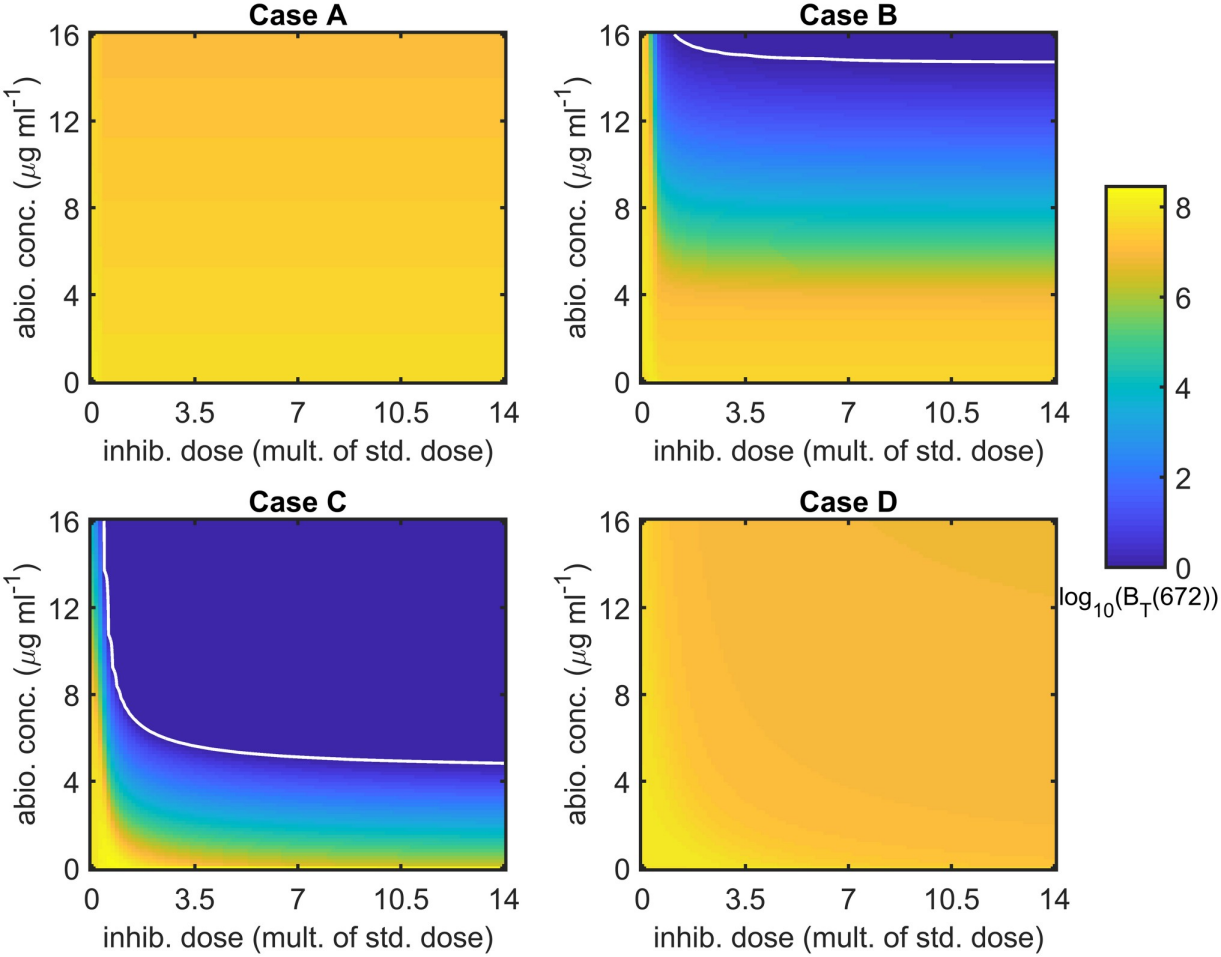
Sensitivity analysis for antibiotic concentration and inhibitor dose—4 weeks. The log_10_ of the total number of bacteria at 4 weeks (672 hr), *B*_*T*_(672), is plotted for a range of antibiotic concentrations and inhibitor doses. Note that inhibitor treatments are plotted as multiples of the standard dose (6.12×10^7^ inhib. cm^−3^) and that values of *B*_*T*_(672) < 1 are plotted as *B*_*T*_(672) = 1 to maximise visual clarity. The white curves are the contours along which *B*_*T*_(672) = 1; hence, *B*_*T*_(672) < 1 above-right of these contours. The effect of treatment is relatively minor in Cases A and D; however, the bacterial burden may be eliminated for sufficiently high antibiotic concentrations and inhibitor doses in Cases B and C. Eqs 1–11 were solved using ode15s and with a constant antibiotic concentration. Parameter values: ${\tilde{\psi }}_{Bac}=0$ hr^−1^, ${\tilde{\psi }}_{I}=0$ hr^−1^, *λ* = 0 cm^3^cell^−1^hr^−1^, *ρ* = 0 hr^−1^ and *ω* = 1. See Tables 2–4 for the remaining parameter values.

Fig 6 shows the effect of varying the antibiotic concentration and inhibitor dose upon the total number of bacteria at *t* = 672 hr (4 weeks). The full model (Eqs 1–11) was solved with a constant antibiotic concentration and with ${\tilde{\psi }}_{Bac}=0$ hr^−1^, ${\tilde{\psi }}_{I}=0$ hr^−1^, *λ* = 0 cm^3^cell^−1^hr^−1^, *ρ* = 0 hr^−1^ and *ω* = 1, for *A* ∈ [0, 16] *μ*g ml^−1^ and $I_{F_{init}}\in \left[0,8.57\times 10^{8}\right]$ inhib. cm^−3^, where the antibiotic upper bound is twice the maximum concentration that could be applied constantly, while the inhibitor upper bound is fourteen times the standard dose. The white curves are the contours along which *B*_*T*_(672) = 1, separating regions in which bacteria survive from those in which they are eliminated. Treatment has a relatively minor effect in Cases A and D; however, the bacterial burden can be eliminated in Cases B and C, provided the antibiotic concentration and inhibitor dose are high enough, corresponding to the region above-right of the white contours. Note that the use of a modest dose of inhibitors is sufficient to dramatically reduce the antibiotic concentration required to eliminate the bacterial burden in these cases. The equivalent results at 1 week and 1 year are given in Figs B and C in S3 Text. Treatment is less effective after 1 week than after 4 weeks in Cases B and C, but more effective in Cases A and D, while treatment is more effective in Cases B and C after 1 year than after 4 weeks and there is little change in Cases A and D over this period.

**Fig 7:**
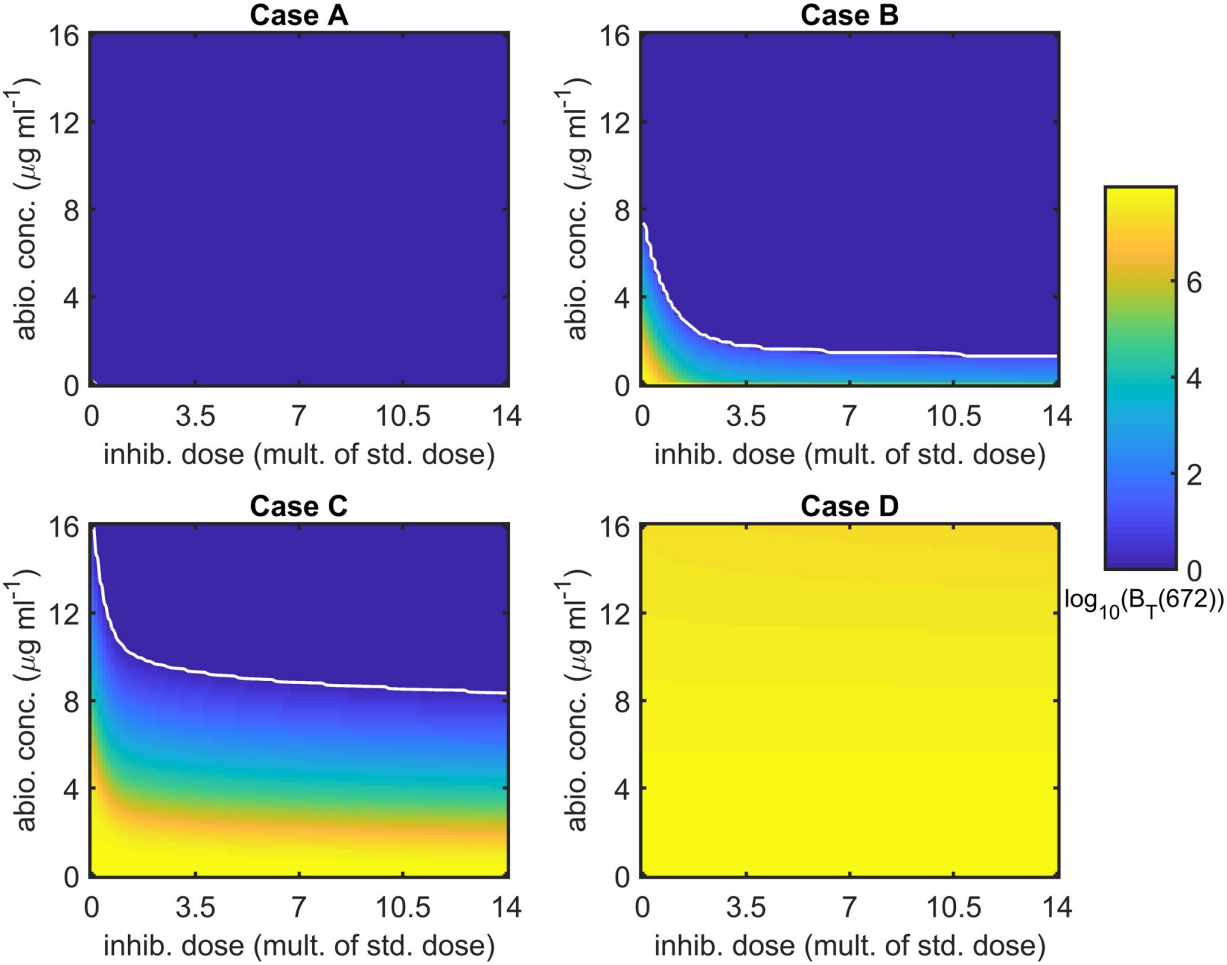
Sensitivity analysis for antibiotic concentration and inhibitor dose with debridement—4 weeks. The log_10_ of the total number of bacteria at 4 weeks (672 hr), *B*_*T*_(672), is plotted for a range of antibiotic concentrations and inhibitor doses. Note that inhibitor treatments are plotted as multiples of the standard dose (6.12×10^7^ inhib. cm^−3^) and that values of *B*_*T*_(672) < 1 are plotted as *B*_*T*_(672) = 1 to maximise visual clarity. The white curves are the contours along which *B*_*T*_(672) = 1; hence, *B*_*T*_(672) < 1 above-right of these contours. All bacteria are eliminated for all antibiotic concentrations and inhibitor doses tested in Case A, except where they are both absent. The bacterial burden can also be eliminated in Cases B and C for sufficiently high antibiotic concentrations and inhibitor doses. Treatment has relatively little effect in Case D, reducing the bacterial population by no more than a factor of five. Eqs 1–11 were solved using ode15s and with a constant antibiotic concentration. Debridement takes place at the start of each day, occurring for the first time at *t* = 24 hr, effecting the removal of all free bacteria and inhibitors. Parameter values: *λ* = 0 cm^3^cell^−1^hr^−1^, *ρ* = 0 hr^−1^ and *ω* = 1. See Tables 2–4 for the remaining parameter values.

Fig 7 shows the effect of varying the antibiotic concentration and inhibitor dose upon the total number of bacteria at *t* = 672 hr (4 weeks) when daily debridement is included. The model details are the same as for Fig 6, except for the inclusion of debridement and the clearance of free bacteria and inhibitors (${\tilde{\psi }}_{Bac}>0$ and ${\tilde{\psi }}_{I}>0$). Treatment is more effective in Cases A and B than in the antibiotic and inhibitor only scenario (Fig 6) and less effective in Cases C and D. The equivalent results at 1 week and 1 year are given in Figs D and E in S3 Text. Treatment is less effective after 1 week than after 4 weeks in Cases A–C, but more effective in Case D. Comparing the results at 4 weeks with those at 1 year, bacteria are always eliminated in Case A at both times, while there is little change in Case D over this period. In Cases B and C, bacteria are eliminated for a larger range of inhibitor doses and antibiotic concentrations at 1 year; however, in those regions of parameter space where bacteria are not eliminated, *B*_*T*_ increases over this period.

#### Conjugation and segregation

The system is insensitive to variation in the rates of conjugation and segregation within realistic ranges. The results are presented and discussed in S3 Text and Figs F–K therein. For this reason and the reasons stated in Parameter fitting and justification, we will continue to neglect conjugation and segregation in the remainder of this paper.

#### Factor difference in antibiotic potency against bound bacteria compared with free bacteria

**Fig 8:**
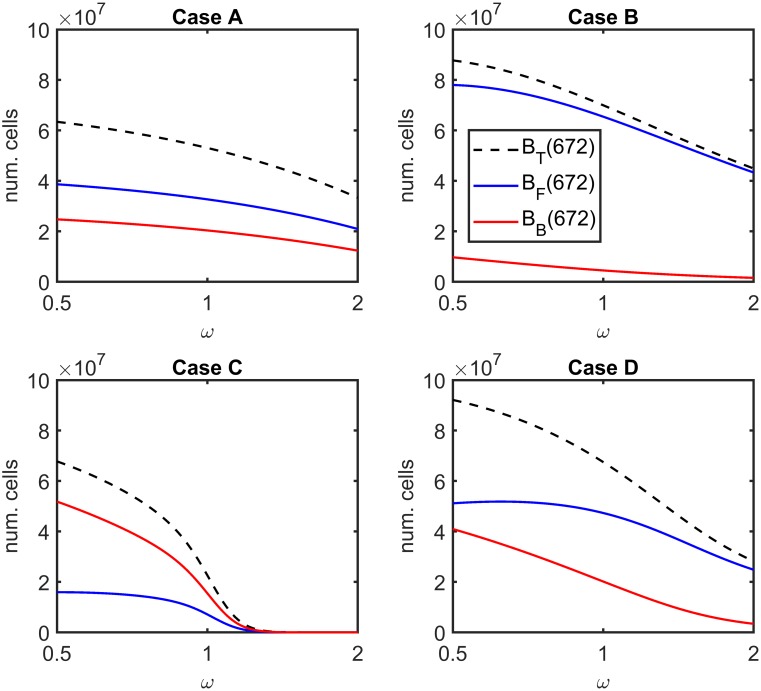
Sensitivity analysis for the factor difference in antibiotic potency against bound bacteria compared with free bacteria—4 weeks. The total number of bacteria and the numbers of free and bound bacteria at 4 weeks (672 hr), *B*_*T*_(672), *B*_*F*_(672) and *B*_*B*_(672) respectively, are plotted for a range of values of the potency factor, *ω*. Note the log_2_ scale on the *x*-axis. In all cases both *B*_*T*_(672) and *B*_*B*_(672) decrease monotonically with increasing *ω*, while *B*_*F*_(672) is monotone decreasing in Cases A–C and increases before decreasing in Case D. The effect is particularly pronounced in Case C, where the bacterial burden is almost eliminated (*B*_*T*_ = *O*(10)) as *ω* approaches 2. Eqs 1–11 were solved using ode15s, with a constant antibiotic dose and without inhibitors. Parameter values: *A* = 8 *μ*g ml^−1^, ${\tilde{\psi }}_{Bac}=0$ hr^−1^, ${\tilde{\psi }}_{I}=0$ hr^−1^, *λ* = 0 cm^3^cell^−1^hr^−1^ and *ρ* = 0 hr^−1^. See Tables 2–4 for the remaining parameter values.

We have assumed until now that antibiotics are equally potent against bound and free bacteria, that is *ω* = 1. Fig 8 shows the effect of varying *ω* upon the total number of bacteria, *B*_*T*_, the number of free bacteria, *B*_*F*_, and the number of bound bacteria, *B*_*B*_, at *t* = 672 hr (4 weeks) (we note that the majority of bacteria are resistant in all cases, such that a plot of the susceptible bacterial population size would almost always lie along the *x*-axis at this scale). The full model (Eqs 1–11) was solved with a constant antibiotic dose of *A* = 8 *μ*g ml^−1^ and without inhibitors, where ${\tilde{\psi }}_{Bac}=0$ hr^−1^, ${\tilde{\psi }}_{I}=0$ hr^−1^, *λ* = 0 cm^3^cell^−1^hr^−1^ and *ρ* = 0 hr^−1^. The factor difference in antibiotic potency was varied in the range *ω* ∈ [0.5, 2]. Both *B*_*B*_ and *B*_*T*_ are monotone decreasing functions of *ω* in all cases within the range examined, while *B*_*F*_ is monotone decreasing in Cases A–C and increases initially, before decreasing, in Case D. The system is sensitive in all cases, and particularly in Case C, for which *B*_*F*_, *B*_*B*_ and *B*_*T*_ are *O*(10) when *ω* = 2. Figs L and M in S3 Text show the equivalent results at 1 week and 1 year. The behaviour is similar to that at 4 weeks, with *B*_*T*_, *B*_*F*_ and *B*_*B*_ monotone decreasing in most cases.

### Optimising treatment

Informed by the preceding sensitivity analyses, we used our mathematical model to predict the optimum treatment regimens in Cases A–D under certain constraints.

Two sets of initial conditions were considered, each consisting of a mixture of susceptible and antibiotic resistant bacteria. The first set is the standard initial conditions given in Table 4, which corresponds to a new infection in which bacteria have not yet had time to bind to host cells. The second set corresponds to an established infection. Here we chose the initial conditions to be the untreated steady-states for each parameter set (Cases A–D), in which all surviving bacteria are susceptible, modified so that 2% of the free and bound bacteria are resistant.

We chose to optimise the treatment over the period of a week—this being a standard period over which to treat a bacterial infection and also reducing the number of regimens over which to search compared with longer periods—exploring combination therapies including antibiotics, inhibitors and debridement. We assume continuous dosing with antibiotics, fixing the concentration at its maximum value of *A* = 8 *μ*g ml^−1^ since this was found to have the greatest effect against bound bacteria (see Fig 4), while free bacteria can be removed using debridement. Debridement may be applied at the beginning of days 2–7, but not at the start of the first day (see Treatment types), giving 2^6^ = 64 possible treatment regimens. Lastly, we assume that inhibitors may be applied in multiples of the standard dose (6.12×10^7^ inhib. cm^−3^), using exactly seven standard doses worth of inhibitors over the week (4.28×10^8^ inhib. cm^−3^), and that inhibitors may only be applied at *t* = 0 hr and immediately following a debridement event. This brings the total number of possible treatment regimens to 14,407. We note that in preliminary work we used a genetic algorithm approach to investigate optimum solutions; however, there is no guarantee of identifying the global optimum via this method. Rather, by accounting for the clinical constraints on the treatment regimen (as described above), we sample the complete space of possible treatment regimens, enabling us to identify the global optimum, subject to these constraints. While the clinical constraints imposed on our optimisation limit the options to a discrete set of points in decision space, we note that the theoretical range of treatment options lies on a continuum (e.g. the timing and concentration of inhibitor doses).

Four separate optimisations were performed for each of Cases A–D and for each set of initial conditions, each using a different objective function which we sought to minimise. The first objective function gives the final number of bound bacteria, *B*_*B*_(168), the second gives the final total number of bacteria, *B*_*T*_(168), the third gives the mean number of bound bacteria over the week, 〈*B*_*B*_〉, while the fourth gives the mean total number of bacteria over the week, 〈*B*_*T*_〉. We seek to optimise for each objective function individually, rather than performing a multi-objective optimisation, since we wish to find the regimens which fully-optimise each criteria and to compare between these. A unique optimal regimen can always be found under the first two optimality criteria. In those cases where multiple treatment regimens are equally optimal under the third and fourth criteria, we designate that regimen which gives the lowest final value of *B*_*B*_ (for the 〈*B*_*B*_〉 criterion) or *B*_*T*_ (for the 〈*B*_*T*_〉 criterion) as being optimal. We performed separate optimisations for the bound bacterial burden since it is bound bacteria, rather than free bacteria, that actively damage host tissue. Therefore, it may be more important to remove bound bacteria than free bacteria. Further, we performed separate optimisations for the final and mean number of bacteria since we aim both to eliminate the bacterial burden as rapidly as possible (final), while also keeping the bacterial burden low during treatment (mean). In each case we search through the full set of 14,407 possible treatment regimens. We note that, unlike in the steady-state and sensitivity analyses above (with the exception of Fig 7), clearance of free bacteria and free inhibitors is included in these simulations, occurring both in the first 24 hr and in the first 24 hr after each debridement event.

**Fig 9:**
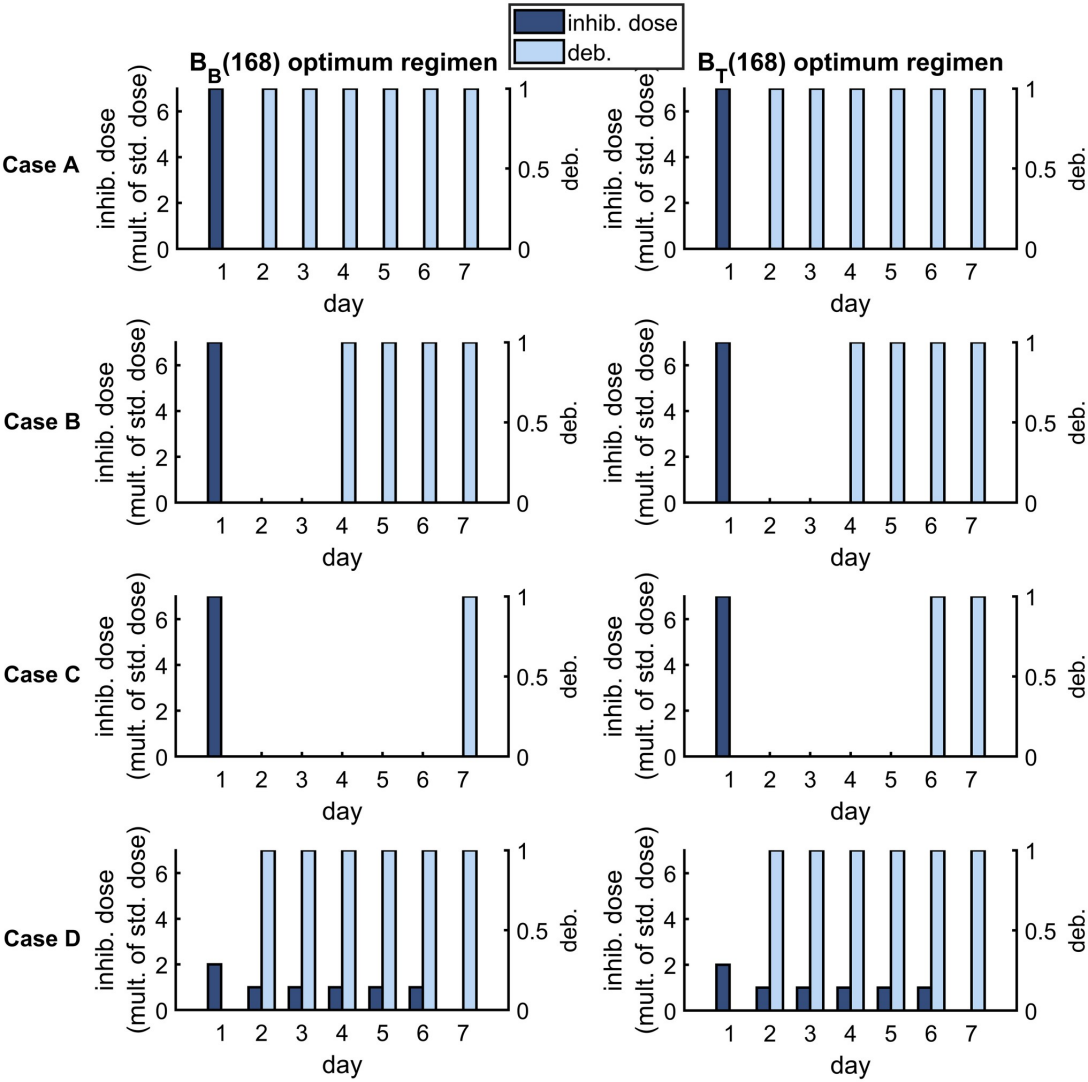
Optimum treatment regimens—minimising final bacterial burdens with a new infection. For each parameter set (rows) the optimum regimen of inhibitor doses and debridement is shown, where we minimise either the final number of bound bacteria, *B*_*B*_(168) (left-hand column), or the final total number of bacteria, *B*_*T*_(168) (right-hand column). Note that inhibitor treatments are plotted as multiples of the standard dose (6.12×10^7^ inhib. cm^−3^). Using all of the inhibitors on the first day is optimal in Cases A–C under both optimality conditions, whereas inhibitor doses should be distributed across the week in Case D. It is optimal to debride every day in Cases A and D, and only on some of the later days in Case B and C. Eqs 1–11 were solved using ode15s, with a constant antibiotic dose. Parameter values: *A* = 8 *μ*g ml^−1^, *λ* = 0 cm^3^cell^−1^hr^−1^, *ρ* = 0 hr^−1^ and *ω* = 1. See Tables 2–4 for the remaining parameter values.

**Fig 10:**
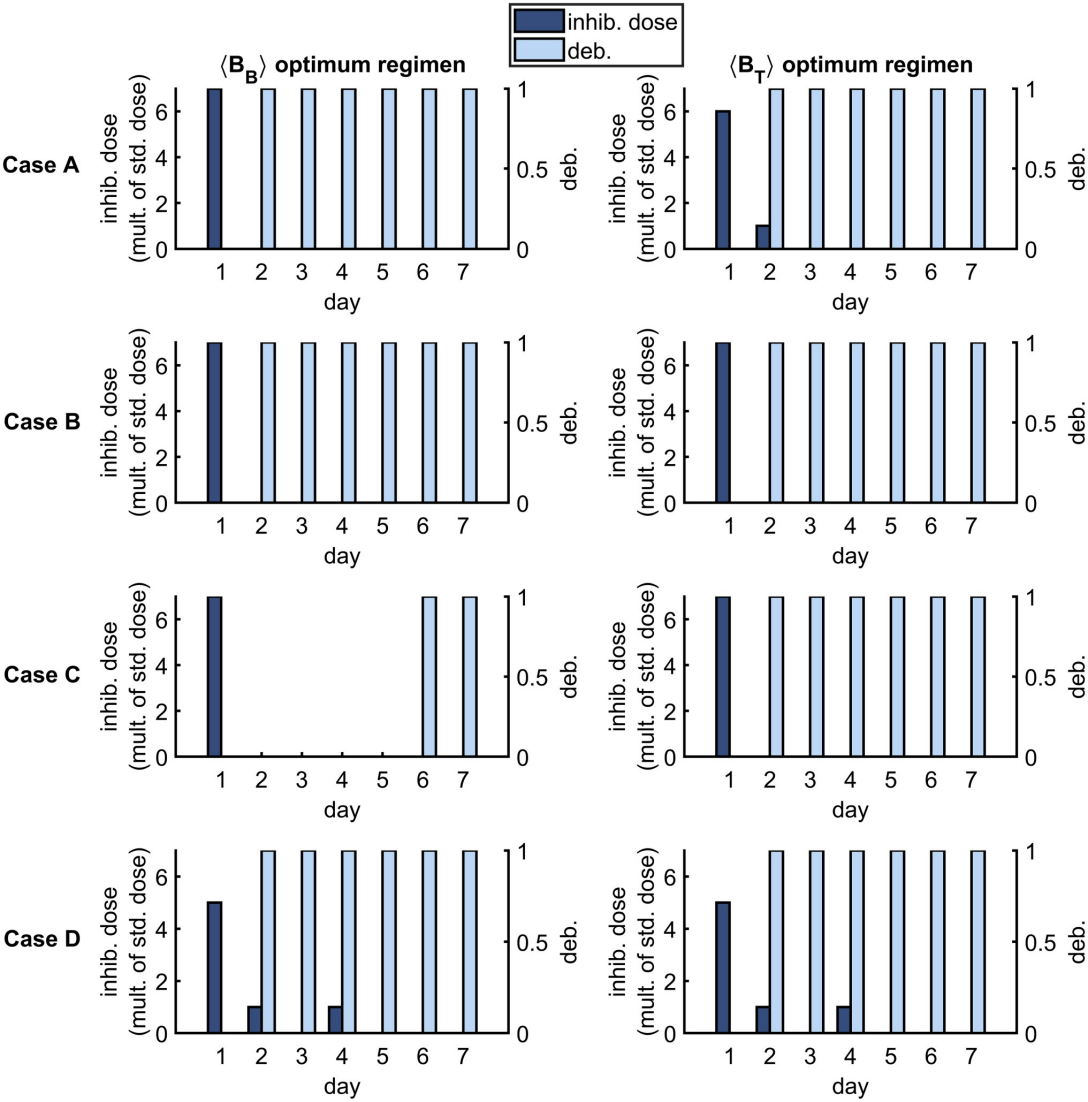
Optimum treatment regimens—minimising mean bacterial burdens with a new infection. For each parameter set (rows) the optimum regimen of inhibitor doses and debridement is shown, where we minimise either the mean number of bound bacteria, 〈*B*_*B*_〉 (left-hand column), or the mean total number of bacteria, 〈*B*_*T*_〉 (right-hand column). Note that inhibitor treatments are plotted as multiples of the standard dose (6.12×10^7^ inhib. cm^−3^). Using most of the inhibitors on the first day is optimal in all instances. It is optimal to debride every day in all cases (A–D), except in Case C under the 〈*B*_*B*_〉 criterion, where it is optimal to debride on days 6 and 7 only. Eqs 1–11 were solved using ode15s, with a constant antibiotic dose. Parameter values: *A* = 8 *μ*g ml^−1^, *λ* = 0 cm^3^cell^−1^hr^−1^, *ρ* = 0 hr^−1^ and *ω* = 1. See Tables 2–4 for the remaining parameter values.

Fig 9 shows the optimum treatment regimens for the *B*_*B*_(168) and *B*_*T*_(168) objective functions (columns) and for each parameter set (rows) in the new infection scenario. It is predicted to be optimal to apply all of the inhibitors at the start of the first day under both optimality conditions in Cases A–C and to distribute inhibitors more evenly across the week in Case D. Further, it is predicted to be optimal to debride every day (days 2–7) under both optimality conditions in Cases A and D, and to debride only on some of the later days in Cases B and C. The results under the 〈*B*_*B*_〉 and 〈*B*_*T*_〉 criteria are similar (see Fig 10).

**Fig 11:**
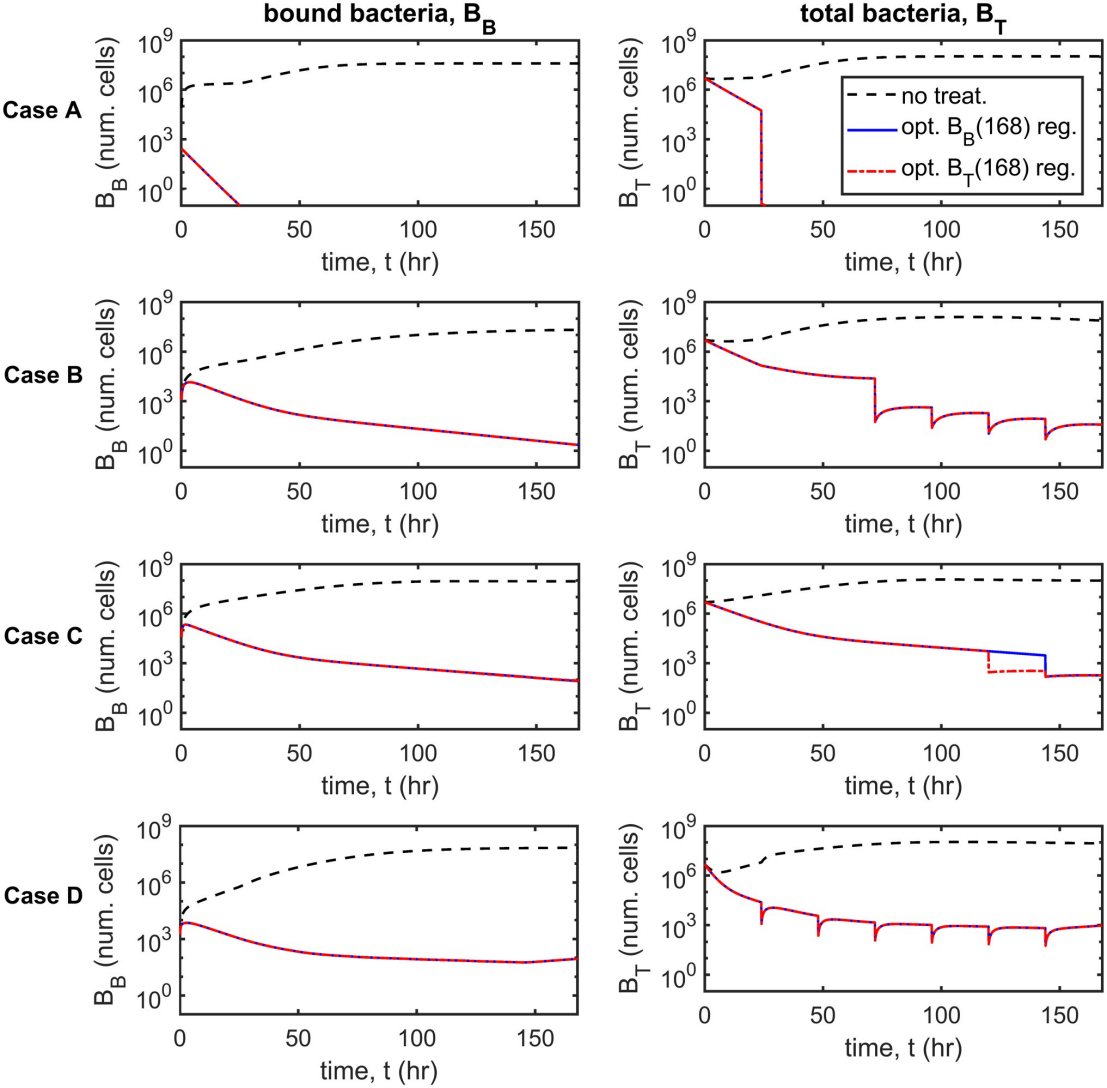
Optimum treatment outcomes—minimising final bacterial burdens with a new infection. Graphs show the dynamics of the bound and total bacterial burdens, *B*_*B*_ and *B*_*T*_ respectively (columns), in the untreated case and under the optimal treatment regimens (see Fig 9) for each parameter set (rows). Note the log_10_ scale on the *y*-axis. The bacterial burden is eliminated (*B*_*T*_(168) < 1) under the optimal treatment regimens in Case A and is significantly reduced in Cases B–D (to *O*(10) in Case B, *O*(10^2^) in Case C and just below 10^3^ in Case D). There is little difference in the effects of the optimum treatments under the different optimality conditions for any given case. Note that the discontinuities in *B*_*T*_ are caused by the instantaneous removal of free bacteria upon debridement (similarly in Figs 12, 15 and 16, see Treatment types). Eqs 1–11 were solved using ode15s, with a constant antibiotic dose. Parameter values: *A* = 8 *μ*g ml^−1^, *λ* = 0 cm^3^cell^−1^hr^−1^, *ρ* = 0 hr^−1^ and *ω* = 1. See Tables 2–4 for the remaining parameter values.

**Fig 12:**
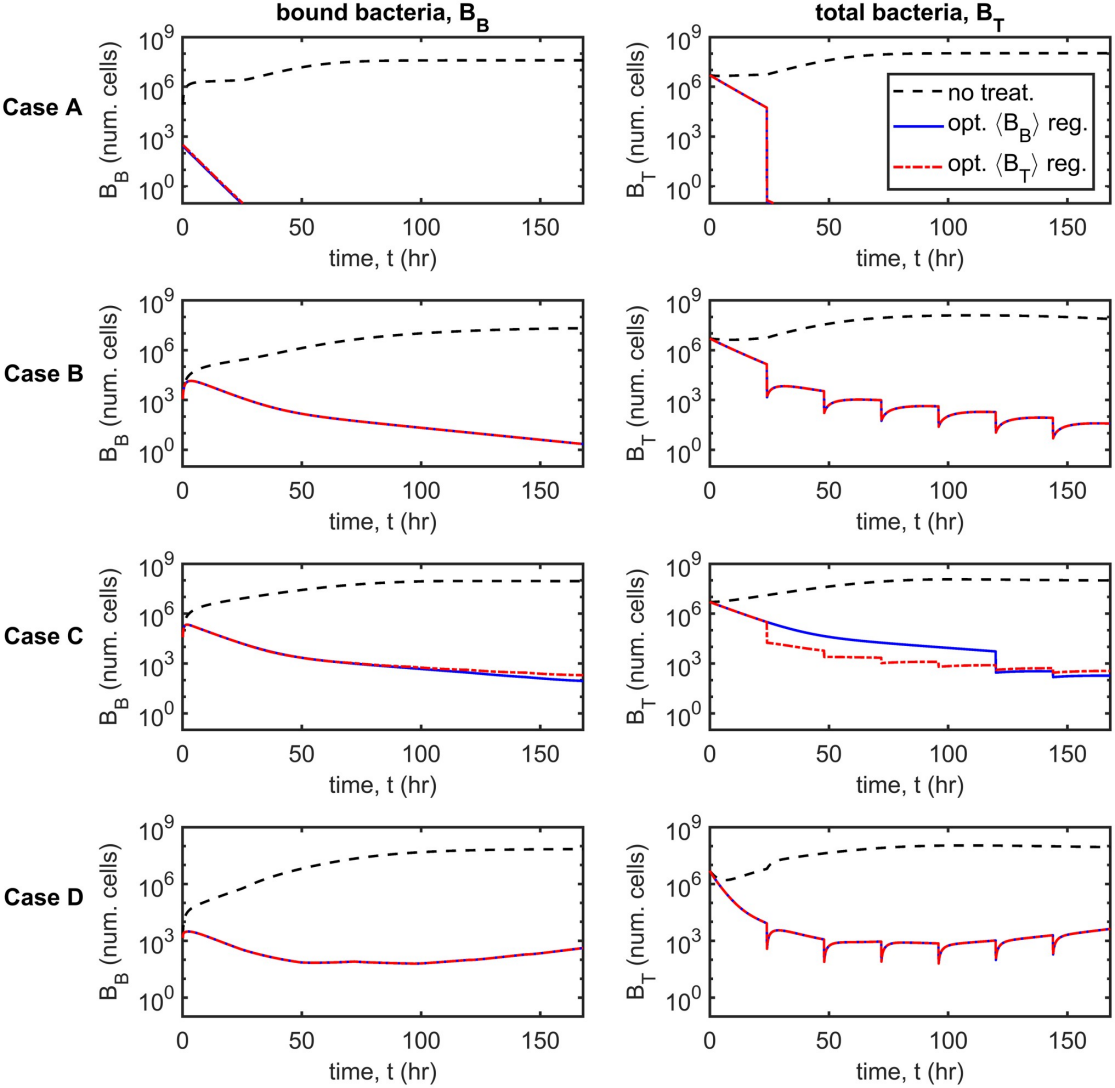
Optimum treatment outcomes—minimising mean bacterial burdens with a new infection. Graphs show the dynamics of the bound and total bacterial burdens, *B*_*B*_ and *B*_*T*_ respectively (columns), in the untreated case and under the optimal treatment regimens (see Fig 10) for each parameter set (rows). Note the log_10_ scale on the *y*-axis. The bacterial burden is eliminated (*B*_*T*_(168) < 1) under the optimal treatment regimen in Case A and almost eliminated in Cases B and C, where *B*_*T*_(168) = *O*(10) and *O*(10^2^) respectively, while *B*_*T*_(168) = *O*(10^4^) in Case D. There is little difference in the effects of the optimum regimens under the different optimality conditions for any given case. Eqs 1–11 were solved using ode15s, with a constant antibiotic dose. Parameter values: *A* = 8 *μ*g ml^−1^, *λ* = 0 cm^3^cell^−1^hr^−1^, *ρ* = 0 hr^−1^ and *ω* = 1. See Tables 2–4 for the remaining parameter values.

Fig 11 shows the dynamics of *B*_*B*_ and *B*_*T*_ under the optimal *B*_*B*_(168) and *B*_*T*_(168) treatment regimens in the new infection scenario. The total bacterial burden is eliminated by the end of the week in Case A, is reduced to *O*(10) in Case B, to *O*(10^2^) in Case C and to just below 10^3^ in Case D (where *B*_*T*_(168) = *O*(10^7^) to *O*(10^8^) in the untreated scenario in Cases A–D). The results under the 〈*B*_*B*_〉 and 〈*B*_*T*_〉 criteria are presented in Fig 12. The difference in the bacterial dynamics between the different optimisation regimens is minor. Both here and in Figs 15 and 16 we plot just the total number of bacteria and the number of bound bacteria for clarity. In both cases the majority of bacteria are susceptible for approximately the first 2 days, after which antibiotic resistant bacteria dominate.

**Fig 13:**
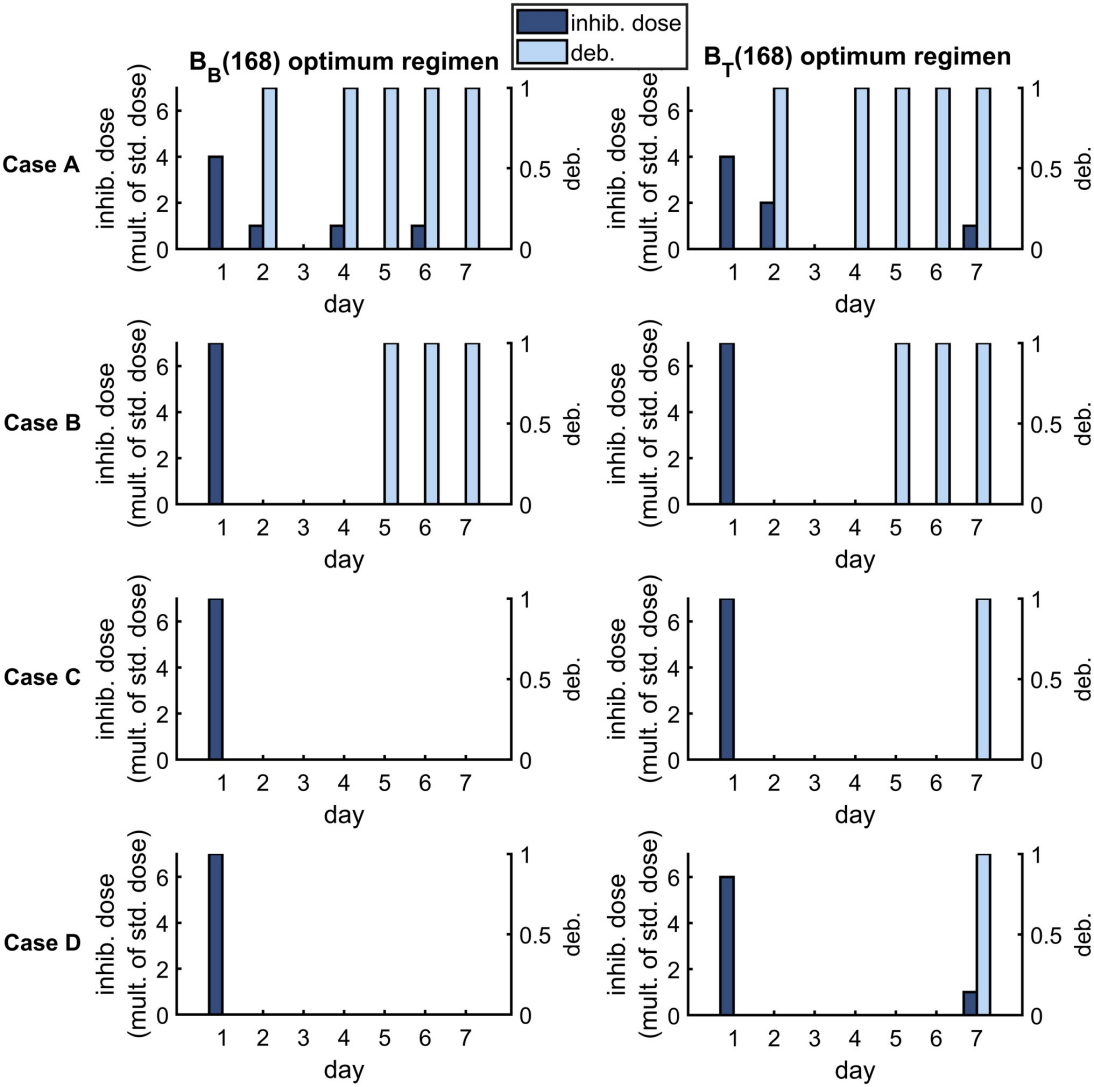
Optimum treatment regimens—minimising final bacterial burdens with an established infection. For each parameter set (rows) the optimum regimen of inhibitor doses and debridement is shown, where we minimise either the final number of bound bacteria, *B*_*B*_(168) (left-hand column), or the final total number of bacteria, *B*_*T*_(168) (right-hand column). Note that inhibitor treatments are plotted as multiples of the standard dose (6.12×10^7^ inhib. cm^−3^). Using most or all of the inhibitors on the first day is optimal in Cases B–D, whereas inhibitor doses should be distributed across the week in Case A. It is optimal to debride on most days in Case A, on the last three days in Case B and either once on the last day (*B*_*T*_(168) optimum) or not at all (*B*_*B*_(168) optimum) in Cases C and D. Eqs 1–11 were solved using ode15s, with a constant antibiotic dose. Parameter values: *A* = 8 *μ*g ml^−1^, *λ* = 0 cm^3^cell^−1^hr^−1^, *ρ* = 0 hr^−1^ and *ω* = 1. The initial conditions are the untreated steady-states for each parameter set, modified so that 2% of the free and bound bacteria are resistant. See Tables 2–4 for the remaining parameter values.

**Fig 14:**
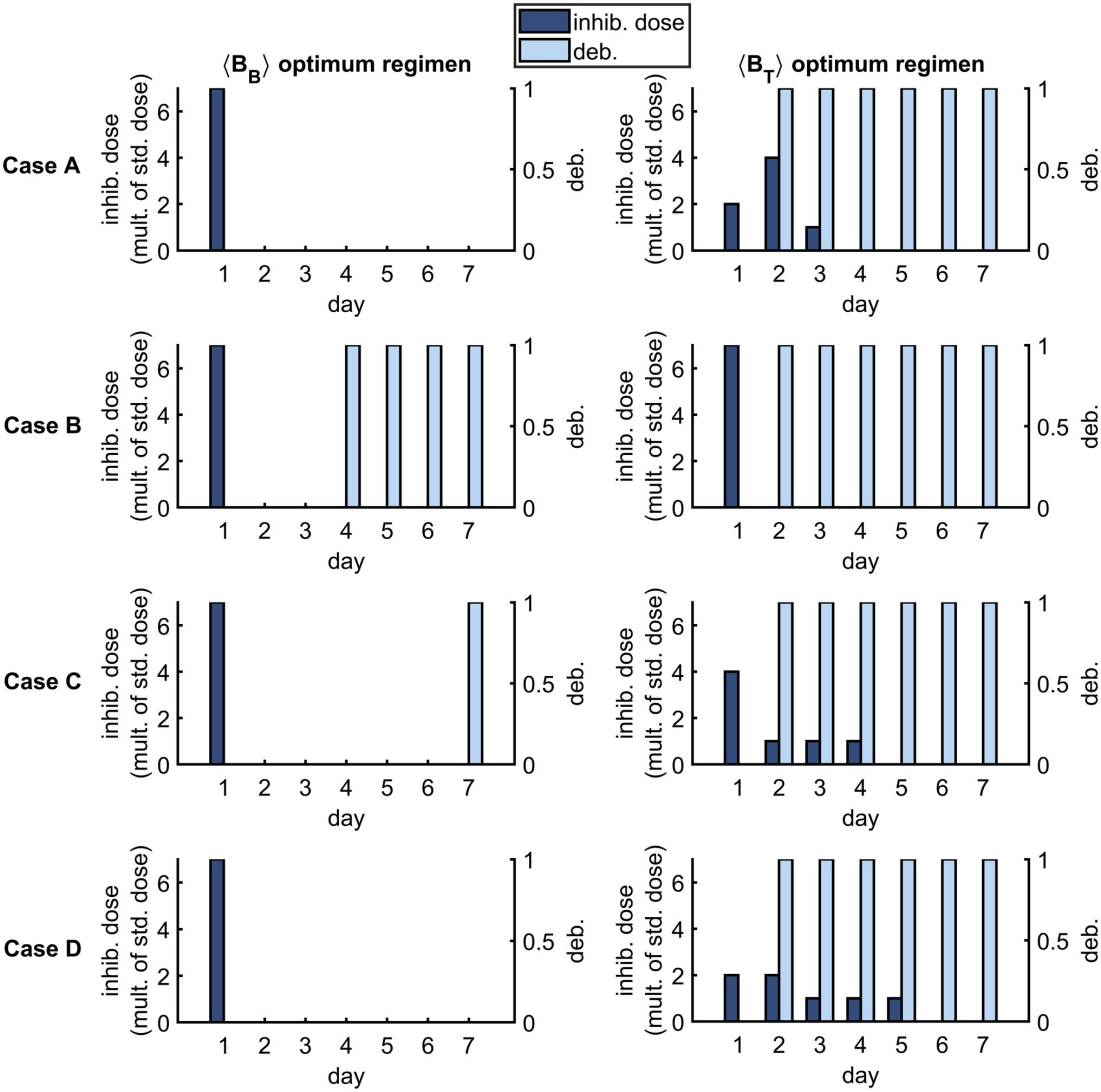
Optimum treatment regimens—minimising mean bacterial burdens with an established infection. For each parameter set (rows) the optimum regimen of inhibitor doses and debridement is shown, where we minimise either the mean number of bound bacteria, 〈*B*_*B*_〉 (left-hand column), or the mean total number of bacteria, 〈*B*_*T*_〉 (right-hand column). Note that inhibitor treatments are plotted as multiples of the standard dose (6.12×10^7^ inhib. cm^−3^). Using all of the inhibitors on the first day is optimal for all cases under the 〈*B*_*B*_〉 criterion and in Case B under the 〈*B*_*T*_〉 criterion, while it is optimal to distribute inhibitor treatment across more of the week in Cases A, C and D under the 〈*B*_*T*_〉 criterion. It is optimal to debride every day in all cases under the 〈*B*_*T*_〉 criterion, while under the 〈*B*_*B*_〉 criterion it is optimal to debride less frequently or not at all. Eqs 1–11 were solved using ode15s, with a constant antibiotic dose. Parameter values: *A* = 8 *μ*g ml^−1^, *λ* = 0 cm^3^cell^−1^hr^−1^, *ρ* = 0 hr^−1^ and *ω* = 1. The initial conditions are the untreated steady-states for each parameter set, modified so that 2% of the free and bound bacteria are resistant. See Tables 2–4 for the remaining parameter values.

Fig 13 shows the optimum treatment regimens for the *B*_*B*_(168) and *B*_*T*_(168) objective functions (columns) and for each parameter set (rows) in the case of an established infection. It is predicted to be optimal to apply all or most of the inhibitors at the start of the first day under both optimality conditions in Cases B–D and to distribute inhibitors more evenly across the week in Case A. In this respect the optimal debridement is similar to the new infection scenario for Cases B and C, differing in Case A, where all inhibitors were used on the first day, and Case D, where inhibitor doses were distributed throughout the week. The predicted optimal debridement patterns differ markedly from the new infection scenario, with debridement being less frequent in all cases and entirely absent in Cases C and D under the *B*_*B*_(168) criterion. The results under the 〈*B*_*B*_〉 and 〈*B*_*T*_〉 criteria differ from all of those discussed above (see Fig 14). Here, it is predicted to be optimal to use all inhibitors at the start of day 1 for Cases A–D under the 〈*B*_*B*_〉 criterion and in Case B under the 〈*B*_*T*_〉 criterion, while it is better to distribute inhibitors across multiple days in Cases A, C and D under the 〈*B*_*T*_〉 criterion. Further it is predicted to be optimal to debride every day in Cases A–D under the 〈*B*_*T*_〉 criterion, not at all in Cases A and D under the 〈*B*_*B*_〉 criterion and only on some later days in Cases B and C under the 〈*B*_*B*_〉 criterion.

**Fig 15:**
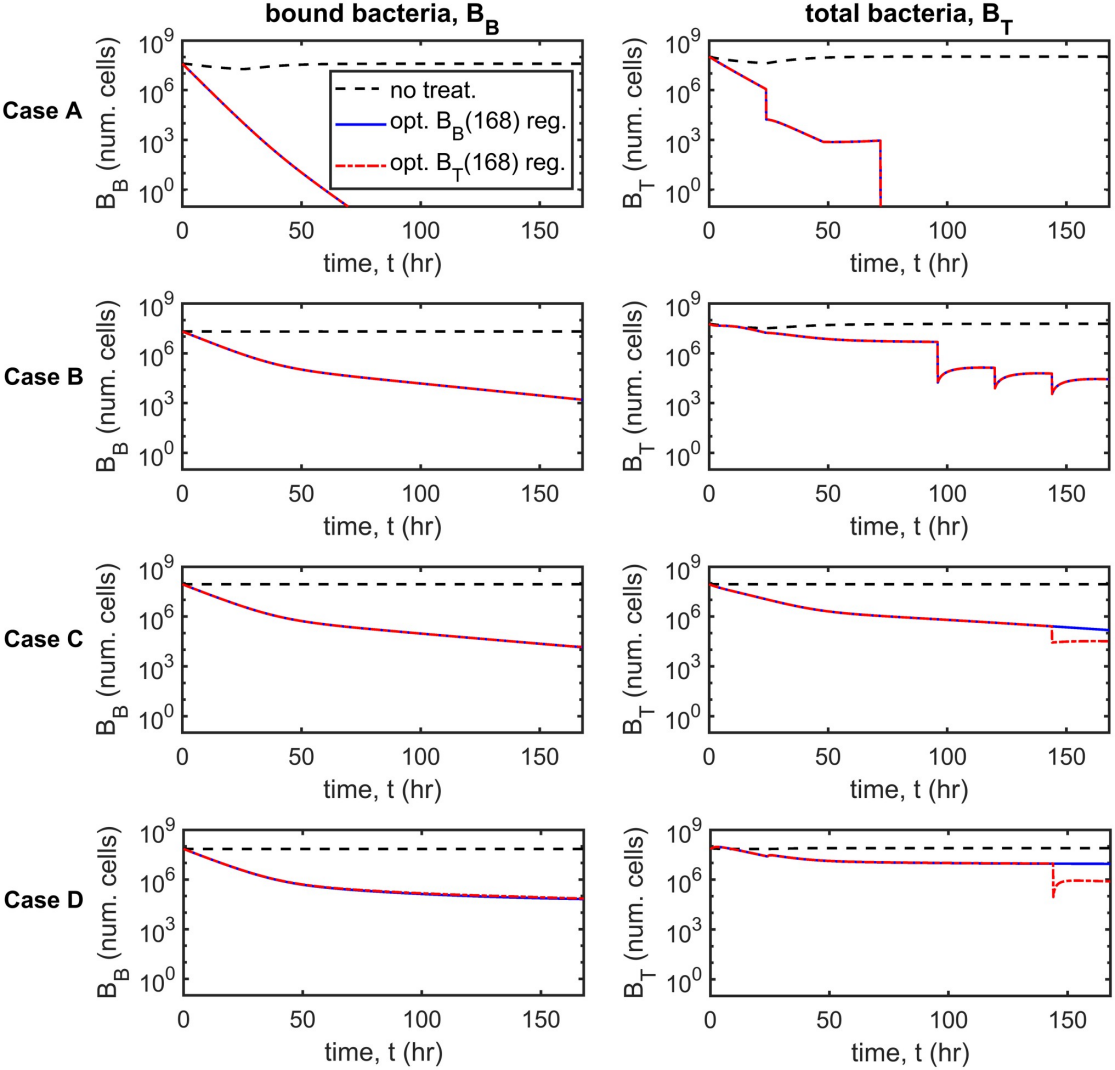
Optimum treatment outcomes—minimising final bacterial burdens with an established infection. Graphs show the dynamics of the bound and total bacterial burdens, *B*_*B*_ and *B*_*T*_ respectively (columns), in the untreated case and under the optimal treatment regimens (see Fig 13) for each parameter set (rows). Note the log_10_ scale on the *y*-axis. The bacterial burden is eliminated (*B*_*T*_(168) < 1) under the optimal treatment regimens in Case A and is significantly reduced in Cases B–D (to *O*(10^2^) in Case B, *O*(10^4^)–*O*(10^5^) in Case C and *O*(10^5^)–*O*(10^6^) in Case D, lower values corresponding to the *B*_*T*_(168) optimality condition and higher values to the *B*_*B*_(168) optimality condition where ranges are given). Eqs 1–11 were solved using ode15s, with a constant antibiotic dose. Parameter values: *A* = 8 *μ*g ml^−1^, *λ* = 0 cm^3^cell^−1^hr^−1^, *ρ* = 0 hr^−1^ and *ω* = 1. The initial conditions are the untreated steady-states for each parameter set, modified so that 2% of the free and bound bacteria are resistant. See Tables 2–4 for the remaining parameter values.

**Fig 16:**
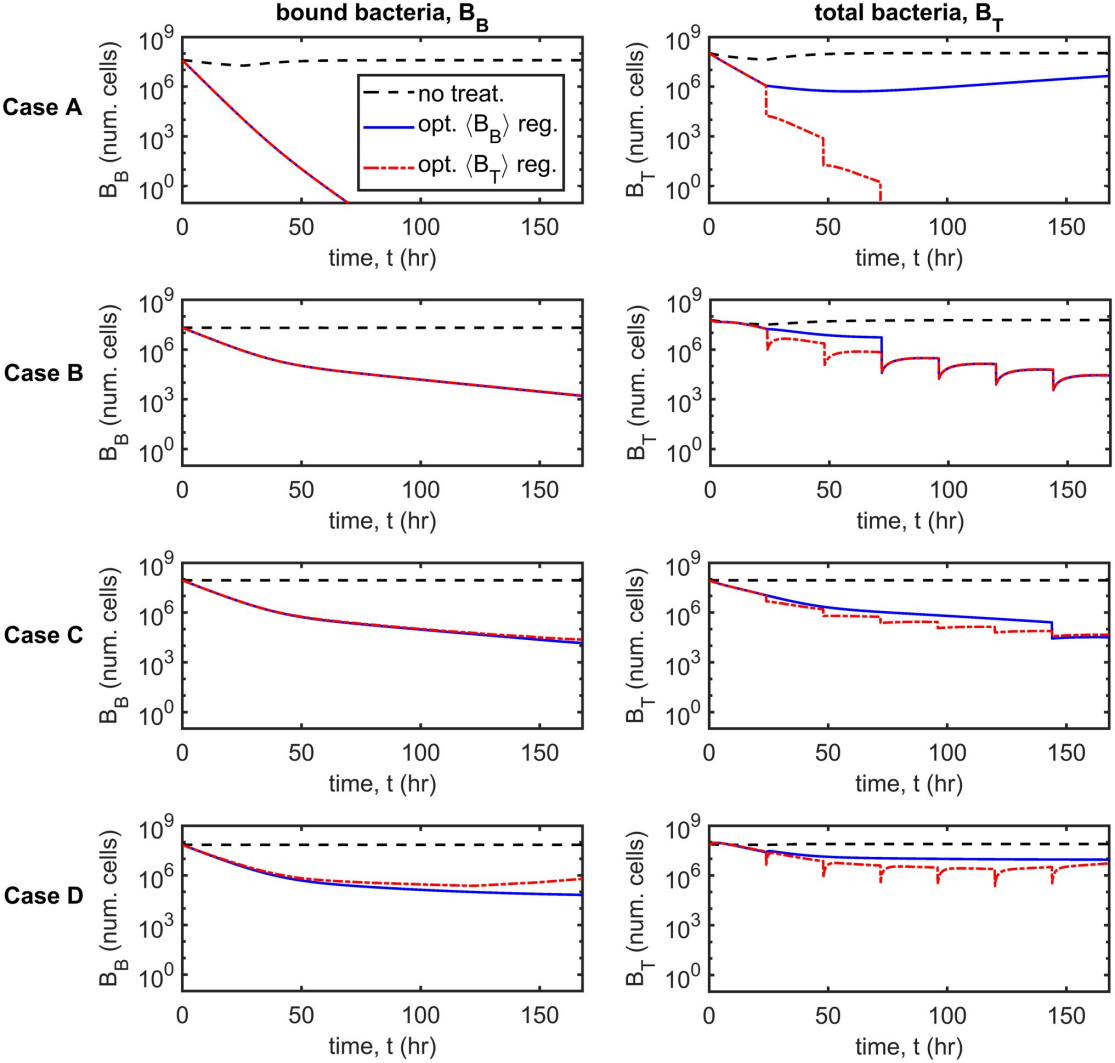
Optimum treatment outcomes—minimising mean bacterial burdens with an established infection. Graphs show the dynamics of the bound and total bacterial burdens, *B*_*B*_ and *B*_*T*_ respectively (columns), in the untreated case and under the optimal treatment regimens (see Fig 14) for each parameter set (rows). Note the log_10_ scale on the *y*-axis. The bacterial burden is eliminated (*B*_*T*_(168) < 1) under the 〈*B*_*T*_〉 optimal treatment regimen in Case A, wile *B*_*T*_(168) = *O*(10^6^) under the 〈*B*_*B*_〉 regimen. Both regimens achieve *B*_*T*_(168) = *O*(10^4^) in Cases B and C, and *B*_*T*_(168) = *O*(10^6^) in Case D. There is little difference in the effects of the optimum regimens under the different optimality conditions for Cases B–D. Eqs 1–11 were solved using ode15s, with a constant antibiotic dose. Parameter values: *A* = 8 *μ*g ml^−1^, *λ* = 0 cm^3^cell^−1^hr^−1^, *ρ* = 0 hr^−1^ and *ω* = 1. The initial conditions are the untreated steady-states for each parameter set, modified so that 2% of the free and bound bacteria are resistant. See Tables 2–4 for the remaining parameter values.

Fig 15 shows the dynamics of *B*_*B*_ and *B*_*T*_ under the optimal *B*_*B*_(168) and *B*_*T*_(168) treatment regimens in the case of an established infection. Here the efficacy is more modest in comparison with the new infection scenario, as would be expected. The total bacterial burden is eliminated by the end of the week in Case A, is reduced to *O*(10^2^) in Case B, to *O*(10^4^)–*O*(10^5^) in Case C and to *O*(10^5^)–*O*(10^6^) in Case D (where *B*_*T*_(168) = *O*(10^7^) to *O*(10^8^) in the untreated scenario in Cases A–D), lower values corresponding to the *B*_*T*_(168) optimality condition and higher values to the *B*_*B*_(168) optimality condition where ranges are given. The results under the 〈*B*_*B*_〉 and 〈*B*_*T*_〉 criteria are presented in Fig 16. The difference in the bacterial dynamics between the different optimisation regimens is minor, except under the 〈*B*_*B*_〉 optimum regimen in Case A, for which the total bacterial burden is not eliminated since debridement is not employed.

## Discussion

The rise in antimicrobial resistance (AMR) poses a real and increasing challenge in treating microbial infections. Anti-adhesion therapy provides one way of meeting this challenge, preventing bacteria from binding to the cells of an infected host, thus rendering them more susceptible to physical clearance e.g. through debridement, and less harmful to the host. In this paper we have used mathematical modelling to elucidate and predict the effects of therapies combining traditional treatments, namely antibiotics and debridement, with anti-adhesion therapy to treat antimicrobial resistant infections. We consider the particular context of a burn wound, infected by a mixture of antibiotic-resistant and antibiotic-susceptible strains of *P. aeruginosa*, using the bactericidal antibiotic meropenem; fitting the antibiotic-associated parameters in our ODE model to *in vitro* data, collected as part of this study. While the parameters used in the model are specific to *P. aeruginosa* and meropenem in a rat burn wound, the model structure can also be used to model burn wound infections in other species (e.g. in humans), with other bactericidal antibiotics and with any bacterial species that uses MAM7 to enable it to bind to host cells (e.g. *Vibrio parahaemolyticus*, *Yersinia pseudotuberculosis* and *Vibrio cholerae* [16]).

We begin by providing a brief summary of our key results, before discussing them in more detail below:

- Treatment with antibiotic in isolation can increase the bacterial burden in some cases (a result of having distinct free and bound compartments);
- Maintaining a constant antibiotic dose is more effective than regular dosing;
- Treatment with antibiotics and inhibitors in combination is more effective than treatment with either therapy in isolation, their combined effect being synergistic (rather than additive);
- The use of inhibitors significantly reduces the minimum antibiotic dose required to clear an infection, thus reducing the chances that bacteria will develop resistance to antibiotic therapies;
- Combining antibiotic and inhibitor therapy with regular debridement further increases treatment efficacy;
- To optimise treatment: keep the antibiotic concentration at its maximum level and
  - *New infection scenario*: use the full inhibitor quota at the start of the first day and debride every subsequent day;
  - *Established infection scenario*: use the full inhibitor quota at the start of the first day and delay debridement for as long as possible, or, if debridement must be conducted more regularly, either use the full inhibitor quota at the start of the first day (Case A and B) or distribute inhibitor doses evenly across the week (Cases C and D).

In each of the results presented we considered four parameter sets, denoted as Cases A–D, each of which provides a good fit to the experimental data, but qualitatively different behaviour beyond the time frame of the experimental results (see Parameter fitting and justification and [26] for details). Steady-state analysis demonstrated that the system is monostable for all parameter sets considered, with one exception (see Steady-state analysis). In the absence of antibiotic, susceptible bacteria survive while antibiotic resistant bacteria go extinct due to the fitness cost associated with resistance. However, in the presence of (sufficient quantities of) antibiotic, resistant bacteria survive and susceptible bacteria go extinct, since the asymmetric killing rate of susceptible and resistant bacteria by antibiotic outweighs the fitness cost experienced by resistant bacteria. Treatment with antibiotics and inhibitors in combination is more effective than treatment with either therapy in isolation, eliminating the bacterial population in Cases B and C, and significantly reducing it in Cases A and D. Interestingly, the combined effect is synergistic, as opposed to additive, effecting a greater reduction in the bacterial burden than the sum of the reductions achieved through either therapy in isolation in Cases B–D and a lesser reduction in Case A. Indeed, the elimination of bacteria in Cases B and C is surprising given that antibiotics alone increase the total bacterial burden, *B*_*T*_, in Case B, while inhibitors alone significantly increase *B*_*T*_ in Case C. While these results are encouraging, it is important to note that it can take on the order of days to months for the system to approach steady-state. Therefore, it is important to consider the dynamic behaviour of the system.

Simulations of the full time-dependent problem revealed that a constant antibiotic concentration is more effective, often significantly so, than regular dosing at the same concentration. This is to be expected, in part, since the antibiotic killing rate is maintained at a high level in the constant concentration scenario, whereas it drops off as antibiotic is eliminated from the body in the regular dosing scenario. However, the difference in efficacy is more significant that might be expected. Combination therapy, combining a constant antibiotic concentration with regular inhibitor dosing and debridement, was the most effective treatment strategy considered, eliminating the bacterial population in Cases A–D in times ranging between 1–30 days. While all bacteria, including the resistant subpopulation, were eliminated in this latter therapy, other strategies were found to increase the number of resistant bacteria, compared with the untreated scenario, in some cases. This highlights the fact that the choice of treatment regimen can have a significant effect on the spread of AMR within a host.

Steady-state sensitivity analyses for antibiotic and inhibitor doses (*A* and $I_{F_{init}}$ respectively) applied in isolation show that these treatments can both decrease and (surprisingly) increase *B*_*T*_, depending upon the dosage used and upon the parameter set under consideration. The increase in *B*_*T*_ is caused either by an increase in the logistic growth rate of bound bacteria (Fig 4 Cases B–D and Fig 5 Case B) or by a decrease in the per-bacteria binding rate of free bacteria to host cells (Fig 5 Case C, see Sensitivity analysis for a detailed discussion). Each treatment is effective in reducing the total bacterial burden when used in isolation, provided the dosage is sufficiently large; however, our model predicts that the antibiotic dose would have to be made infeasibly large in Case B to be effective in isolation (*A* > 10 *μ*g cm^−3^) and similarly for the inhibitor dose in Cases C and D (hundreds to thousands of times the standard dose). Further experimental studies are required to test these predictions to determine under what circumstances they hold.

Sensitivity analysis for antibiotic and inhibitor combination therapy without debridement predicts that the bacterial burden can be eliminated within four weeks in Case C using realistic doses and significantly reduced in Case B, whereas *B*_*T*_ can be reduced by at most an order of magnitude for realistic doses in Cases A and D, which are less sensitive to treatment. We further predicted that treatment efficacy can be enhanced by including debridement, eliminating bacteria in Cases A–C using realistic levels of antibiotic and inhibitor, and clearing an infection more rapidly. Importantly, our model predicts that the use of inhibitors significantly reduces the antibiotic dose required to clear an infection, both in terms of the maximum antibiotic concentration required and also in terms of the total quantity of antibiotic administered over the course of an infection, given that combination therapy may clear an infection more quickly. We speculate that this could also reduce the chances of bacteria developing resistance to antibiotic therapies.

The model is insensitive to the rates of conjugation and segregation (*λ* and *ρ* respectively) within realistic ranges; hence, it is reasonable to neglect these processes from the model. By contrast, the system is sensitive to the factor difference in antibiotic potency against bound bacteria compared with free bacteria, *ω*, an increase in this parameter effecting a decrease in *B*_*T*_. We have assumed that *ω* = 1 in the present work; however, it would be valuable to measure this parameter experientially for different bacterial species, antibiotics and infection sites to determine its true value in a variety of contexts, and thus to incorporate this into future models.

Optimal treatment regimens, combining antibiotics and inhibitors with debridement over the period of a week, were predicted for Cases A–D. For each case, two scenarios were considered: the first, corresponding to a new infection, in which bacteria have not yet had an opportunity to bind to host cells and the second, corresponding to an established infection, including both bound and free bacteria. Both scenarios consisted of mixed populations of susceptible and resistant bacteria. The inhibitor dosing and debridement regimens were allowed to vary, while the antibiotic concentration was assumed to take its maximum value based upon the preceding sensitivity analyses. Four separate optimisations were performed for each parameter set and scenario using different optimality criteria: minimising the final bound bacterial burden, *B*_*B*_(168), minimising the final total bacterial burden, *B*_*T*_(168), minimising the mean bound bacterial burden, 〈*B*_*B*_〉, and minimising the mean total bacterial burden, 〈*B*_*T*_〉. We focused upon bound bacteria in particular, since it is bound bacteria, rather than free bacteria, that damage host cells.

In the new infection scenario it is almost always optimal to use the full weekly quota of inhibitors at the beginning of the first day in Cases A–C and to distribute inhibitor dosing more evenly across the week in Case D, while it is best to debride every day to minimise 〈*B*_*B*_〉 and 〈*B*_*T*_〉 in most cases, the optimal debridement regimen varying between parameter sets under the *B*_*B*_(168) and *B*_*T*_(168) criteria. Given that the bound and total bacterial burdens evolve similarly in Cases A–D under each of the optimal regimens, we suggest that, in the new infection scenario, it would be best to use the full inhibitor quota at the beginning of the first day and to debride every day in a clinical setting. The optimal treatment regimens are predicted to eliminate the bacterial burden within a week in Case A and to significantly reduce the bacterial burden in Cases B–D. Further experimental studies are required to test these predictions.

In the established infection scenario optimal treatment regimens vary greatly between parameter sets and optimality criteria. Since it is most important that we eliminate the bound bacterial burden, we suggest that a regimen which minimises *B*_*B*_(168) or 〈*B*_*B*_〉 would be best. Under these criteria it is almost always optimal to use the full inhibitor quota at the beginning of the first day of treatment and to delay the first debridement event for as long as possible to allow inhibitors time to outcompete bacteria for binding sites before debridement removes their free contingent. If daily debridement is required then the optimal strategy will depend upon the parameter set. In Cases A and B, the ratio of bacterial binding to unbinding rates, *α*_*Bac*_/*β*_*bac*_, is lower than the ratio of inhibitor binding to unbinding rates, *α*_*I*_/*β*_*I*_. Therefore, inhibitors quickly outcompete bacteria for binding sites, such that using the full inhibitor dose at the start of the first day would be a good strategy. In Cases C and D, *α*_*Bac*_/*β*_*bac*_ > *α*_*I*_/*β*_*I*_. Therefore, it takes inhibitors longer to displace bacteria, such that distributing inhibitor doses evenly across the week would be a good strategy.

In future work we will develop our mathematical modelling in a number of new directions. This will include the development of partial differential equation models to account for the spatial distribution of bacteria, antibiotics, inhibitors and binding sites (ODE models being incapable of adequately accounting for non-uniform distributions or diffusive/migratory processes), allowing us to investigate issues such as how a localised application of inhibitors would affect treatment efficacy; the development of stochastic and cellular automata models to account for the random behaviour of the system at a more finely-resolved spatial scale; and a more detailed stability analysis of ODE systems involving treatment with inhibitors and antibiotics. Future models could also consider the use of bacteriostatic antibiotics, quorum sensing and biofilm formation. Possible future experimental studies are noted in the discussion above.

In conclusion, our model predicts that antibiotics and inhibitors have a synergistic effect when used together, that combination therapy is more effective than either treatment in isolation and that treatment may be further enhanced through the use of debridement. Further, our model predicts that, in general, when treating over the period of a week, the optimal strategy is to maintain a constant antibiotic dose at the maximum allowable concentration, to use the full quota of inhibitors at the beginning of the first day of treatment and to debride daily, though this could be further enhanced if a patient-specific parameter set is identified. Lastly, our models predict that using inhibitors lowers the minimum antibiotic dose required in order to eliminate a bacterial infection, reducing the selection pressure and, potentially, the probability that bacteria will develop resistance to the antibiotic.

## Supporting information

S1 Text Fitting parameters to *in vitro* data. (PDF)

S2 Text Steady-state analysis details. (PDF)

S3 Text Detailed numerical results. (PDF)
