# Supplementary material for: Mathematical model predicts anti-adhesion–antibiotic–debridement combination therapies can clear an antibiotic resistant infection
Source: PLoS Comput Biol. 2019 Jul 23;15(7):e1007211. doi: 10.1371/journal.pcbi.1007211 (PMC6677339; doi:10.1371/journal.pcbi.1007211)
Supplement: S1 Text — (PDF) [file pcbi.1007211.s001.pdf]

Mathematical model predicts anti-adhesion–antibiotic–debridement combination therapies can clear an antibiotic resistant infection
PLOS Computational Biology
S1 Text

Paul A. Roberts*[1,2], Ryan M. Huebinger[3], Emma Keen[2], Anne-Marie Krachler[4] and Sara Jabbari[1,2]

[1]School of Mathematics, University of Birmingham, Edgbaston, Birmingham, United Kingdom
[2]Institute of Microbiology and Infection, School of Biosciences, University of Birmingham, Edgbaston, Birmingham, United Kingdom
[3]Department of Surgery, University of Texas Southwestern Medical Center, Dallas, Texas, United States of America
[4]Department of Microbiology and Molecular Genetics, University of Texas McGovern Medical School at Houston, Houston, Texas, United States of America

Fitting parameters to *in vitro* data

In what follows we describe the experiments and fitting procedures used to determine the antibiotic-associated parameters in our mathematical model. The bacterial population was measured in terms of optical density (OD600) in our experiments; however, we need to know the number of bacteria in terms of colony-forming units (CFUs) to allow comparison with simulations. Therefore, we begin by calibrating OD600 to CFUs.

Optical density calibration

A culture of PA1004 WT (wild-type) *Pseudomonas aeruginosa* (*P. aeruginosa*) were grown overnight in LB (lysogeny broth). The culture was then diluted to an OD600 of 1.0, from which dilutions of 0.2, 0.4, 0.6 and 0.8 were prepared. Each dilution was then serially diluted and 10 μL of each dilution plated onto LB-agar plates and incubated overnight at 37°C. Colonies were then counted and CFU cm^{-3} calculated. Three independent experiments were performed, each of which was conducted in triplicate, to give $n = 9$ measurements at each dilution. Data were normalised such that an OD600 of 0 corresponds to 0 CFU cm^{-3}.

Assuming that the relationship between OD600 and CFU cm^{-3} is linear, the Matlab routine fminsearch was used to fit a straight line with zero intercept to the mean of the data, minimising the mean squared error (MSE), to give the relationship Y (CFU cm^{-3}) = $7.19\times10^{8}X$ (OD600) (see Fig A). This value is in good agreement with Kim *et al.* [1] who measured Y (CFU cm^{-3}) = $2.0\times10^{8}X$ (OD600) $+4.0\times10^{6}$ for *P. aeruginosa* strain KCCM-40269 in LB.

Directed evolution of a clonal meropenem resistant mutant of PA1004

The *P. aeruginosa* isolate PA1004 is a clinical isolate derived from a hospital-associated outbreak at a burns care ward [2]. It is fully susceptible according to the European Committee on Antimicrobial Susceptibility Testing (EUCAST)

*Corresponding author
E-mail address: p.a.roberts@univ.oxon.org (PAR)

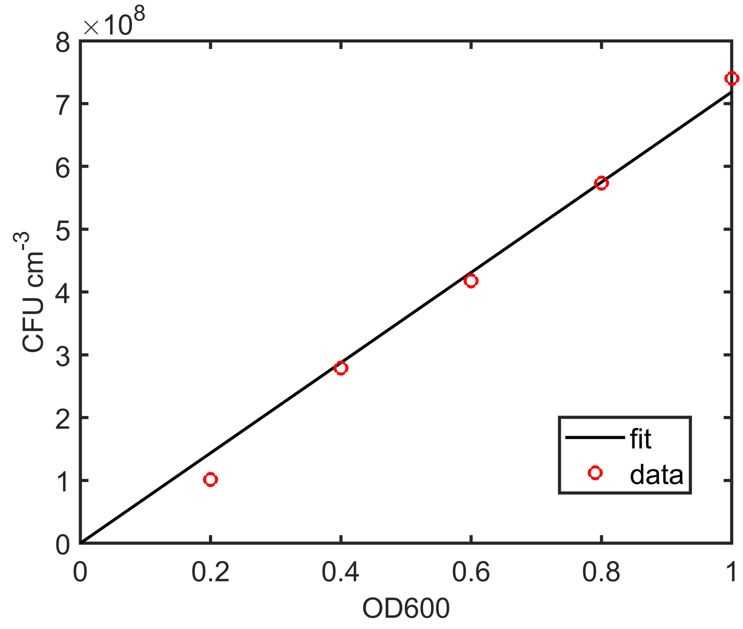

Figure A: **OD600 calibration.** There is a close fit between the fitted straight line, Y (CFU cm^{-3}) = $7.19 \times 10^8 X$ (OD600), and the mean experimental data points.

clinical breakpoint for meropenem of 2 μg cm^{-3}. To experimentally assess a system consisting of a susceptible and resistant, but otherwise clonal bacterial strain pair, we used the PA1004 isolate as a basis to evolve a meropenem resistant mutant. A resistant strain is defined per EUCAST as having an MIC $\geq$ 8 μg cm^{-3}. We plated 100 μL of an overnight culture of PA1004 onto an LBA (lysogeny broth agar) plate containing 2 μg cm^{-3} of meropenem and incubated overnight at 37°C. Following this incubation, several colonies were picked, grown in LB without meropenem for 16 hours and then plated onto LBA plates containing 2 μg cm^{-3} of meropenem again, to ensure the resistance was maintained. This procedure was repeated using increasing concentrations of meropenem (6, 8 and finally 10 μg cm^{-3}) for selection. Stocks of all intermediates were made and characterized by spot plating on increasing concentrations of meropenem (Fig B). The final evolved strain was designated PA1004 Evo10.

Bacterial growth curve experiments

Growth curves were measured for the susceptible and resistant strains of *P. aeruginosa*, PA1004 WT and PA1004 Evo10, under a range of concentrations of the antibiotic meropenem. Experiments were performed using the growth medium DMEM (Dulbecco's modified eagle medium) to replicate the nutrient levels in a burn wound exudate [3, 4].

Cultures of each strain were centrifuged overnight and then suspended in DMEM. Cultures were normalised to an OD600 of 0.4 (twice the desired starting OD since it is diluted in a ratio of 1:2 later in the experiment) using DMEM. Dilutions of meropenem were also prepared in DMEM at twice the desired concentration. In each experiment, 500 μL of culture and 500 μL of meropenem dilution were added to the wells of a 24 well plate and OD600 measurements taken using a plate reader every 10 minutes for 24 hours. Between readings the plate was incubated at 37°C and shaken at 200 rpm. Experiments were performed in triplicate (n = 3) at meropenem concentrations of 0, 1, 2, 4, 8 and 16 μg cm^{-3} for each strain in isolation (see Fig C).

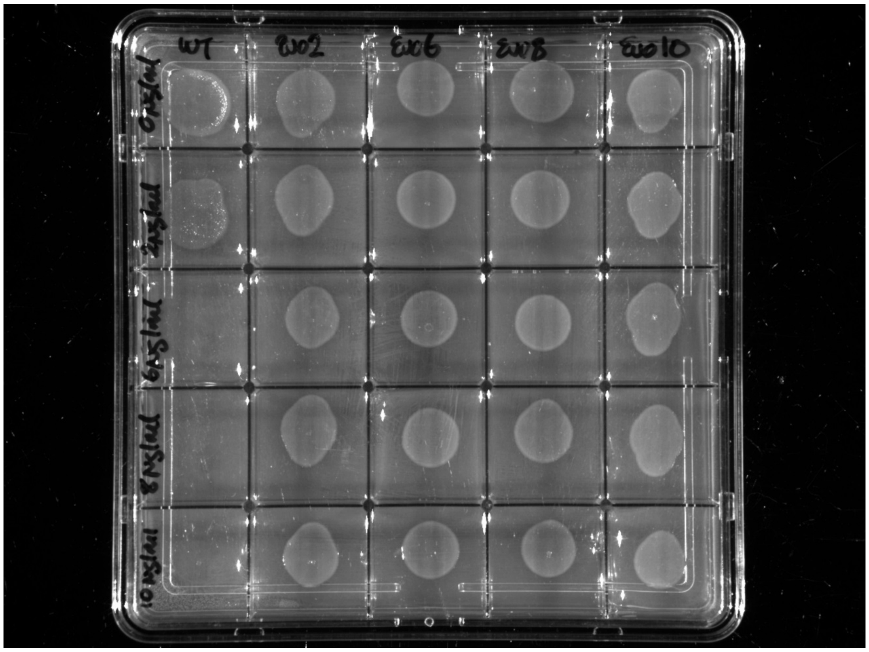

Figure B: **Directed evolution and spot growth assay to characterize PA1004 and evolved meropenem resistant derivatives.** Left to right: original WT strain PA1004 and derivatives Evo 2, 6, 8 and Evo10. Top to bottom: 0, 1, 6, 8, and 10 μg cm^{-3} meropenem in LBA.

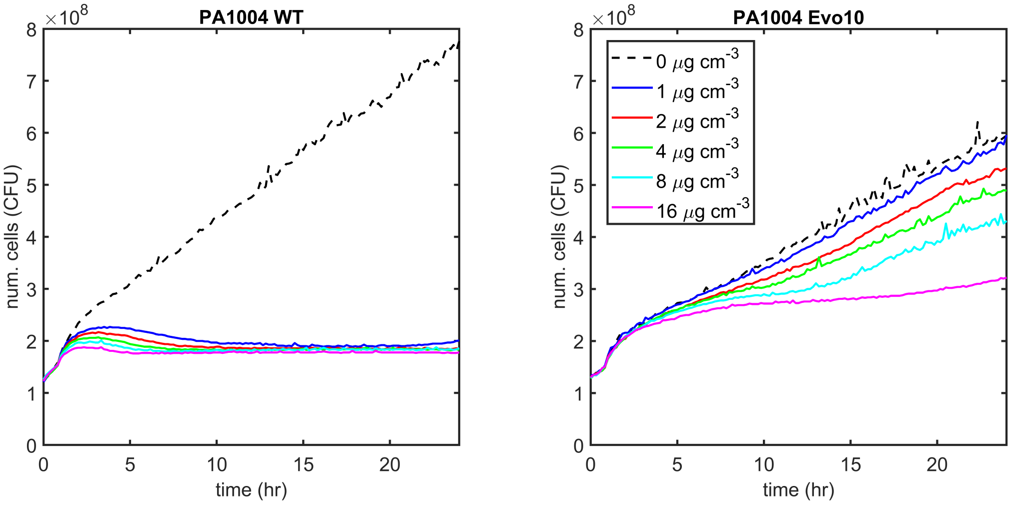

Figure C: **Experimental growth curves for *P. aeruginosa* strains PA1004 WT and PA1004 Evo10 under a range of meropenem concentrations.** Panels show the mean number of bacteria (n = 3) in CFUs over time for the PA1004 WT (left panel) and PA1004 Evo10 (right panel) strains under meropenem concentrations of 0, 1, 2, 4, 8 and 16 μg cm^{-3}. Bacterial population growth decreases with increasing meropenem concentration, the effect upon PA1004 WT (the susceptible strain) being more dramatic than that upon PA1004 Evo10 (the resistant strain). Note also that the PA1004 WT strain grows more rapidly in the absence of antibiotics than the PA1004 Evo10 strain, indicating that the latter strain has incurred a fitness cost.

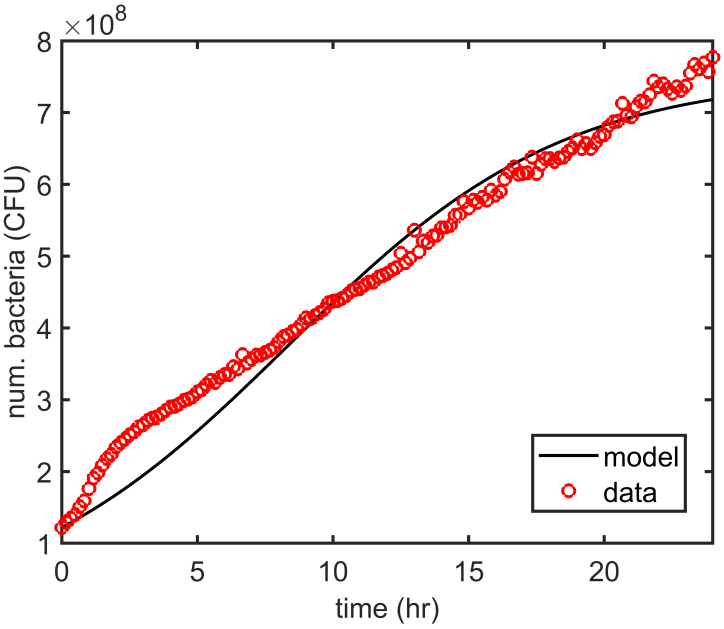

Figure D: **Comparison of the fitted model with experimental data.** The logistic growth equation provides a good fit to the mean experimental data points for susceptible bacteria (PA1004 WT) without antibiotic. Parameter vales: $r = 0.196$ hr^{-1} and $K = 7.51 \times 10^8$ cells cm^{-3}.

Fitting to growth curves

We used the growth curve data described above to fit five of our model parameters: the fitness cost, c, the maximal killing rates of susceptible and resistant bacteria by antibiotics, E^S_{max} and E^R_{max}, and the antibiotic concentrations at which the killing rate of susceptible and resistant bacteria is half maximal, A^S_{50} and A^R_{50}. We also used the fitting to inform our choice of the elimination rate of antibiotic, δ_A, which accounts for natural degradation (an aspect captured in our *in vitro* experiments), degradation due to the action of biological molecules and elimination from the body e.g. via the bloodstream, the latter two of which are not captured by our *in vitro* experiments. Parameters were fitted to the data sequentially, as described below, fitting to the mean of the data using the Matlab routine fminsearch to minimise the MSE in all cases. Fitting all of the parameters to all of the data simultaneously was also considered; however, better fits were found to be obtained by taking a sequential approach. Parameter fits correspond to unique global minima in the MSE within the range of biologically realistic values in all cases.

Stage 1: we fitted a logistic growth equation to the growth curve for susceptible (PA1004 WT) bacteria without antibiotic, with intrinsic growth rate $r = 0.196$ hr^{-1} and carrying capacity $K = 7.51 \times 10^8$ cells cm^{-3} (see Fig D). We note that these values are different from the *in vivo* values used in the paper; however, it is necessary to calculate the *in vitro* values in order to fit the antibiotic-associated parameters.

Stage 2: we constructed a modified equation combining the logistic growth term from the first stage, using the values for r and K established in Stage 1, with an antibiotic killing term of the form used in Eqs 1–4, with parameters E^S_{max} and A^S_{50}. This equation was fitted to the data for susceptible bacteria exposed to 1, 2, 4, 8 and 16 μg cm^{-3} of meropenem.

Stage 3: we further modified the equation from Stage 2 to include an antibiotic decay term with decay rate δ. It was found that the best fit was obtained for $\delta = 0$ hr^{-1}. Therefore, the natural decay rate of antibiotic does not contribute to the elimination rate of antibiotic, δ_A, in Eq 7.

Stage 4: returning to the logistic growth equation from Stage 1, we modified this equation to incorporate a fitness cost, c, as in Eqs 2–4. Fixing r and K to the values obtained in Stage 1, we fitted this modified equation to the mean growth curve for resistant bacteria (PA1004 Evo10) without antibiotic.

Stage 5: in this final stage of fitting we did the same as in Stage 2, but for resistant bacteria, adding an antibiotic killing term, with parameters E^R_{max} and A^R_{50}, to the equation from Stage 4 and fitting to the data for resistant bacteria exposed to 1, 2, 4, 8 and 16 μg cm^{-3} of meropenem.

References

[1] Kim D, Chung S, Lee S, Choi J. Relation of microbial biomass to counting units for Pseudomonas aeruginosa. Afr J Microbiol Res. 2012;6(21):4620–4622.

[2] Quick J, Cumley N, Wearn CM, Niebel M, Constantinidou C, Thomas CM, et al. Seeking the source of Pseudomonas aeruginosa infections in a recently opened hospital: an observational study using whole-genome sequencing. BMJ Open. 2014;4(11):e006278.

[3] Cutting K. Wound exudate: composition and functions. Br J Community Nurs. 2003;8:4–9.

[4] Gonzalez MR, Fleuchot B, Lauciello L, Jafari P, Applegate LA, Raffoul W, et al. Effect of Human Burn Wound Exudate on Pseudomonas aeruginosa Virulence. mSphere. 2016;1(2):e00111–15.
