# Supplementary material for: Mathematical model predicts anti-adhesion–antibiotic–debridement combination therapies can clear an antibiotic resistant infection
Source: PLoS Comput Biol. 2019 Jul 23;15(7):e1007211. doi: 10.1371/journal.pcbi.1007211 (PMC6677339; doi:10.1371/journal.pcbi.1007211)
Supplement: S2 Text — (PDF) [file pcbi.1007211.s002.pdf]

Mathematical model predicts anti-adhesion–antibiotic–debridement combination therapies can clear an antibiotic resistant infection
PLOS Computational Biology
S2 Text

Paul A. Roberts*[1,2], Ryan M. Huebinger[3], Emma Keen[2], Anne-Marie Krachler[4] and Sara Jabbari[1,2]

[1]School of Mathematics, University of Birmingham, Edgbaston, Birmingham, United Kingdom
[2]Institute of Microbiology and Infection, School of Biosciences, University of Birmingham, Edgbaston, Birmingham, United Kingdom
[3]Department of Surgery, University of Texas Southwestern Medical Center, Dallas, Texas, United States of America
[4]Department of Microbiology and Molecular Genetics, University of Texas McGovern Medical School at Houston, Houston, Texas, United States of America

*Corresponding author
E-mail address: p.a.roberts@univ.oxon.org (PAR)

Steady-state analysis details

We simplify Eqs 1–7 as described in the main text, retaining only those terms required for a steady-state analysis, reducing them to the following form:

$$\frac{\mathrm{d}B_{F_S}}{\mathrm{d}t} = \underbrace{r_{F_S} B_{F_S}\left(1-\frac{B_{F_S}+B_{F_R}}{K_F}\right)}_{\text{logistic growth}} + \underbrace{(1-\eta(E))H(K_B-B_{B_S}-B_{B_R})\frac{1}{h}r_{B_S}B_{B_S}\left(1-\frac{B_{B_S}+B_{B_R}}{K_B}\right)}_{\text{daughter cells freed from host cells upon division}}$$
$$-\underbrace{\alpha_{Bac}A_r B_{F_S}E}_{\text{binding to host cells}} + \underbrace{\frac{\beta_{Bac}}{h}B_{B_S}}_{\substack{\text{unbinding}\\ \text{from host cells}}} - \underbrace{\frac{E^S_{max}A}{A^S_{50}+A}B_{F_S}}_{\substack{\text{killing by}\\ \text{antibiotic}}}, \tag{A}$$

$$\frac{\mathrm{d}B_{F_R}}{\mathrm{d}t} = \underbrace{(1-cH(K_F-B_{F_S}-B_{F_R}))r_{F_S}B_{F_R}\left(1-\frac{B_{F_S}+B_{F_R}}{K_F}\right)}_{\text{logistic growth}}$$
$$+\underbrace{(1-\eta(E))H(K_B-B_{B_S}-B_{B_R})\frac{1}{h}(1-c)r_{B_S}B_{B_R}\left(1-\frac{B_{B_S}+B_{B_R}}{K_B}\right)}_{\text{daughter cells freed from host cells upon division}}$$
$$-\underbrace{\alpha_{Bac}A_r B_{F_R}E}_{\text{binding to host cells}} + \underbrace{\frac{\beta_{Bac}}{h}B_{B_R}}_{\substack{\text{unbinding}\\ \text{from host cells}}} - \underbrace{\frac{E^R_{max}A}{A^R_{50}+A}B_{F_R}}_{\substack{\text{killing by}\\ \text{antibiotic}}}, \tag{B}$$

$$\frac{\mathrm{d}B_{B_S}}{\mathrm{d}t} = \underbrace{\Big(1+(\eta(E)-1)H(K_B-B_{B_S}-B_{B_R})\Big)r_{B_S}B_{B_S}\left(1-\frac{B_{B_S}+B_{B_R}}{K_B}\right)}_{\text{logistic growth (a proportion, }\eta\text{, remain attached)}}$$
$$+\underbrace{\alpha_{Bac}VB_{F_S}E}_{\text{binding to host cells}} - \underbrace{\beta_{Bac}B_{B_S}}_{\substack{\text{unbinding}\\ \text{from host cells}}} - \underbrace{\delta_B B_{B_S}}_{\text{phagocytosis}} - \underbrace{\frac{E^S_{max}A}{A^S_{50}+A}B_{B_S}}_{\substack{\text{killing by}\\ \text{antibiotic}}}, \tag{C}$$

$$\frac{\mathrm{d}B_{B_R}}{\mathrm{d}t} = \underbrace{\Big(1+(\eta(E)-1)H(K_B-B_{B_S}-B_{B_R})\Big)(1-cH(K_B-B_{B_S}-B_{B_R}))r_{B_S}B_{B_R}\left(1-\frac{B_{B_S}+B_{B_R}}{K_B}\right)}_{\text{logistic growth (a proportion, }\eta\text{, remain attached)}}$$
$$+\underbrace{\alpha_{Bac}VB_{F_R}E}_{\text{binding to host cells}} - \underbrace{\beta_{Bac}B_{B_R}}_{\substack{\text{unbinding}\\ \text{from host cells}}} - \underbrace{\delta_B B_{B_R}}_{\text{phagocytosis}} - \underbrace{\frac{E^R_{max}A}{A^R_{50}+A}B_{B_R}}_{\substack{\text{killing by}\\ \text{antibiotic}}}, \tag{D}$$

$$\frac{\mathrm{d}I_F}{\mathrm{d}t} = -\underbrace{\alpha_I A_r I_F E}_{\substack{\text{binding to}\\ \text{host cells}}} + \underbrace{\beta_I\Big(I_{F_{init}}-I_F\Big)}_{\substack{\text{unbinding}\\ \text{from host cells}}}. \tag{E}$$

Inhibitors are conserved in the absence of clearance. Therefore, we are able to neglect the equation for bound inhibitors, I_B (Eq 6), making the substitution $I_B = h(I_{F_{init}} - I_F)$ in Eq E. We also neglect the equation for antibiotic, A (Eq 7), since the antibiotic concentration is held constant. The functions H and η are as defined in Eqs 8 and 9 respectively. All five equations are required when inhibitors are used; however, only Eqs A–D are required in the absence of inhibitors. Further, in the absence of antibiotics, the antibiotic killing terms can be neglected.

The stability properties of the system are summarised in Table 5 and described in detail below.

The untreated scenario has three physically realistic steady-states in Cases A–D. For all cases, the first steady-state,

$(B^*_{FS_1}, B^*_{FR_1}, B^*_{BS_1}, B^*_{BR_1}) = (0,0,0,0)$, corresponds to the complete absence of bacteria and can be classified as an unstable node, with two real positive and two real negative eigenvalues. In the second steady-state, $(B^*_{FS_2}, B^*_{FR_2}, B^*_{BS_2}, B^*_{BR_2}) = (0, B^*_{FR_2}, 0, B^*_{BR_2})$, where $B^*_{FR_2} > 0$ and $B^*_{BR_2} > 0$ in all cases, such that resistant bacteria survive and susceptible bacteria go extinct. This second steady-state is an unstable node in Cases A, B and D, with three real negative eigenvalues and one real positive eigenvalue, while it is an unstable node/spiral in Case C, with two real eigenvalues (one positive and one negative) and a pair of complex conjugate eigenvalues with negative real parts. In the third steady-state, $(B^*_{FS_3}, B^*_{FR_3}, B^*_{BS_3}, B^*_{BR_3}) = (B^*_{FS_3}, 0, B^*_{BS_3}, 0)$, where $B^*_{FS_3} > 0$ and $B^*_{BS_3} > 0$ in all cases, such that susceptible bacteria survive and resistant bacteria go extinct. The third steady-state is a stable node in Cases A, B and D, with four real negative eigenvalues, while it is a stable node/spiral in Case C, with 2 real negative eigenvalues and a pair of complex conjugate eigenvalues with negative real parts.

The antibiotic only scenario has two physically realistic steady-states in Cases A–C and three in Case D. For all cases, the first steady-state, $(B^*_{FS_1}, B^*_{FR_1}, B^*_{BS_1}, B^*_{BR_1}) = (0,0,0,0)$, corresponds to the complete absence of bacteria and can be classified as an unstable node, with one real positive and three real negative eigenvalues in Cases A–C and two real positive and two real negative eigenvalues in Case D. In the second steady-state, $(B^*_{FS_2}, B^*_{FR_2}, B^*_{BS_2}, B^*_{BR_2}) = (0, B^*_{FR_2}, 0, B^*_{BR_2})$, where $B^*_{FR_2} > 0$ and $B^*_{BR_2} > 0$ in all cases, such that resistant bacteria survive and susceptible bacteria go extinct. This second steady-state is a stable node in all cases with four real negative eigenvalues. In the third steady-state (Case D), $(B^*_{FS_3}, B^*_{FR_3}, B^*_{BS_3}, B^*_{BR_3}) = (B^*_{FS_3}, 0, B^*_{BS_3}, 0)$, where $B^*_{FS_3} > 0$ and $B^*_{BS_3} > 0$, such that susceptible bacteria survive and resistant bacteria go extinct. This is an unstable node with one real positive and three real negative eigenvalues.

The inhibitor only scenario has three physically realistic steady-states in Cases A–D. For all cases, the first steady-state, $(B^*_{FS_1}, B^*_{FR_1}, B^*_{BS_1}, B^*_{BR_1}) = (0,0,0,0)$, corresponds to the complete absence of bacteria and can be classified as an unstable node, with two real positive and three real negative eigenvalues in Cases A–C, and three real positive and two real negative eigenvalues in Case D. In the second steady-state, $(B^*_{FS_2}, B^*_{FR_2}, B^*_{BS_2}, B^*_{BR_2}) = (0, B^*_{FR_2}, 0, B^*_{BR_2})$, where $B^*_{FR_2} > 0$ and $B^*_{BR_2} > 0$ in all cases, such that resistant bacteria survive and susceptible bacteria go extinct. This second steady-state is an unstable node with one real positive eigenvalue and four real negative eigenvalues in all cases. In the third steady-state, $(B^*_{FS_3}, B^*_{FR_3}, B^*_{BS_3}, B^*_{BR_3}) = (B^*_{FS_3}, 0, B^*_{BS_3}, 0)$, where $B^*_{FS_3} > 0$ and $B^*_{BS_3} > 0$ in all cases, such that susceptible bacteria survive and resistant bacteria go extinct. The third steady-state is a stable node in Cases B–C with five real negative eigenvalues; however, in Case A this steady-state is unstable, with one real positive eigenvalue and four real negative eigenvalues. Thus, there are no isolated stable steady-states in Case A. Simulations of the time-dependent problem reveal that the system does settle to a steady-state, but that the steady-state attained depends upon the initial conditions. We will address this feature in more detail in a future publication.

The antibiotic and inhibitor scenario has a single physically realistic steady-state in Cases B and C, two in Case A and three in Case D. For all cases, the first steady-state, $(B^*_{FS_1}, B^*_{FR_1}, B^*_{BS_1}, B^*_{BR_1}) = (0,0,0,0)$, corresponds to the complete absence of bacteria. It can be classified as an unstable node, with one real positive and four real negative eigenvalues in Case A, and 2 real positive and 3 real negative eigenvalues in Case D. In Cases B and C this steady-state is a stable node with five real negative eigenvalues. In the second steady-state (Cases A and D), $(B^*_{FS_2}, B^*_{FR_2}, B^*_{BS_2}, B^*_{BR_2}) = (0, B^*_{FR_2}, 0, B^*_{BR_2})$, where $B^*_{FR_2} > 0$ and $B^*_{BR_2} > 0$, such that resistant bacteria survive and susceptible bacteria go extinct. This second steady-state is a stable node in both Cases A and D, with five real negative eigenvalues. In the third steady-state (Case D), $(B^*_{FS_3}, B^*_{FR_3}, B^*_{BS_3}, B^*_{BR_3}) = (B^*_{FS_3}, 0, B^*_{BS_3}, 0)$, where $B^*_{FS_3} > 0$ and $B^*_{BS_3} > 0$, such that susceptible bacteria survive and resistant bacteria go extinct. This is an unstable node with one real positive and four real negative eigenvalues.
