# Supplementary material for: Mathematical model predicts anti-adhesion–antibiotic–debridement combination therapies can clear an antibiotic resistant infection
Source: PLoS Comput Biol. 2019 Jul 23;15(7):e1007211. doi: 10.1371/journal.pcbi.1007211 (PMC6677339; doi:10.1371/journal.pcbi.1007211)
Supplement: S3 Text — (PDF) [file pcbi.1007211.s003.pdf]

Mathematical model predicts anti-adhesion–antibiotic–debridement combination therapies can clear an antibiotic resistant infection
PLOS Computational Biology
S3 Text

Paul A. Roberts*[1,2], Ryan M. Huebinger[3], Emma Keen[2], Anne-Marie Krachler[4] and Sara Jabbari[1,2]

[1]School of Mathematics, University of Birmingham, Edgbaston, Birmingham, United Kingdom
[2]Institute of Microbiology and Infection, School of Biosciences, University of Birmingham, Edgbaston, Birmingham, United Kingdom
[3]Department of Surgery, University of Texas Southwestern Medical Center, Dallas, Texas, United States of America
[4]Department of Microbiology and Molecular Genetics, University of Texas McGovern Medical School at Houston, Houston, Texas, United States of America

Detailed numerical results

In what follows we present additional sensitivity analysis and treatment optimisation results. All results are discussed in the main text, with the exception of the sensitivity analysis results for conjugation and segregation, which are described below.

In all of the results presented in the main text, conjugation and segregation were neglected. Figs F–K below show the effect of varying the conjugation rate, λ, and the segregation rate, ρ, upon the total number of bacteria, B_T, at 1 week (Figs F and I), 4 weeks (Figs G and J) and 1 year (Figs H and K), both in the presence (Figs I–K) and absence (Figs F–H) of antibiotic. The full model (Eqs 1–11) was solved with a constant antibiotic concentration of $A = 0$ or 8 μg ml^{-1} and without inhibitors, where $\tilde{\psi}_{Bac} = 0$ hr^{-1}, $\tilde{\psi}_I = 0$ hr^{-1} and $\omega = 1$. Conjugation and segregation were varied in the ranges $\lambda \in [0, 1 \times 10^{-6}]$ cm^{3}cell^{-1}hr^{-1} and $\rho \in [0, 3 \times 10^3]$ hr^{-1}. We have exceeded the ranges suggested in Parameter fitting and justification as this was necessary in order to obtain a significant change in model output.

In the absence of antibiotics the system is relatively insensitive to variations in these parameters at all times considered and in all four cases (A–D). Increasing ρ mildly increases B_T for all cases and times considered, while increasing λ decreases B_T in Cases A–D at 4 weeks and 1 year and in Case A at 1 week, while it increases B_T in Cases B and D at 1 week and has a non-monotonic effect in Case C at 1 week. In the presence of antibiotics, the situation is reversed such that increasing ρ decreases B_T, while increasing λ increases B_T. This effect is relatively insignificant in Cases A–D at 1 year and in Cases A, B and D at 4 weeks, but is significant in Case C at 4 weeks and in Cases A–D at 1 week, being much more sensitive to λ than to ρ in these cases. However, the sensitivity is only present within the exaggerated parameter ranges. Therefore, within realistic ranges, the system is insensitive to variation in the rates of conjugation and segregation. For this reason and the reasons stated in Parameter fitting and justification, we neglect conjugation and segregation in the results presented in the main text.

*Corresponding author
E-mail address: p.a.roberts@univ.oxon.org (PAR)

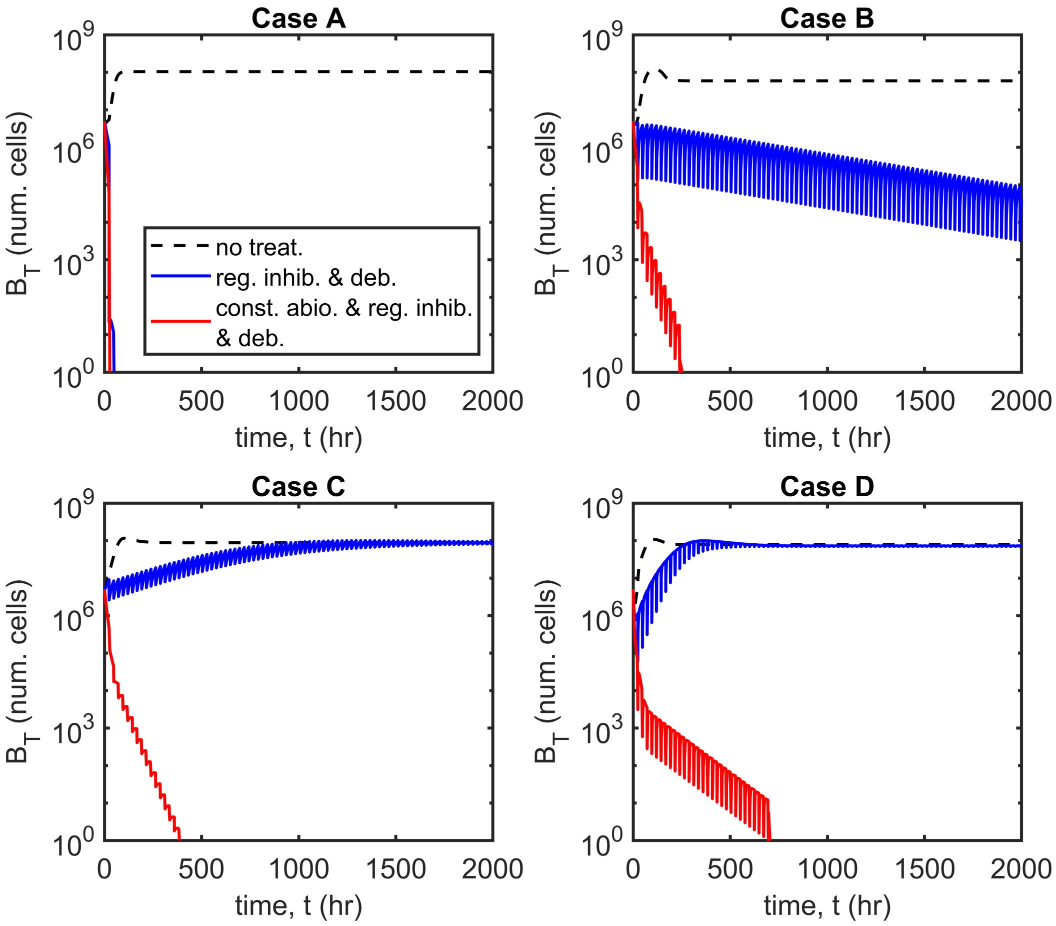

Figure A: **Dynamic simulations comparing treatment with and without antibiotic.** The total number of bacteria, B_T, is shown over time for the untreated scenario and for two treatment strategies: regular inhibitor dosing with regular debridement, with and without a constant antibiotic dose. Note the $\log_{10}$ scale on the y-axis. Inhibitors and debridement alone are only capable of clearing the bacterial infection in Case A. However, when combined with a constant antibiotic dose, the bacterial infection can be cleared in all four cases. Eqs 1–11 were solved using `ode15s`. Parameter values: $\lambda = 0$ cm^{3}cell^{-1}hr^{-1}, $\rho = 0$ hr^{-1} and $\omega = 1$. Inhibitor doses: $6.12{\times}10^7$ inhib. cm^{-3}, constant antibiotic scenario: $A = 8$ μg cm^{-3}. See Tables 2–4 for the remaining parameter values.

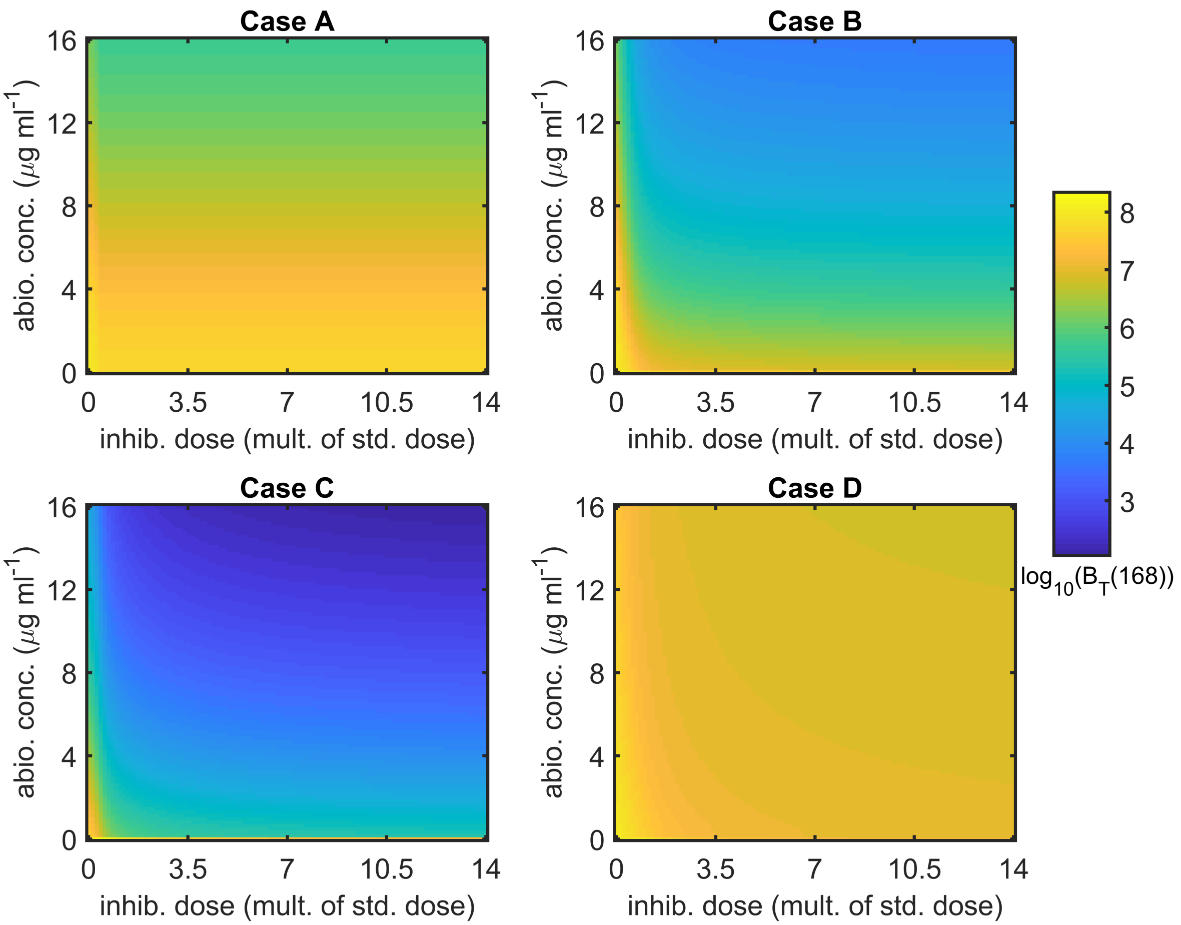

Figure B: **Sensitivity analysis for antibiotic and inhibitor doses — 1 week.** The $\log_{10}$ of the total number of bacteria at 1 week (168 hr), $B_T(168)$, is plotted for a range of antibiotic and inhibitor doses. Note that inhibitor treatments are plotted as multiples of the standard dose (6.12×10^7 inhib. cm^{-3}). Treatment is most effective in Cases B and C, reducing the bacterial burden by several orders of magnitude for sufficiently high antibiotic and inhibitor doses. Eqs 1–11 were solved using `ode15s` and with a constant antibiotic dose. Parameter values: $\tilde{\psi}_{Bac} = 0$ hr^{-1}, $\tilde{\psi}_I = 0$ hr^{-1}, $\lambda = 0$ cm^{3}cell^{-1}hr^{-1}, $\rho = 0$ hr^{-1} and $\omega = 1$. See Tables 2–4 for the remaining parameter values.

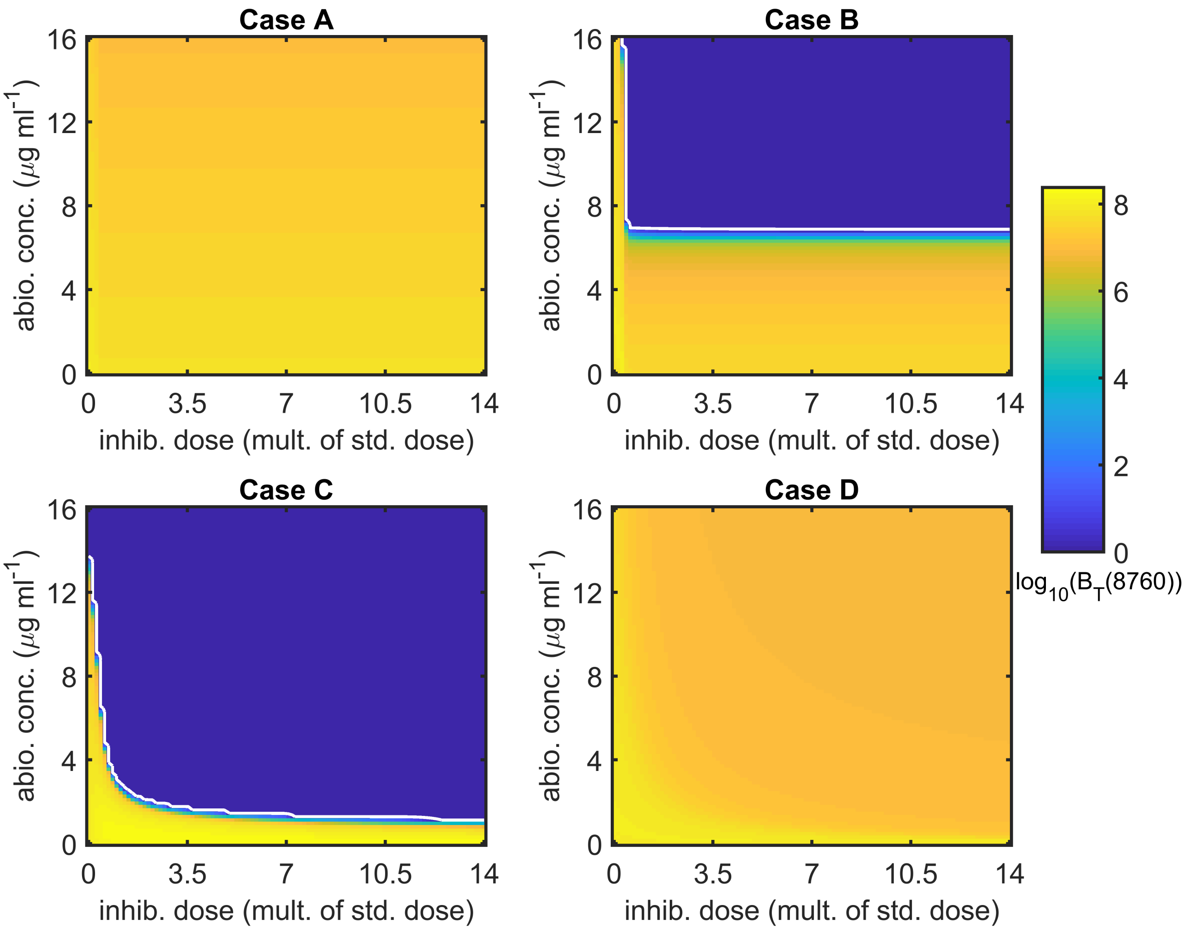

Figure C: **Sensitivity analysis for antibiotic and inhibitor doses — 1 year.** The $\log_{10}$ of the total number of bacteria at 1 year (8760 hr), $B_T(8760)$, is plotted for a range of antibiotic and inhibitor doses. Note that inhibitor treatments are plotted as multiples of the standard dose (6.12×10^7 inhib. cm^{-3}) and that values of $B_T(8760) < 1$ are plotted as $B_T(8760) = 1$ to maximise visual clarity. The white curves are the contours along which $B_T(8760) = 1$; hence, $B_T(8760) < 1$ above-right of these contours. The effect of treatment is relatively minor in Cases A and D; however, the bacterial burden may be eliminated for sufficiently high antibiotic and inhibitor doses in Cases B and C. Eqs 1–11 were solved using `ode15s` and with a constant antibiotic dose. Parameter values: $\tilde{\psi}_{Bac} = 0$ hr^{-1}, $\tilde{\psi}_I = 0$ hr^{-1}, $\lambda = 0$ cm^{3}cell^{-1}hr^{-1}, $\rho = 0$ hr^{-1} and $\omega = 1$. See Tables 2–4 for the remaining parameter values.

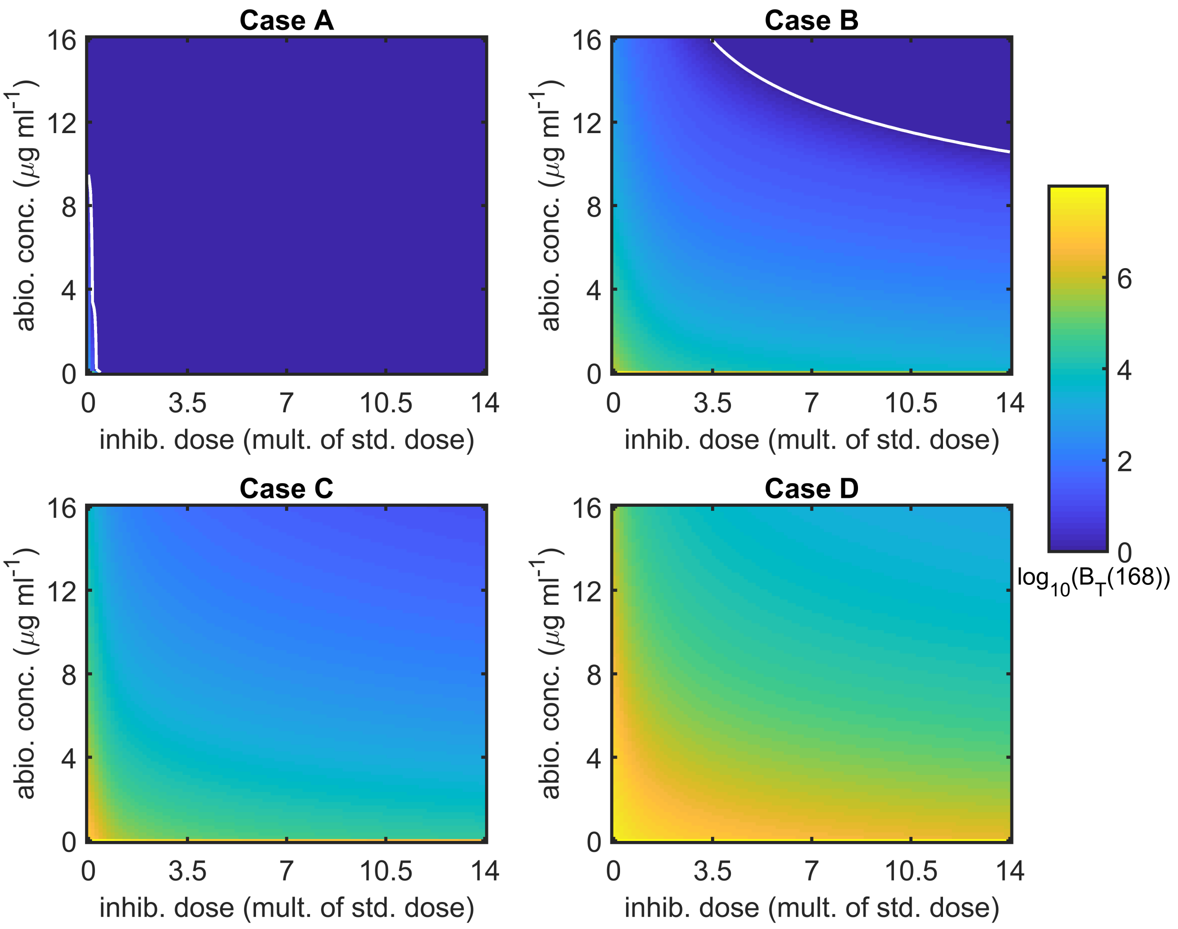

Figure D: **Sensitivity analysis for antibiotic and inhibitor doses with debridement — 1 week.** The $\log_{10}$ of the total number of bacteria at 1 week (168 hr), $B_T(168)$, is plotted for a range of antibiotic and inhibitor doses. Note that inhibitor treatments are plotted as multiples of the standard dose (6.12×10^7 inhib. cm^{-3}) and that values of $B_T(168) < 1$ are plotted as $B_T(168) = 1$ to maximise visual clarity. The white curves are the contours along which $B_T(168) = 1$; hence, $B_T(168) < 1$ above-right of these contours. Treatment is most effective in Case A, eliminating the bacterial burden for very low antibiotic and inhibitor doses and is also highly effective in Case B, eliminating the bacterial burden for higher antibiotic and inhibitor doses. The bacterial burden may also be reduced by several orders of magnitude in Cases C and D. Eqs 1–11 were solved using `ode15s` and with a constant antibiotic dose. Debridement takes place at the start of each day, occurring for the first time at $t = 24$ hr, effecting the removal of all free bacteria and inhibitors. Parameter values: $\lambda = 0$ cm^{3}cell^{-1}hr^{-1}, $\rho = 0$ hr^{-1} and $\omega = 1$. See Tables 2–4 for the remaining parameter values.

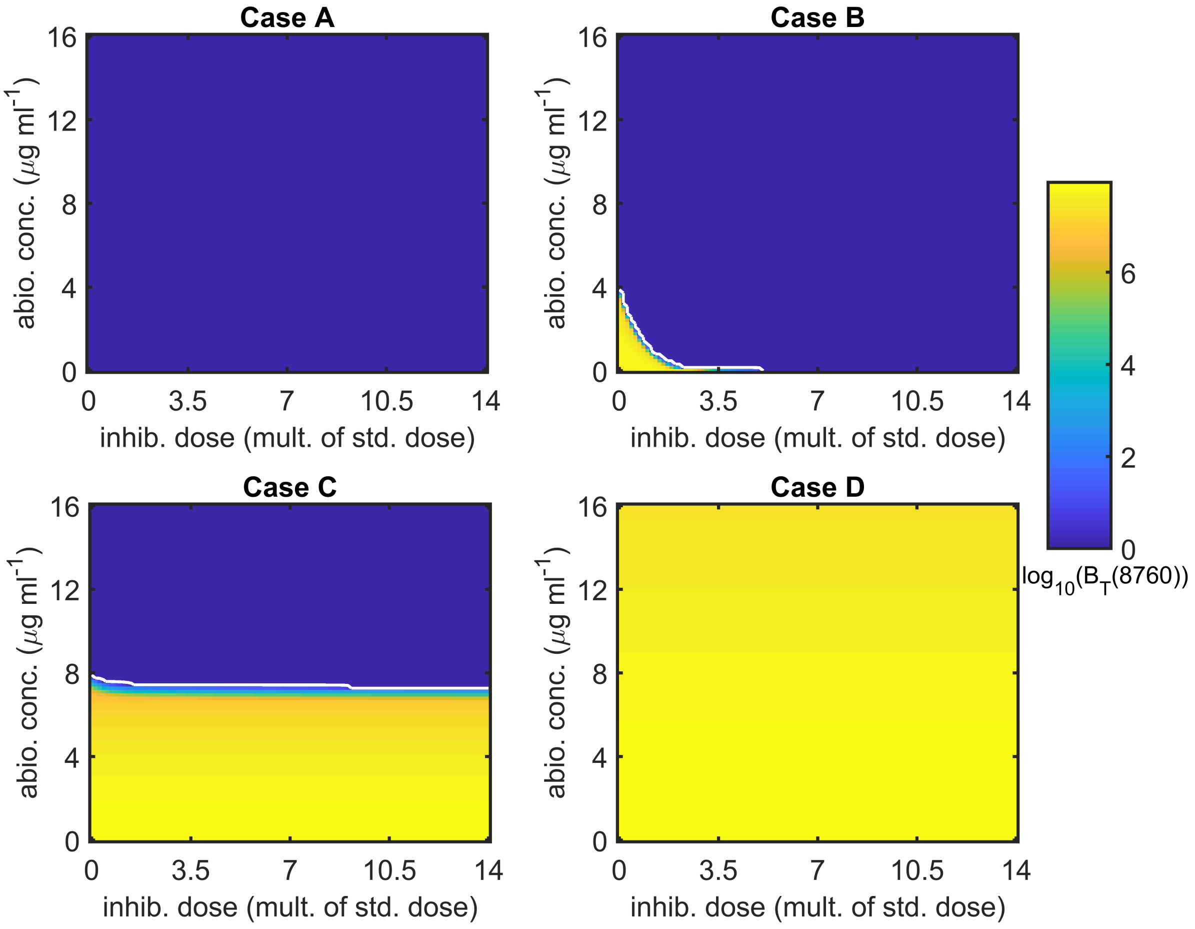

Figure E: **Sensitivity analysis for antibiotic and inhibitor doses with debridement — 1 year.** The $\log_{10}$ of the total number of bacteria at 1 year (8760 hr), $B_T(8760)$, is plotted for a range of antibiotic and inhibitor doses. Note that inhibitor treatments are plotted as multiples of the standard dose (6.12×10^7 inhib. cm^{-3}) and that values of $B_T(8760) < 1$ are plotted as $B_T(8760) = 1$ to maximise visual clarity. The white curves are the contours along which $B_T(8760) = 1$; hence, $B_T(8760) < 1$ above-right of these contours. Treatment is most effective in Case A, where debridement alone is sufficient to clear an infection, and can also eliminate the bacterial population in Cases B and C for sufficiently high antibiotic and inhibitor doses. By contrast, treatment has little effect in Case D. Eqs 1–11 were solved using `ode15s` and with a constant antibiotic dose. Debridement takes place at the start of each day, occurring for the first time at $t = 24$ hr, effecting the removal of all free bacteria and inhibitors. Parameter values: $\lambda = 0$ $\text{cm}^3\text{cell}^{-1}\text{hr}^{-1}$, $\rho = 0$ hr^{-1} and $\omega = 1$. See Tables 2–4 for the remaining parameter values.

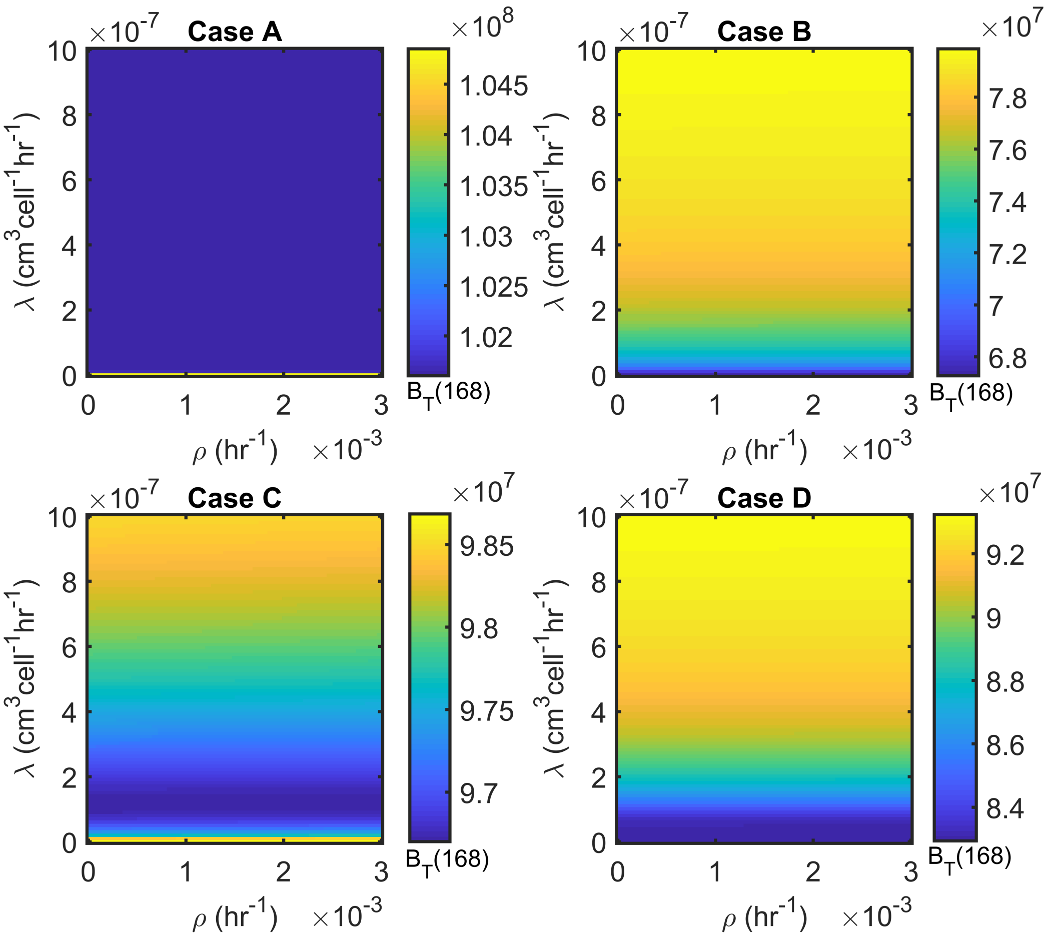

Figure F: **Sensitivity analysis for conjugation and segregation rates in the absence of antibiotic — 1 week.** The total number of bacteria at 1 week (168 hr), $B_T(168)$, is plotted for a range of conjugation rates, λ, and segregation rates, ρ. The system is relatively insensitive to variation in these parameters in all cases. Variation in ρ has little discernible effect, while increasing λ may increase or decrease B_T depending upon the parameter set and the range within which it is varied. Eqs 1–11 were solved using `ode15s`, without antibiotic or inhibitors. Parameter values: $\tilde{\psi}_{Bac} = 0$ hr^{-1}, $\tilde{\psi}_I = 0$ hr^{-1} and $\omega = 1$. See Tables 2–4 for the remaining parameter values.

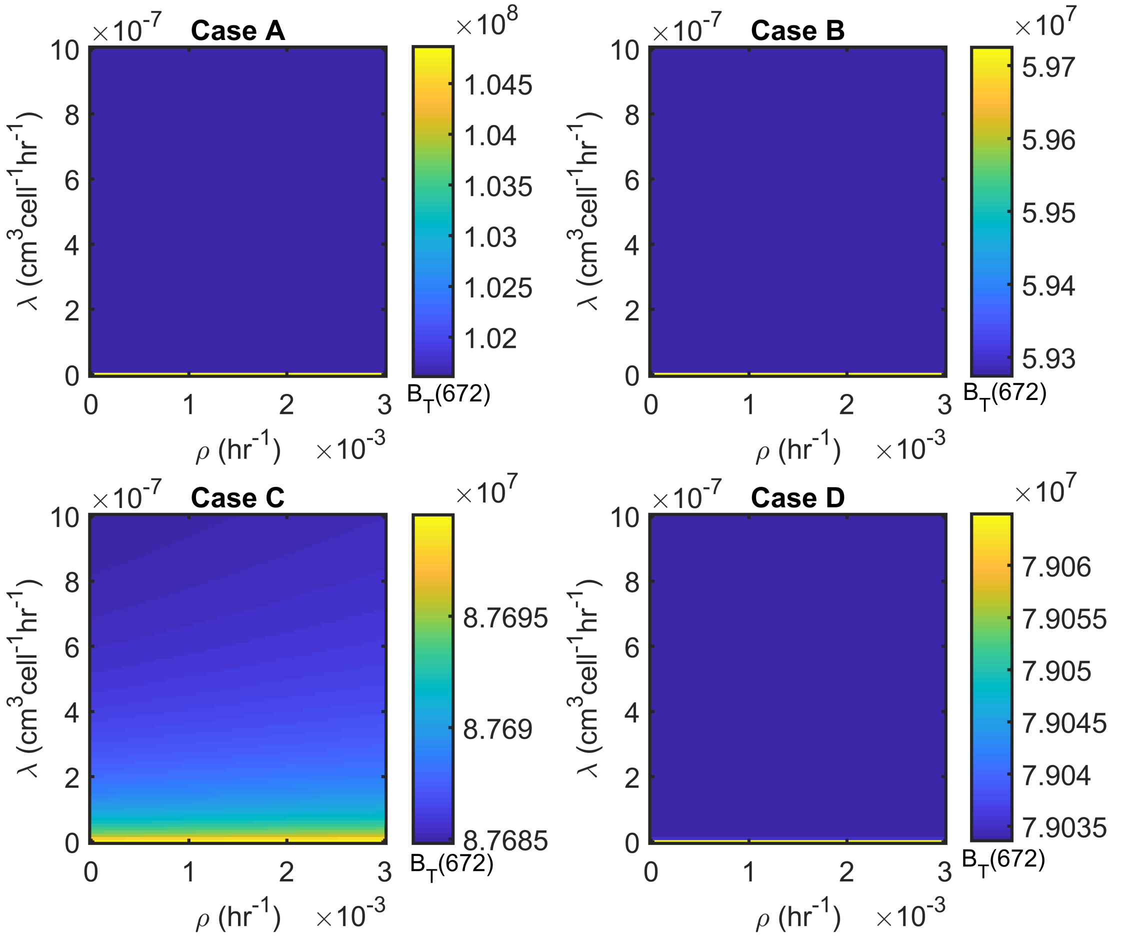

Figure G: **Sensitivity analysis for conjugation and segregation rates in the absence of antibiotic — 4 weeks.** The total number of bacteria at 4 weeks (672 hr), $B_T(672)$, is plotted for a range of conjugation rates, λ, and segregation rates, ρ. The system is relatively insensitive to variation in these parameters in all cases. Variation in ρ has little discernible effect, while B_T decreases with increasing λ. Eqs 1–11 were solved using `ode15s`, without antibiotic or inhibitors. Parameter values: $\tilde{\psi}_{Bac} = 0$ hr^{-1}, $\tilde{\psi}_I = 0$ hr^{-1} and $\omega = 1$. See Tables 2–4 for the remaining parameter values.

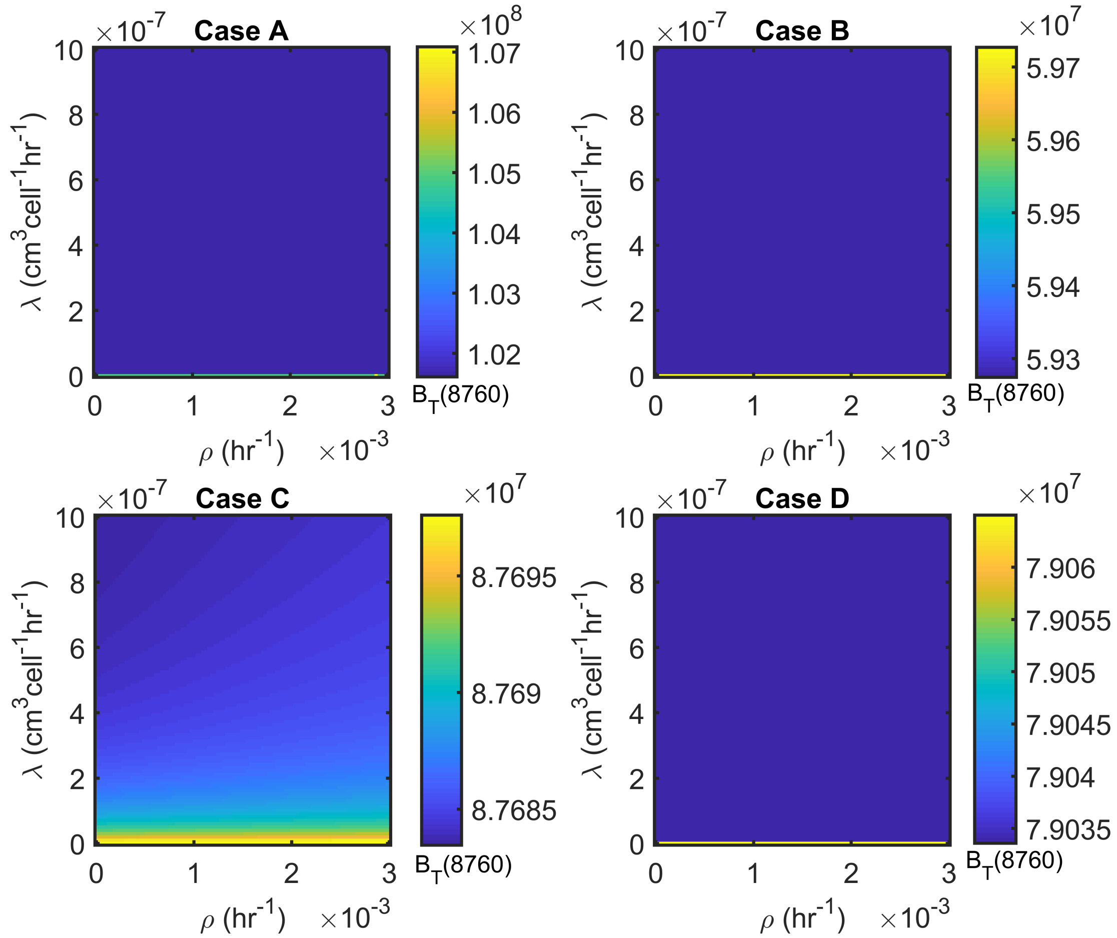

Figure H: **Sensitivity analysis for conjugation and segregation rates in the absence of antibiotic — 1 year.** The total number of bacteria at 1 year (8760 hr), $B_T(8760)$, is plotted for a range of conjugation rates, λ, and segregation rates, ρ. The system is relatively insensitive to variation in these parameters in all cases. Variation in ρ has little discernible effect, while B_T decreases with increasing λ. Eqs 1–11 were solved using `ode15s`, without antibiotic or inhibitors. Parameter values: $\tilde{\psi}_{Bac} = 0$ hr^{-1}, $\tilde{\psi}_I = 0$ hr^{-1} and $\omega = 1$. See Tables 2–4 for the remaining parameter values.

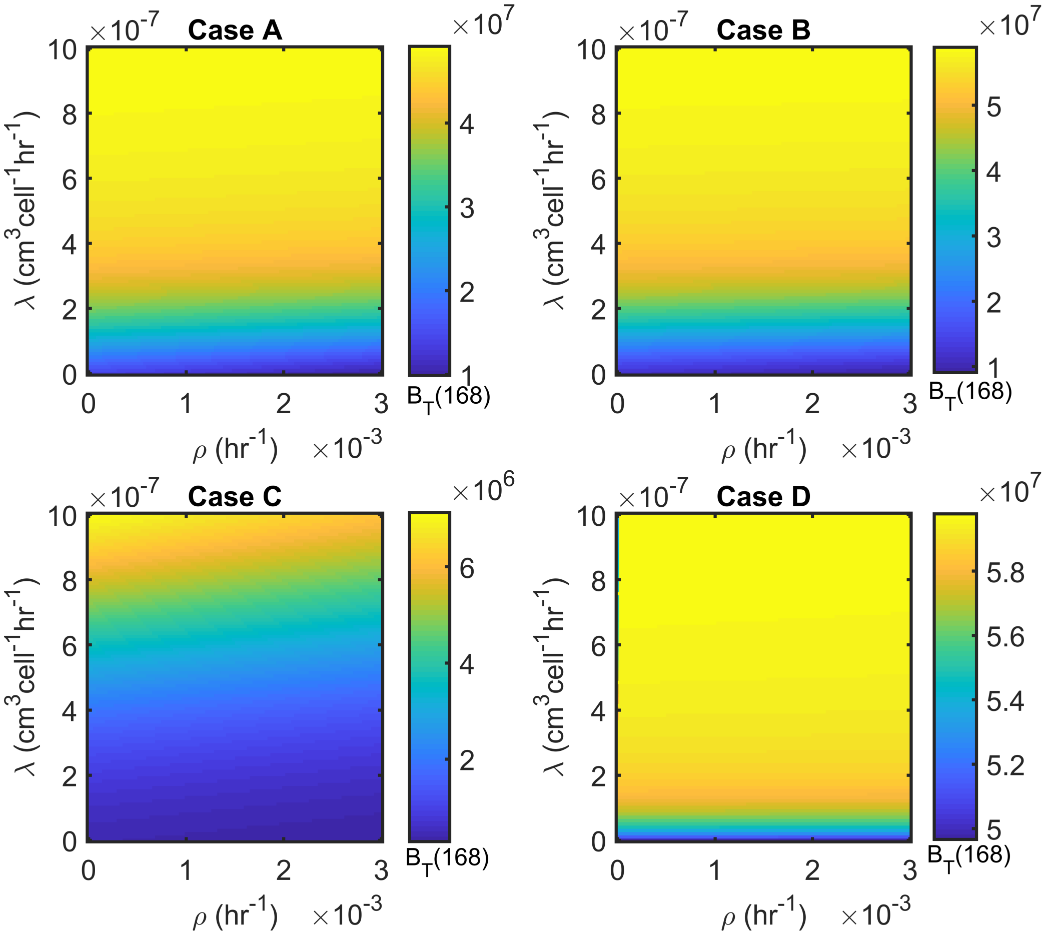

Figure I: **Sensitivity analysis for conjugation and segregation rates in the presence of antibiotic — 1 week.** The total number of bacteria at 1 week (168 hr), $B_T(168)$, is plotted for a range of conjugation rates, λ, and segregation rates, ρ. The system is relatively insensitive to variation in ρ; however, it is sensitive to variation in λ for all parameter sets, especially for Cases A–C. In all cases, increasing the segregation rate decreases the bacterial burden, while increasing the conjugation rate increases the bacterial burden. Eqs 1–11 were solved using `ode15s`, with a constant antibiotic dose and without inhibitors. Parameter values: $A = 8\ \mu$g ml^{-1}, $\tilde{\psi}_{Bac} = 0$ hr^{-1}, $\tilde{\psi}_I = 0$ hr^{-1} and $\omega = 1$. See Tables 2–4 for the remaining parameter values.

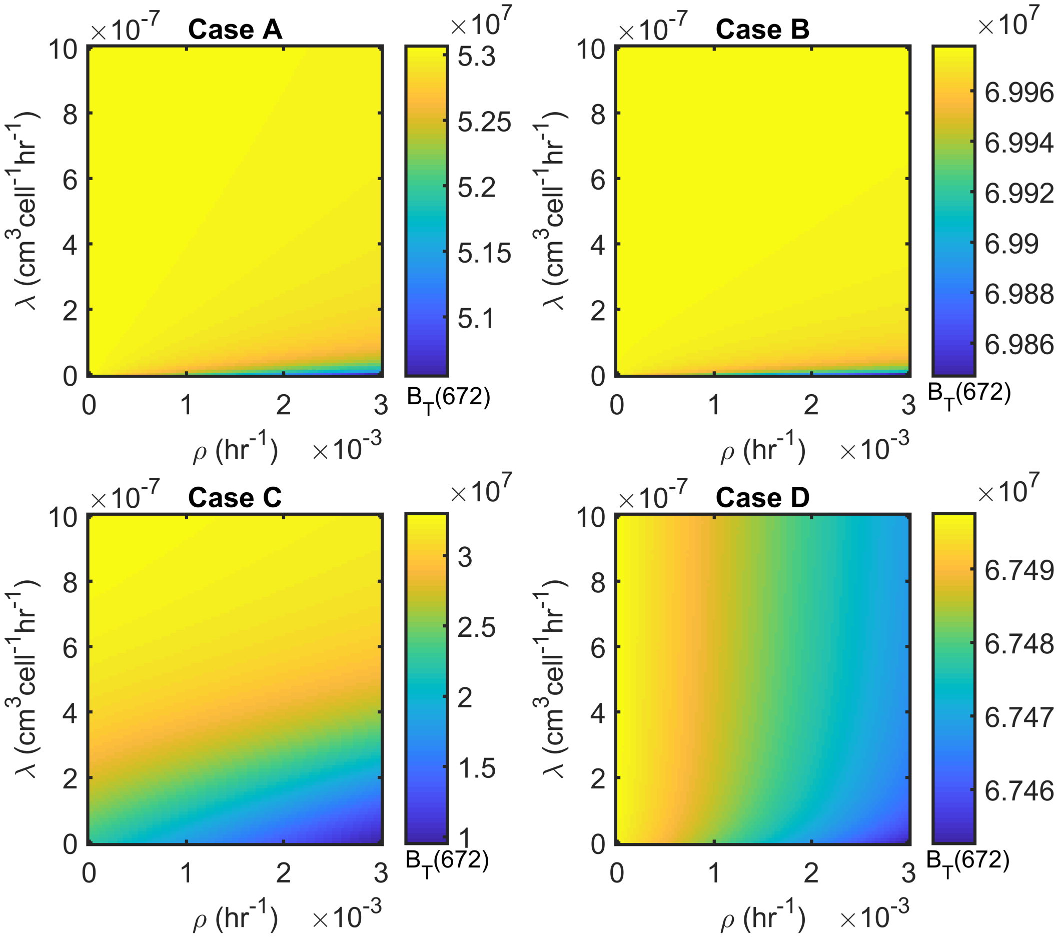

Figure J: **Sensitivity analysis for conjugation and segregation rates in the presence of antibiotic — 4 weeks.** The total number of bacteria at 4 weeks (672 hr), $B_T(672)$, is plotted for a range of conjugation rates, λ, and segregation rates, ρ. The system is relatively insensitive to variation in these parameters in Cases A, B and D, while the effect is more pronounced in Case C. In all cases, increasing the segregation rate decreases the bacterial burden, while increasing the conjugation rate increases the bacterial burden. Eqs 1–11 were solved using ode15s, with a constant antibiotic dose and without inhibitors. Parameter values: $A = 8\ \mu$g ml^{-1}, $\tilde{\psi}_{Bac} = 0$ hr^{-1}, $\tilde{\psi}_I = 0$ hr^{-1} and $\omega = 1$. See Tables 2–4 for the remaining parameter values.

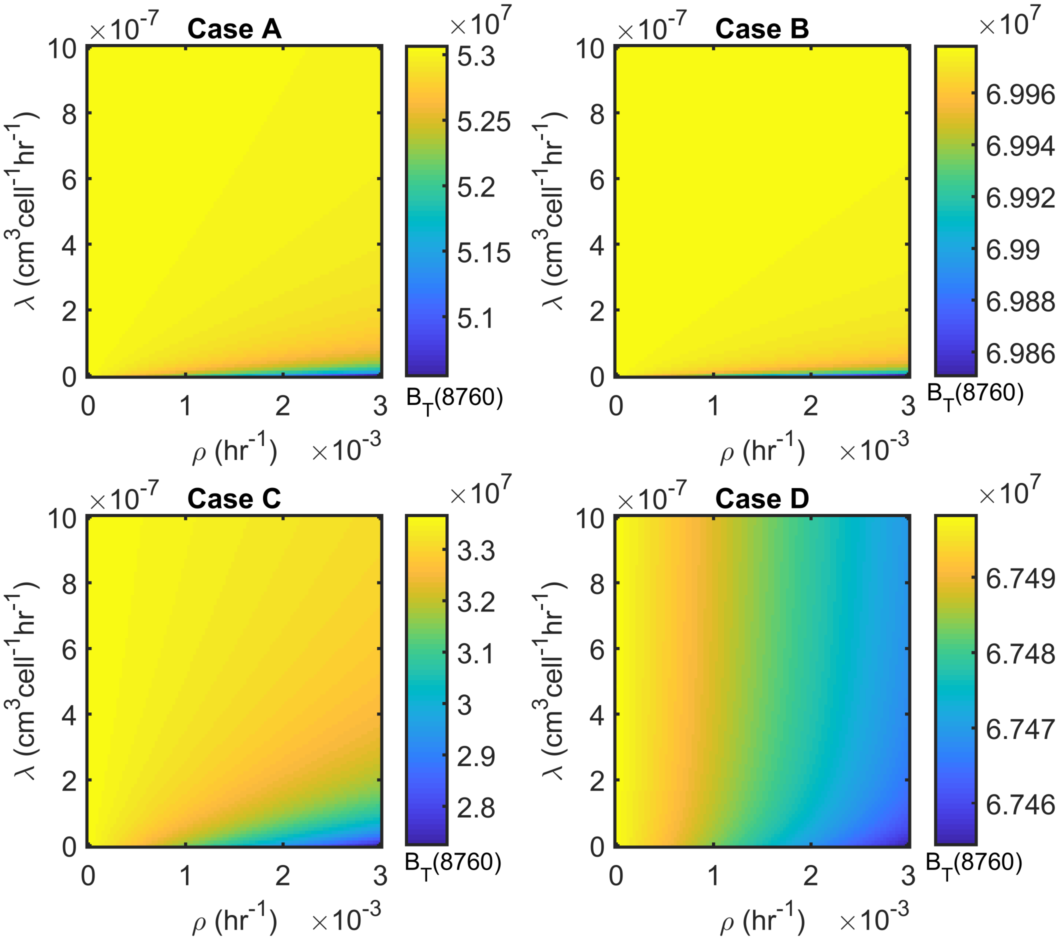

Figure K: **Sensitivity analysis for conjugation and segregation rates in the presence of antibiotic — 1 year.** The total number of bacteria at 1 year (8760 hr), $B_T(8760)$, is plotted for a range of conjugation rates, λ, and segregation rates, ρ. The system is relatively insensitive to variation in these parameters for all parameter sets. In all cases, increasing the segregation rate decreases the bacterial burden, while increasing the conjugation rate increases the bacterial burden. Eqs 1–11 were solved using `ode15s`, with a constant antibiotic dose and without inhibitors. Parameter values: $A = 8\ \mu$g ml^{-1}, $\tilde{\psi}_{Bac} = 0$ hr^{-1}, $\tilde{\psi}_I = 0$ hr^{-1} and $\omega = 1$. See Tables 2–4 for the remaining parameter values.

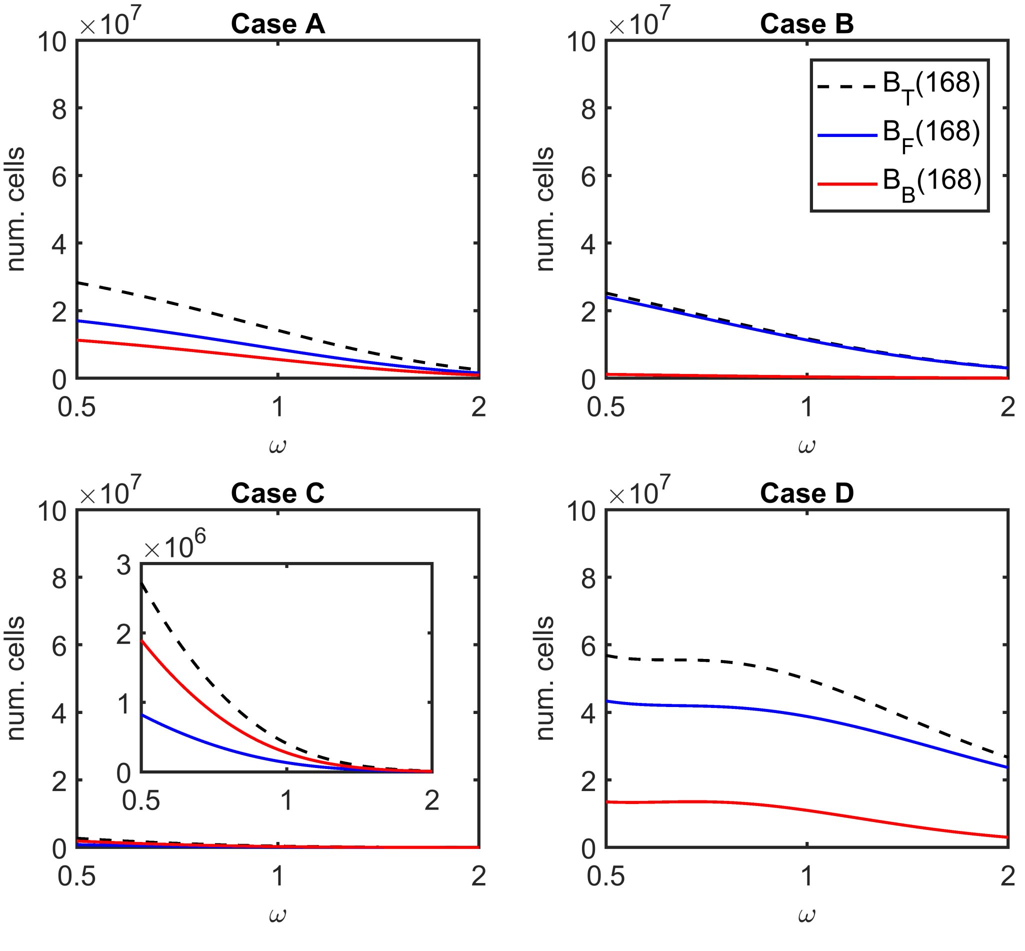

Figure L: **Sensitivity analysis for the factor difference in antibiotic potency against bound bacteria compared with free bacteria — 1 week.** The total number of bacteria, together with the numbers of free and bound bacteria at 1 week (168 hr), $B_T(168)$, $B_F(168)$ and $B_B(168)$ respectively, are plotted for a range of values of the potency factor, ω. Note the $\log_2$ scale on the x-axis. In almost all cases, $B_T(168)$, $B_F(168)$ and $B_B(168)$ decrease monotonically with increasing ω, the only exception being $B_B(168)$ in Case D, which increases for $\omega \in (0.55, 0.68)$. The inset graph for Case C magnifies the y-dimension to clarify the system behaviour. Eqs 1–11 were solved using `ode15s`, with a constant antibiotic dose and without inhibitors. Parameter values: $A = 8$ μg ml^{-1}, $\tilde{\psi}_{Bac} = 0$ hr^{-1}, $\tilde{\psi}_I = 0$ hr^{-1}, $\lambda = 0$ cm^{3}cell^{-1}hr^{-1} and $\rho = 0$ hr^{-1}. See Tables 2–4 for the remaining parameter values.

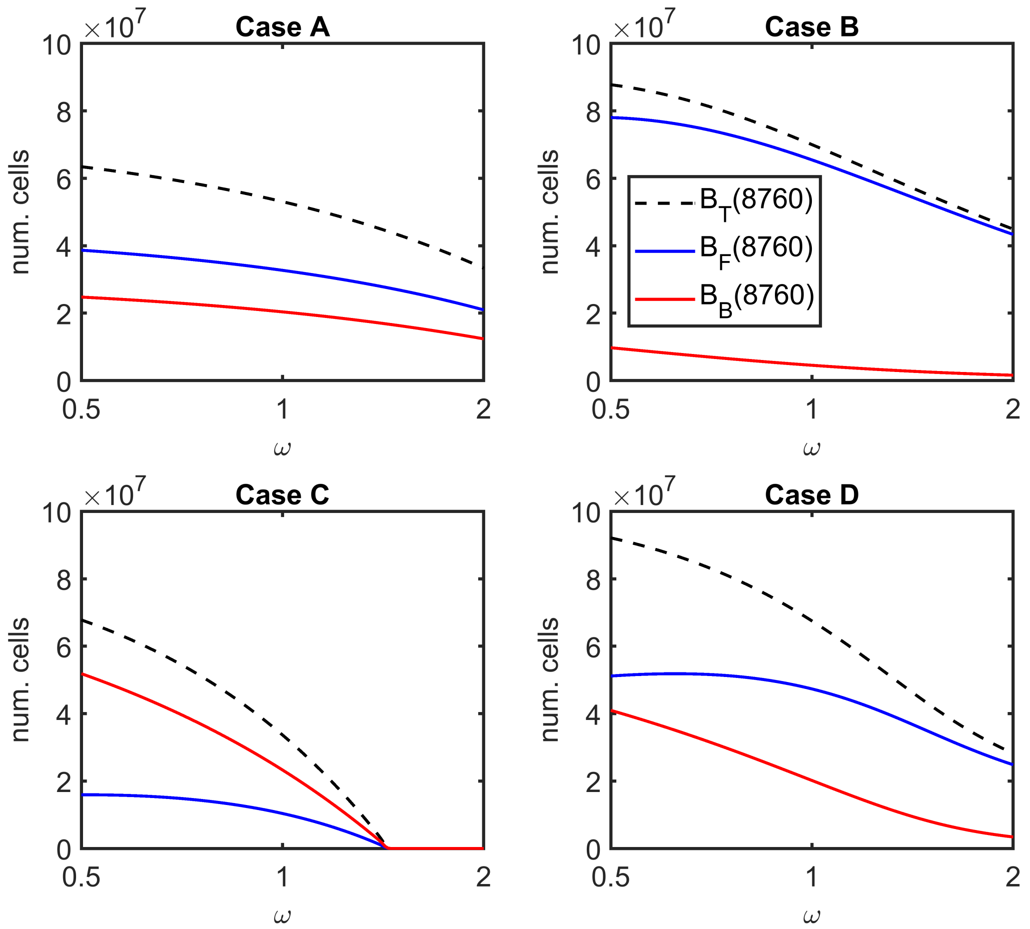

Figure M: **Sensitivity analysis for the factor difference in antibiotic potency against bound bacteria compared with free bacteria — 1 year.** The total number of bacteria, together with the numbers of free and bound bacteria at 1 year (8760 hr), $B_T(8760)$, $B_F(8760)$ and $B_B(8760)$ respectively, are plotted for a range of values of the potency factor, ω. Note the $\log_2$ scale on the x-axis. In almost all cases, $B_T(8760)$, $B_F(8760)$ and $B_B(8760)$ decrease monotonically with increasing ω, the only exception being $B_F(8760)$ in Case D, which increases initially. The effect is particularly pronounced in Case C, where the bacterial burden is eliminated ($B_T < 1$) as ω approaches 2. Eqs 1–11 were solved using ode15s, with a constant antibiotic dose and without inhibitors. Parameter values: $A = 8\ \mu$g ml^{-1}, $\tilde{\psi}_{Bac} = 0$ hr^{-1}, $\tilde{\psi}_I = 0$ hr^{-1}, $\lambda = 0$ cm^{3}cell^{-1}hr^{-1} and $\rho = 0$ hr^{-1}. See Tables 2–4 for the remaining parameter values.
